# Mechanism of a COVID-19 nanoparticle vaccine candidate that elicits a broadly neutralizing antibody response to SARS-CoV-2 variants

**DOI:** 10.1126/sciadv.abj3107

**Published:** 2021-10-20

**Authors:** Yi-Nan Zhang, Jennifer Paynter, Cindy Sou, Tatiana Fourfouris, Ying Wang, Ciril Abraham, Timothy Ngo, Yi Zhang, Linling He, Jiang Zhu

**Affiliations:** 1 Department of Integrative Structural and Computational Biology, The Scripps Research Institute, La Jolla, CA 92037, USA.; 2 Fels Institute for Cancer Research and Molecular Biology, Temple University, Philadelphia, PA 19140, USA.; 3 Department of Microbiology and Immunology, Temple University, Philadelphia, PA 19140, USA.; 4 Department of Immunology and Microbiology, The Scripps Research Institute, La Jolla, CA 92037, USA.

## Abstract

Vaccines that induce potent neutralizing antibody (NAb) responses against emerging variants of severe acute respiratory syndrome coronavirus 2 (SARS-CoV-2) are essential for combating the coronavirus disease 2019 (COVID-19) pandemic. We demonstrated that mouse plasma induced by self-assembling protein nanoparticles (SApNPs) that present 20 rationally designed S2GΔHR2 spikes of the ancestral Wuhan-Hu-1 strain can neutralize the B.1.1.7, B.1.351, P.1, and B.1.617 variants with comparable potency. The adjuvant effect on vaccine-induced immunity was investigated by testing 16 formulations for the multilayered I3-01v9 SApNP. Using single-cell sorting, monoclonal antibodies (mAbs) with diverse neutralization breadth and potency were isolated from mice immunized with the receptor binding domain (RBD), S2GΔHR2 spike, and SApNP vaccines. The mechanism of vaccine-induced immunity was examined in the mouse model. Compared with the soluble spike, the I3-01v9 SApNP showed sixfold longer retention, fourfold greater presentation on follicular dendritic cell dendrites, and fivefold stronger germinal center reactions in lymph node follicles.

## Teaser

With a well-defined mechanism, spike nanoparticles offer an effective vaccine solution to SARS-CoV-2 variants.

## INTRODUCTION

The coronavirus disease 2019 (COVID-19) pandemic has led to more than 231 million infection cases and 4.7 million deaths globally. Antibody responses to severe acute respiratory syndrome coronavirus 2 (SARS-CoV-2) spike antigens can be sustained for several months in most patients with COVID-19 after infection (*1*–*4*). However, recently identified variants of concern (VOCs) exhibit higher transmissibility and resistance to prior immunity as SARS-CoV-2 continues to adapt to the human host (*5*, *6*). One such variant, B.1.1.7 (World Health Organization classification: Alpha), emerged from southeast England in October 2020 and accounted for two-thirds of new infections in London in December 2020, with a higher transmission rate (43 to 90%) and risk of mortality (32 to 104%) than previously circulating strains (*7*, *8*). Other variants, such as B.1.351 (Beta) and P.1 (Gamma), also became prevalent in three provinces in South Africa and Manaus, Brazil, respectively (*6*, *9*, *10*). The B.1.617.2 (Delta) variant, which was initially identified in India, has become a dominant strain in many countries (*11*, *12*) and responsible for most of the new COVID-19 cases. This variant was found to be ~60% more transmissible than the highly infectious B.1.1.7 variant (*12*). The rise of SARS-CoV-2 VOCs and their rapid spread worldwide result in more infection cases, hospitalizations, and potentially more deaths, further straining health care resources (*10*).

To date, eight COVID-19 vaccines have been approved for emergency use in humans, with more than 100 candidates assessed in various phases of clinical trials (*13*). With the exception of inactivated whole virion vaccines, diverse platforms have been used to deliver the recombinant SARS-CoV-2 spike, such as mRNA-encapsulating liposomes (e.g., BNT162b2 and mRNA-1273), adenovirus vectors [e.g., ChAdOx1 nCoV-19 (AZD1222), CTII-nCoV, Sputnik V, and Ad26.COV2.S], and micelle-attached spikes (e.g., NVX-CoV2373). These vaccines demonstrated 65 to 96% efficacy in phase 3 trials, with lower morbidity and mortality associated with COVID-19 disease (*14*–*19*). However, a notable loss of vaccine efficacy against new SARS-CoV-2 variants was reported, likely caused by spike mutations in the receptor binding domain (RBD; e.g., K417N, E484K, and N501Y), N-terminal domain (NTD; e.g., L18F, D80A, D215G, and Δ242-244), and other regions that are critical to spike stability and function (e.g., D614G and P681R) (*6*, *11*, *20*–*25*). Among circulating VOCs, the B.1.351 lineage appeared to be most resistant to neutralization by convalescent plasma (9.4-fold) and vaccine sera (10.3- to 12.4-fold) (*26*), whereas a lesser degree of reduction was observed for an early variant, B.1.1.7 (*27*–*29*). On the basis of these findings, it was suggested that vaccines would need to be updated periodically to maintain protection against rapidly evolving SARS-CoV-2 (*30*–*32*). However, in a recent study, convalescent sera from B.1.351- or P.1-infected individuals showed a more visible reduction of B.1.617.2 neutralization than convalescent sera from individuals infected with early pandemic strains (*33*). Together, these issues raise the concern that herd immunity may be difficult to achieve, highlighting the necessity of developing vaccines that can elicit a broadly neutralizing antibody (bNAb) response to current and emerging variants (*25*, *31*). As previously reported (*34*–*38*), the production of a bNAb response relies on long-lived germinal center (GC) reactions to activate precursor B cells, stimulate affinity maturation, and form long-term immune memory. In particular, antigen retention and presentation within lymph node follicles are key to the induction of long-lived GC reactions (*34*, *36*, *39*) and should be considered in the development of bNAb-producing vaccines (*40*).

We previously investigated the cause of SARS-CoV-2 spike metastability and rationally designed the S2GΔHR2 spike, which was displayed on three self-assembling protein nanoparticle (SApNP) platforms, including ferritin (FR) 24-mer and multilayered E2p and I3-01v9 60-mers, as COVID-19 vaccine candidates (*41*). In the present study, we investigated the vaccine-induced NAb response to SARS-CoV-2 VOCs and the mechanism by which SApNP vaccines (e.g., I3-01v9) generate such a response. We first examined the neutralizing activity of mouse plasma from our previous study (*41*) against four representative SARS-CoV-2 variants, B.1.1.7, B.1.351, P.1, and B.1.617_Rec_, which was derived from a detailed analysis of the B.1.617 lineage (*11*) and shares key spike mutations with VOC B.1.617.2. Mouse plasma induced by the S2GΔHR2 spike-presenting I3-01v9 SApNP potently neutralized all four variants with comparable titers to the wild-type strain, Wuhan-Hu-1. When a different injection route was tested in mouse immunization, E2p and I3-01v9 SApNPs sustained neutralizing titers against the four variants, even at a low dosage of 3.3 μg, whereas a significant reduction of plasma neutralization was observed for the soluble spike. Next, we examined the adjuvant effect on vaccine-induced humoral and T cell responses for the I3-01v9 SApNP. While detectable plasma neutralization was observed for the nonadjuvanted I3-01v9 group, conventional adjuvants, such as aluminum hydroxide (AH) and aluminum phosphate (AP), boosted the titers by 8.6- to 11.3-fold (or 9.6 to 12.3 times). Adjuvants that target the stimulator of interferon genes (STING) and Toll-like receptor 9 (TLR9) pathways enhanced neutralization by 21- to 35-fold, alone or combined with AP, in addition to a T helper 1 (T_H_1)–biased cellular response. We then performed antigen-specific single-cell sorting and isolated 20 monoclonal antibodies (mAbs) from RBD, spike, and I3-01v9 SApNP-immunized mice. These mAbs were derived from diverse B cell lineages, of which some neutralized the wild-type Wuhan-Hu-1 strain and four variants with equivalent potency. Last, we investigated how SApNPs behave in lymph nodes and induce GCs by characterizing vaccine delivery and immunological responses at the intraorgan, intracellular, and intercellular levels in mice. The I3-01v9 SApNP showed sixfold longer retention, fourfold greater presentation on follicular dendritic cell (FDC) dendrites, and fivefold higher GC reactions than the soluble spike. Intact SApNPs in lymph node tissues could be visualized by transmission electron microscopy (TEM). Our study thus demonstrates that a spike-presenting SApNP vaccine derived from the ancestral SARS-CoV-2 strain (Wuhan-Hu-1) may confer broad protection against emerging variants.

## RESULTS

### Spike and SApNP vaccine-induced neutralizing responses to SARS-CoV-2 variants

**Fig. 1.**
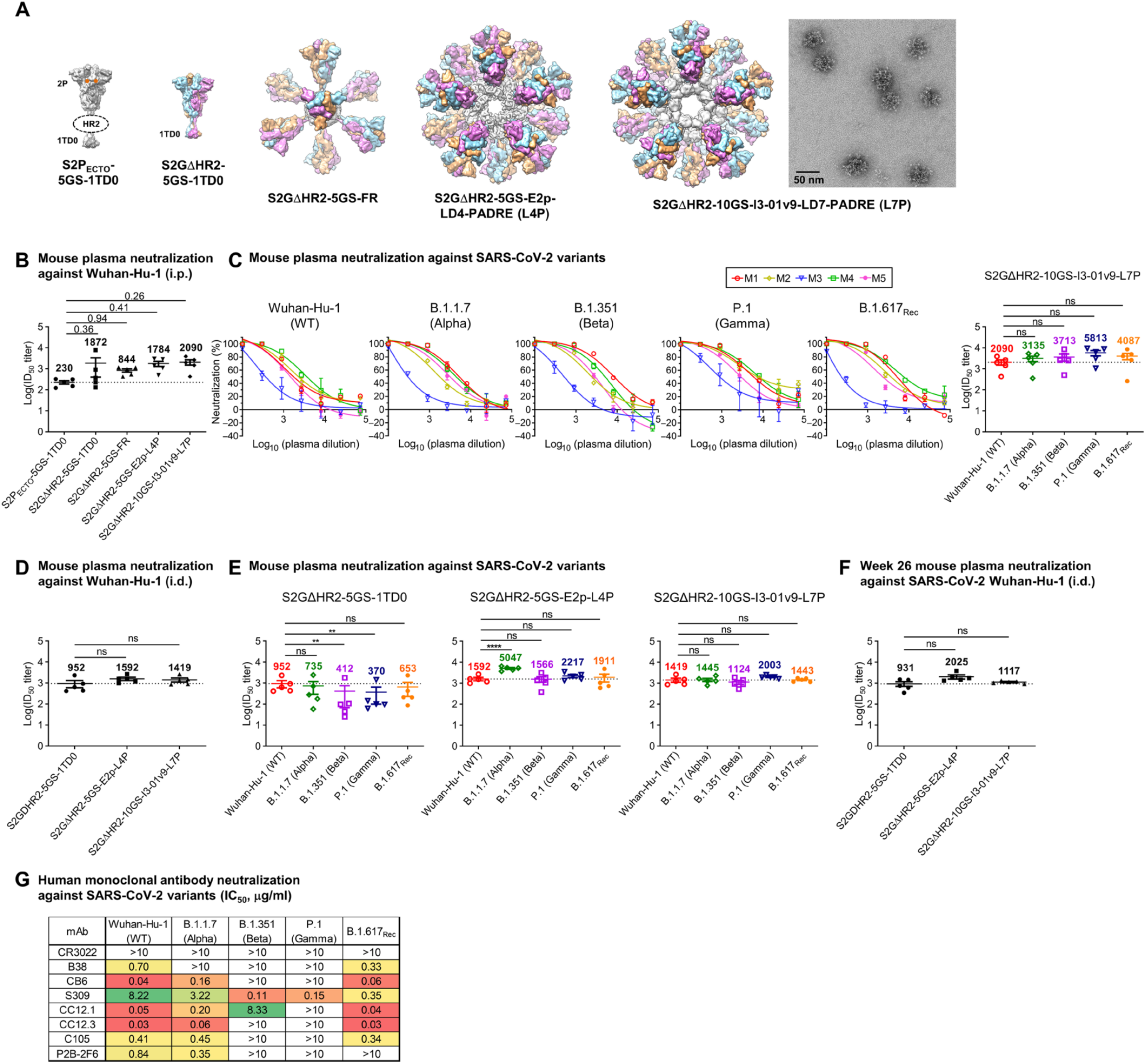
SApNP vaccines induce broadly neutralizing plasma responses to four representative SARS-CoV-2 variants. (**A**) Molecular surface representations of two spike (S2P_ECTO_-5GS-1TD0 and S2GΔHR2-5GS-1TD0) and three spike-SApNP [S2GΔHR2-5GS-FR, S2GΔHR2-5GS-E2p-LD4-PADRE (E2p-L4P), and S2GΔHR2-10GS-I3-01v9-LD7-PADRE (I3-01v9-L7P)] vaccines. Representative EM image of S2GΔHR2-10GS-I3-01v9-L7P SApNPs is shown on the right. (**B**) Neutralization of the wild-type (WT) Wuhan-Hu-1 strain by mouse plasma induced by five different vaccines at week 5 after two intraperitoneal (i.p.) injections (*n* = 5 mice per group). ID_50_ titers derived from SARS-CoV-2-pp neutralization assays are plotted, with average ID_50_ values labeled on the plots. (**C**) Mouse plasma neutralization against Wuhan-Hu-1 and the B.1.1.7, B.1.351, P.1, and B.1.617_Rec_ variants at week 5 after two intraperitoneal injections of the adjuvanted S2GΔHR2-10GS-I3-01v9-L7P vaccine (left panels: percent neutralization plots; right panel: ID_50_ plot). In (B) and (C), the plasma samples were generated in our previous study (*41*) in which mice were immunized with 50 μg of adjuvanted vaccine antigen. (**D**) Neutralization of mouse plasma against the wild-type Wuhan-Hu-1 strain induced by the S2GΔHR2 spike and two large SApNPs at week 5. Vaccines were administered via intradermal (i.d.) footpad injections (0.8 μg per injection, for a total of 3.3 μg per mouse). (**E**) Mouse plasma neutralization against Wuhan-Hu-1 strain and the B.1.1.7, B.1.351, P.1, and B.1.617_Rec_ variants at week 5 after two intradermal footpad injections. (**F**) Neutralization of mouse plasma against Wuhan-Hu-1 induced by the S2GΔHR2 spike and two large SApNPs at week 26. In (B) to (F), the ID_50_ values are plotted as means ± SEM. The data were analyzed using one-way analysis of variance (ANOVA) for comparison between different vaccine groups or repeated measures ANOVA for comparison of ID_50_ titers from the same plasma sample against different SARS-Cov-2 strains. Dunnett’s multiple comparison post hoc test was performed. ns, not significant. **P < 0.01 and ****P < 0.0001. (**G**) Neutralization of five SARS-CoV-2 strains by eight human mAbs. The IC_50_ values were calculated with the % neutralization range constrained within 0.0 to 100.0% and color-coded (white, IC_50_ > 10 μg/ml; green to red, low to high).

We previously demonstrated that the rationally designed S2GΔHR2 spike was more immunogenic than the spike ectodomain with a 2P mutation in the S2 subunit, S2P_ECTO_ (*42*), and SApNPs displaying 8 to 20 spikes outperformed soluble spikes in NAb elicitation (Fig. 1A) (*41*). Notably, the I3-01v9 SApNP that presents 20 S2GΔHR2 spikes induced a potent NAb response to both SARS-CoV-1 and SARS-CoV-2, as well as critically needed T cell responses (*41*). Because SARS-CoV-1 shares only modest sequence similarity (~73% in the RBD) with SARS-CoV-2, we hypothesized that our vaccines would protect against emerging variants, which are much more closely related to the ancestral SARS-CoV-2 strain, Wuhan-Hu-1.

We first assessed the neutralizing activity of polyclonal plasma induced by various spike and SApNP vaccine formulations from our previous study (*41*) against the wild-type SARS-CoV-2 strain, Wuhan-Hu-1, as a baseline for comparison (Fig. 1B). Mouse plasma collected at week 5 after two intraperitoneal injections of adjuvanted vaccine antigens (50 μg) was analyzed in pseudoparticle (pp) neutralization assays (*43*). The soluble S2P_ECTO_ spike elicited the lowest 50% inhibitory dilution (ID_50_) titers, whereas the soluble S2GΔHR2 spike increased neutralization with a 7.1-fold higher average ID_50_ titer, which did not reach statistical significance because of within-group variation. All three spike-presenting SApNPs elicited superior neutralizing responses than the soluble S2P_ECTO_ spike (*41*). Notably, the I3-01v9 SApNP achieved the highest potency, with an average ID_50_ titer of 2090, which was 8.1-fold higher than the soluble S2P_ECTO_ spike. Despite differences in ID_50_ titers, the overall pattern remained the same as reported in our previous study (*41*). The differences might be attributable to the inherent variation of pseudovirus assays (*43*, *44*). We then assessed plasma neutralization against four major SARS-CoV-2 variants (Fig. 1C and fig. S1, A and B). The I3-01v9 SApNP induced a stronger neutralizing response against variants, with 0.5-fold (B.1.1.7), 0.8-fold (B.1.351), 1.8-fold (P.1), and 1.0-fold (B.1.617_Rec_) higher (or 1.5 to 2.8 times) ID_50_ titers compared with the wild-type strain (Fig. 1C). Together, these results confirmed our hypothesis and highlighted the advantages of spike-presenting SApNPs.

Next, we examined the influence of injection dose and route on the plasma neutralizing response to various SARS-CoV-2 strains. To this end, we performed a mouse study in which three groups of mice were immunized with 5, 15, and 45 μg of the I3-01v9 SApNP three times via intraperitoneal injection. All four variants were neutralized by mouse plasma with comparable ID_50_ titers observed across dose groups (fig. S1, C and D). To examine whether routes of injection affect the plasma neutralizing response against variants, we performed another mouse study in which a low dose (3.3 μg) of adjuvanted antigen was intradermally administered into four footpads (i.e., 0.8 μg per footpad). At week 5, the large E2p and I3-01v9 SApNPs that present 20 S2GΔHR2 spikes (~55 to 60 nm) yielded higher ID_50_ titers against the wild-type strain than the soluble S2GΔHR2 spike (Fig. 1D and fig. S1, E and F), whereas a notable reduction of ID_50_ titers against the variants was noted for mouse plasma from the S2GΔHR2 group (Fig. 1E and fig. S1, E and F), suggesting that multivalent display is critical for eliciting a broad neutralizing response. Overall, the E2p and I3-01v9 SApNP groups exhibited similar or slightly stronger plasma neutralization against the four variants relative to the wild-type strain, Wuhan-Hu-1 (Fig. 1E). Last, we assessed longevity of the low-dose vaccination-induced neutralizing response by testing week-26 plasma against Wuhan-Hu-1 (Fig. 1F and fig. S1, G and H). It is noteworthy that ID_50_ titers at week 26 were at the same level as week 5, suggesting a long-lasting protective humoral immunity. In our previous study, a panel of human NAbs was used to evaluate antigenicity of the stabilized S2GΔHR2 spike and SApNPs and validate the SARS-CoV-2-pp neutralization assays (*41*). Here, this antibody panel was tested against SARS-CoV-2-pps that carry spikes of the wild-type strain and the four variants (Fig. 1G and fig. S1I). Lower potency against the B.1.351 and P.1 variants, measured by the 50% inhibitory concentration (IC_50_), was observed for all human NAbs, with the exception of NAb S309, which was identified from a SARS-CoV-1 patient (*45*). This finding is consistent with recent reports on convalescent patient plasma (*26*–*28*). Most human NAbs remained effective against B.1.617_Rec_, showing a similar pattern to the wild-type Wuhan-Hu-1 strain and B.1.1.7 variant, consistent with the results of a recent cohort analysis of convalescent sera from individuals infected with early VOCs against a rising B.1.617 (*33*). As a negative control, mouse plasma induced by the S2GΔHR2-presenting I3-01v9 SApNP was tested against pseudoviruses carrying the murine leukemia virus (MLV) envelope glycoprotein (Env) or MLV-pps. Nonspecific MLV-pp neutralization was not detected for plasma samples produced in two independent immunization experiments (fig. S1, J and K).

Together, our results demonstrate that spike-presenting SApNPs are more advantageous than soluble spikes in eliciting a strong neutralizing response to diverse SARS-CoV-2 variants. In our previous study, soluble SARS-CoV-2 spikes induced a more effective neutralizing response to SARS-CoV-1 than a scaffolded SARS-CoV-2 RBD trimer (*41*). Recently, a two-component RBD-NP vaccine showed reduced serum neutralization of variants bearing the E484K mutation (*46*). It is plausible that both the nanoparticle (NP) platform (one-component SApNP versus two-component NP) and the antigen type (spike versus RBD) contribute to neutralization breadth.

### Adjuvant effect on vaccine-induced NAb and T cell responses

Innate immunity plays an important role in regulating adaptive immunity, including humoral and cellular immune responses (*47*–*49*). Adjuvant-formulated vaccines have been shown to recruit and activate innate immune cells more effectively at injection sites and local lymph nodes (*50*–*52*). Among commonly used adjuvants, AH and AP create depots for the recruitment and activation of antigen-presenting cells at injection sites and sentinel lymph nodes (*53*, *54*), whereas oil-in-water emulsions (e.g., MF59) promote antigen retention and stimulation of antigen-presenting cells in lymph nodes (*55*). Pattern recognition receptor (PRR) agonists (e.g., STING, TLR3, TLR4, TLR7/8, and TLR9 agonists) stimulate antigen-presenting cells at injection sites and nearby lymph nodes (*47*, *52*, *56*–*59*). Macrophage inhibitors (e.g., clodronate liposomes, termed CLs) directly stimulate B cells or inhibit antigen sequestration by subcapsular sinus macrophages, thus resulting in more effective GC stimulation in lymph nodes (*60*). Adjuvant combinations may generate a synergistic immune response by simultaneously activating multiple pathways (*52*, *57*).

**Fig. 2.**
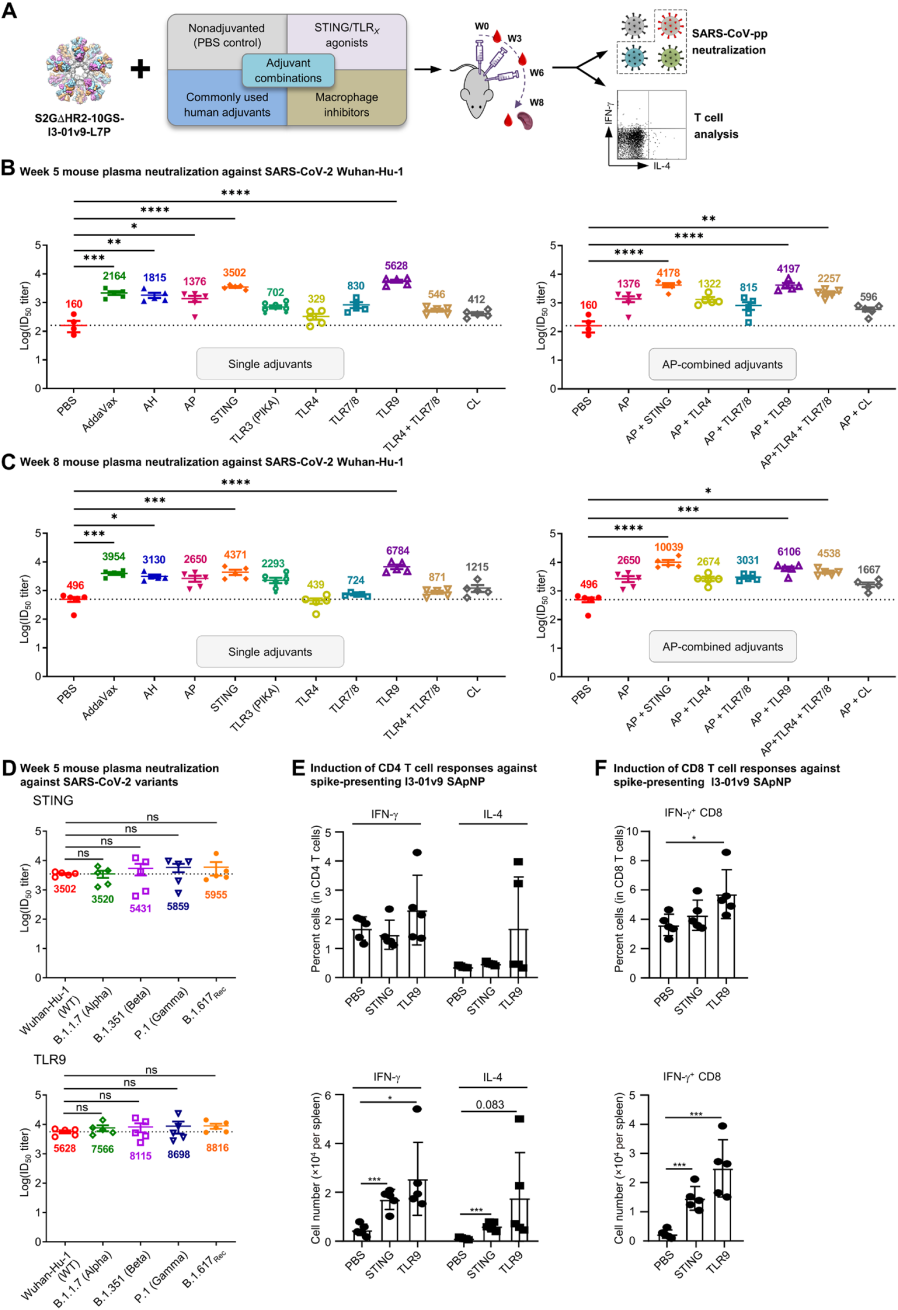
Adjuvants enhance the I3-01v9 SApNP vaccine-induced plasma neuralization of both the wild-type strain and four variants. (**A**) Schematic representation of mouse immunization with the I3-01v9 SApNP with diverse adjuvant formulations and functional assessment by SARS-CoV-2-pp neutralization assays and T cell analysis. Conventional adjuvants, STING/TLR agonists, macrophage inhibitors, and adjuvant combinations were compared to nonadjuvanted control [phosphate-buffered saline (PBS)]. (**B** and **C**) Mouse plasma neutralization against the wild-type SARS-CoV-2 strain, Wuhan-Hu-1, at weeks 5 and 8 after two and three footpad injections, respectively. ID_50_ titers derived from SARS-CoV-2-pp neutralization assays are plotted, with average ID_50_ values labeled on the plots. (**D**) Neutralization against four variants by mouse plasma from STING (top)– and CpG (bottom)–formulated vaccine groups. ID_50_ titers derived from SARS-CoV-2-pp neutralization assays are plotted. Neutralization data were analyzed using either one-way ANOVA (B and C) or repeated measures one-way ANOVA (D) to compare ID_50_ titers. Dunnett’s multiple comparison post hoc test was performed. Splenic mononuclear cells derived from mice in the STING and CpG groups (*n* = 5 mice per group) at week 8 were cultured in the presence of BALB/C DCs pulsed with I3-01v9 SApNP (1 × 10^−7^ mM). Cells were harvested 16 hours following reactivation. (**E**) Production of IFN-γ–producing T_H_1 CD4^+^ T cells and IL-4–producing T_H_2 CD4^+^ T cells. (**F**) IFN-γ–producing CD8^+^ effector T cells. T cell responses were analyzed using one-way ANOVA followed by Tukey’s multiple comparison post hoc test. *P < 0.05, **P < 0.01, ***P < 0.001, and ****P < 0.0001.

To examine the effect of innate signaling pathways on SApNP-induced immune responses, we tested 16 adjuvant formulations in a systematic study (Fig. 2A), in which mice were immunized with the adjuvanted I3-01v9 SApNP (20 μg) via intradermal injections in four footpads (i.e., 5 μg per footpad). We first tested mouse plasma neutralization against the wild-type Wuhan-Hu-1 strain. Mouse plasma at week 2 after a single dose was analyzed in SARS-CoV-2-pp assays, with most groups showing negligible or borderline ID_50_ titers (fig. S2, A and B). We then analyzed mouse plasma at week 5 after two injections (Fig. 2B and fig. S2, C and D). The nonadjuvanted group showed detectable neutralization after two doses, with an average ID_50_ titer of 160, which was used as a baseline in this analysis. By comparison, conventional adjuvants, such as AH, AP, and AddaVax, increased ID_50_ titers by 10.3-, 7.6-, and 12.5-fold, respectively. The macrophage inhibitor CL boosted plasma neutralization by merely 1.6-fold relative to the nonadjuvanted group. Adjuvants that target various PRRs exhibited differential effects on plasma neutralization, increasing ID_50_ titers by 1.1- to 34.2-fold. Notably, STING and unmethylated deoxycytidine-deoxyguanosine (CpG) (TLR9) substantially enhanced neutralizing titers, whereas TLR3, TLR4, and TLR7/8 agonists only exerted a modest effect. In most cases, adjuvants combined with AP further boosted plasma neutralizing activity. For example, when TLR4 and TLR7/8 agonists were mixed with AP, a 3.1-fold increase in ID_50_ titers was observed, suggesting a synergistic effect of stimulating multiple immune pathways. Overall, STING and CpG, either alone or combined with AP, showed plasma neutralization superior to that of any other adjuvant or adjuvant mix, increasing ID_50_ titers by 21- to 34-fold compared with the nonadjuvanted group. This is consistent with the results of the S-Trimer (SCB-2019), which, when formulated with CpG 1018 (TLR9 agonist) and alum adjuvants, induced potent NAb responses in nonhuman primates and human trials (*61*, *62*). Mouse plasma at week 8 showed further increases in ID_50_ titers (1- to 3-fold) for most adjuvant groups (Fig. 2C and fig. S2, E and F). Last, we examined mouse plasma at week 5 from the STING and CpG groups against the B.1.1.7, B.1.351, P.1, and B.1.617_Rec_ variants (Fig. 2D and fig. S2, G and H). Both adjuvant groups exhibited potent neutralizing responses to the four variants, with ID_50_ titers comparable to the wild-type strain.

We previously demonstrated that the AP-formulated I3-01v9 SApNP induces interferon-γ (IFN-γ)–producing CD4^+^ T_H_1 cells and IFN-γ/interleukin-4 (IL-4) double-positive memory CD4^+^ T cells (*41*). Given the superior plasma neutralizing response observed for STING and CpG, we examined the impact of these two adjuvants on vaccine-induced T cell responses. IFN-γ–producing CD4^+^ Th cells are important for optimal antibody responses and the induction of cellular immunity to clear viruses (*63*–*65*). To assess the effect of STING and CpG on vaccine-induced Th cells, we isolated splenocytes from mice 8 weeks after vaccination and cultured them in the presence of BALB/c mouse DCs pulsed with the spike-presenting I3-01v9 SApNP. Compared with the nonadjuvanted control, STING and CpG (TLR9) induced 3.7- and 5.5-fold more IFN-γ–producing CD4^+^ T_H_1 cells and 5.5- and 16-fold more IL-4–producing CD4^+^ T_H_2 cells, respectively (Fig. 2E and fig. S2I). A visible but nonsignificant trend toward a higher frequency of both T_H_1 and T_H_2 cells was noted in mice immunized with the CpG-formulated I3-01v9 SApNP than other formulations. Nonetheless, both adjuvants induced more IFN-γ–producing CD4^+^ T_H_1 cells than IL-4–producing CD4^+^ T_H_2 cells, suggesting a dominant T_H_1 response in these mice. This is consistent with the results for the S-Trimer (SCB-2019), which, when formulated with the AS03 adjuvant or mixed CpG 1018/alum adjuvants, induced T_H_1-biased cellular responses in mice (*61*). STING and CpG also enhanced CD8^+^ T cell responses by 6- and 10-fold, respectively, compared with the phosphate-buffered saline (PBS) control. Notably, this effect was more visible for CpG in terms of both the frequency and number of IFN-γ–producing CD8^+^ effector T cells (Fig. 2F and fig. S2J).

Our results demonstrate that the I3-01v9 SApNP itself is immunogenic, and adjuvants can further enhance vaccine-induced NAb responses in plasma by up to 35-fold. The I3-01v9 SApNP, when formulated with the STING or TLR9 agonist, yielded the highest ID_50_ titers with robust CD4^+^ and CD8^+^ T cell responses, highlighting their potential as adjuvants in the development of more effective SARS-CoV-2 vaccines.

### Diverse variant-neutralizing mouse antibody lineages identified by single-cell analysis

Although plasma neutralization confirmed the effectiveness of our newly designed SARS-CoV-2 vaccines (*41*) against variants, the nature of this response was unclear. It might result from multiple NAb lineages that each target a specific strain (nonoverlapping), a few bNAb lineages that are each able to block multiple strains (overlapping), or a combination of both. Previously, we used antigen-specific single-cell sorting to identify potent mouse NAbs elicited by an I3-01 SApNP that presents 20 stabilized HIV-1 Env trimers (*66*). Here, we applied a similar strategy to decipher NAb responses induced by SARS-CoV-2 vaccines using mouse samples from our previous study (*41*), for which potent plasma neutralization against four variants has been verified (Fig. 1C).

**Fig. 3.**
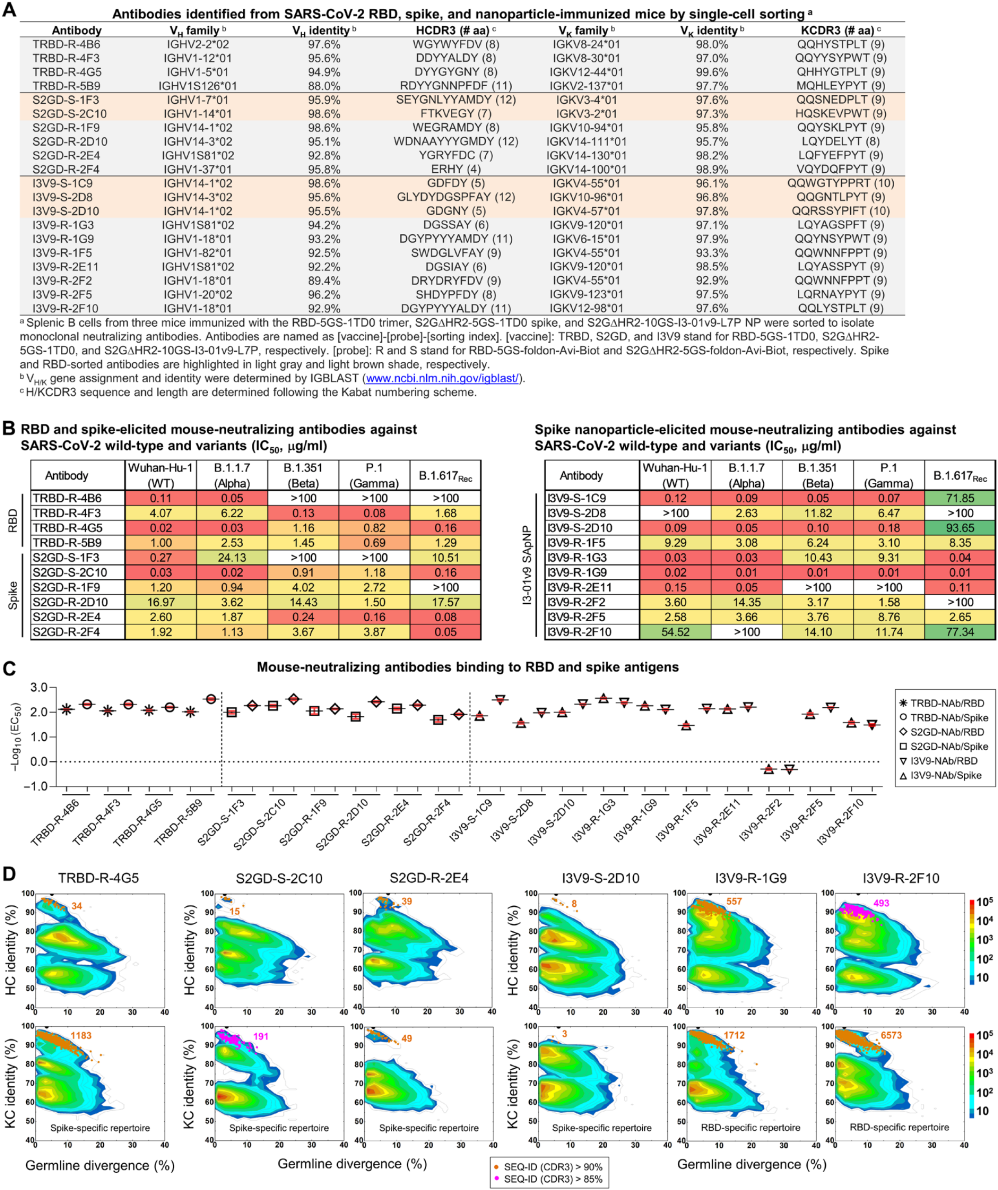
Single-cell isolation identifies vaccine-elicited mouse NAb lineages with diverse breadth and potency. (**A**) Genetic analysis of 20 mouse antibodies identified from M2 in the RBD-5GS-1TD0 trimer group (4), M4 in the S2GΔHR2-5GS-1TD0 spike group (6), and M2 in the S2GΔHR2-10GS-I3-01v9-L7P SApNP group (10). Antibodies isolated by the RBD and spike probes are highlighted in light gray and orange shade, respectively. aa, amino acid. (**B**) Neutralization of five SARS-CoV-2 strains by 10 RBD and spike-elicited mouse antibodies (left) and 10 SApNP-elicited mouse antibodies (right). The IC_50_ values were calculated with the % neutralization range constrained within 0.0 to 100.0% and color-coded (white, IC_50_ > 100 μg/ml; green to red, low to high). (**C**) EC_50_ (μg/ml) values of 20 mouse antibodies binding to the two SARS-CoV-2 antigens, the RBD monomer and S2GΔHR2-5GS-1TD0 spike, both with the Wuhuan-Hu-1 backbone. Antigen binding was measured by ELISA in duplicate, with mean value and SD shown as black and red lines, respectively. (**D**) Divergence-identity analysis of selected mouse NAbs in the context of RBD/spike-specific splenic B cells. HCs and KCs are plotted as a function of sequence identity to the template and sequence divergence from putative germline genes. Color coding denotes sequence density. The template and sequences identified based on the V gene assignment and a CDR3 identity of 90%/85% or greater to the template are shown as black and orange/magenta dots on the 2D plots, with the number of related sequences labeled accordingly. The 2D plots for other NAbs are shown in fig. S4 (D to F).

Spleen samples from Mouse 4 (M4) in the spike group (S2GΔHR2-5GS-1TD0) and M2 in the spike-SApNP group (S2GΔHR2-10GS-I3-01v9-L7P), along with a control sample from M2 in the RBD (RBD-5GS-1TD0) group were analyzed. Two probes, RBD-5GS-foldon-Avi and S2GΔHR2-5GS-foldon-Avi, were produced, biotinylated, and purified to facilitate antigen-specific B cell sorting (fig. S3, A and B). Following antibody cloning, reconstituted mouse mAbs were tested for neutralizing activity against the wild-type strain, Wuhan-Hu-1, in SARS-CoV-2-pp assays. A total of 20 mAbs, 4 from the RBD group (fig. S3C), 6 from the spike group (fig. S3D), and 10 from the I3-01v9 SApNP group (fig. S3E), were found to be NAbs. The genetic analysis of mAb sequences revealed some salient features of the vaccine-induced NAb response in mice (Fig. 3A). Overall, these mAbs evolved from diverse germline origins. The RBD-elicited mAbs appeared to use distinct germline variable (V) genes for both heavy chain (HC) and κ-light chain (KC), or V_H_ and V_K_, respectively, whereas the spike and I301v9 SApNP-elicited mAbs shared some common V_H_ genes, such as IGHV14-1/3 and IGHV1S81. This result was not unexpected because the RBD vaccine presents a structurally distinct antigen to the immune system compared with the spike and I3-01v9 SApNP vaccines, which both contain the S2GΔHR2 spike. These mAbs showed low levels of somatic hypermutation (SHM) with respect to their germline genes. HC complementarity-determining region (HCDR3) loops ranged from 4 to 12 amino acids in length, whereas most KCs contained 9–amino acid KCDR3 loops. Collectively, diverse germline genes and HCDR3 loops, accompanied by low degrees of SHM, suggest that many antibody lineages must have been generated upon vaccination, and some could achieve neutralizing activity without an extensive maturation process.

We then examined the biological function of these mouse mAbs. Neutralizing activity was assessed in SARS-CoV-2-pp assays against the wild-type strain and four variants (Fig. 3B and fig. S3F). Overall, diverse yet consistent patterns were observed for the three sets of mAbs. Both the RBD vaccine (an RBD scaffold) and the two spike vaccines, albeit in different forms, appeared to elicit potent NAbs against the wild-type strain. MAbs TRBD-R-4G5, S2GD-S-2C10, and I3V9-R-1G9 showed similar IC_50_ values (0.02 to 0.03 μg/ml) against Wuhan-Hu-1, on par with the human NAbs CB6 (*67*) and CC12.1/3 (*68*) (Fig. 1F). All three vaccines elicited bNAb responses, despite variation in potency for different mAbs against different strains. Notably, I3V9-R-1G9, which was isolated from an I3-01v9 SApNP-immunized mouse, demonstrated high potency across all four variants (IC_50_, 0.01 to 0.02 μg/ml). This bNAb provided evidence that individual bNAb lineages may critically contribute to the plasma neutralization of diverse variants (Fig. 1C). All three vaccines generated NAbs that preferentially neutralize specific SARS-CoV-2 strains. For example, TRBD-R-4B6 was more effective against the wild-type strain and an early VOC, B.1.1.7, whereas S2GD-R-2E4 neutralized B.1.351, P.1, and B.1.617_Rec_ with greater potency. Notably, more than 60% (13) of the mAbs exhibited different patterns in the neutralization of B.1.617_Rec_ versus VOCs B.1.351 and P.1, as indicated by the fold change in IC_50_, suggesting that B.1.617 may represent a distinct SARS-CoV-2 lineage. Although RBD-isolated NAbs likely neutralized SARS-CoV-2 by blocking its receptor binding, those spike-isolated NAbs could target the RBD, NTD, or epitopes in the S2 subunit. Thus, we tested these mAbs in an enzyme-linked immunosorbent assay (ELISA) against the RBD monomer and S2GΔHR2-5GS-1TD0 spike, both on the basis of the wild-type Wuhan-Hu-1 backbone (Fig. 3C and fig. S3, G and H). Overall, all of the NAbs bound the RBD and spike with a half-maximal concentration (EC_50_) of 0.03 μg/ml or lower, except for I3V9-R-2F2 (1.97 μg/ml for the RBD and 2.05 μg/ml for the spike). Most (15) NAbs showed greater binding affinity (or lower EC_50_ values) for the spike, suggesting that the two arms of the immunoglobulin (Ig) can each interact with one RBD of the spike, resulting in an avidity effect. Notably, diverse binding patterns were observed for I3-01v9 SApNP-elicited NAbs. Although I3V9-S-1C9 and I3V9-S-1F5 bound to the spike more favorably than the RBD, as indicated by a 4.7- to 4.9-fold reduction of their EC_50_ values, three NAbs from this group (I3V9-R-1G3, I3V9-R-1G9, and I3V9-R-2F10) preferred the RBD monomer over the spike. This preference might be explained by steric hindrance when these NAbs approach the RBDs on a trimeric spike with specific angles.

Last, we characterized these mouse NAbs in antigen-specific B cell repertoires by next-generation sequencing (NGS), as previously demonstrated for NAbs isolated from HIV-1 SApNP-immunized mice and rabbits (*66*). Using the same RBD and spike probes (fig. S3A), ~1500 splenic B cells were bulk-sorted from each of the three mice that were analyzed by single-cell sorting for mAb isolation (fig. S4A). Unbiased mouse antibody HC and KC libraries were constructed and sequenced on an Ion S5 platform, which yielded up to 4 million raw reads (fig. S4B). The antibody NGS data were then processed using a mouse antibodyomics pipeline (*69*) to remove low-quality reads, resulting in 0.11 to 0.41 million full-length HCs and KCs (fig. S4B). Quantitative profiles of critical antibody properties, such as germline gene usage, the degree of SHM, and CDR3 loop length, were determined for the RBD and spike-specific B cell populations (fig. S4C). All 20 single-cell–sorted mouse NAbs could well fall in the range of these repertoire profiles, but some V_H_/V_K_ genes that accounted for large portions of antigen-specific B cells, such as IGHV9 and IGHV5, were not used by any NAbs, suggesting that they might give rise to nonneutralizing binding antibodies. Two-dimensional (2D) divergence/identity plots were generated to visualize these NAbs in the context of NGS-derived B cell repertoires (Fig. 3D and fig. S4, D to F). Somatic variants were identified for each NAb by searching for sequences of the same V_H_/V_K_ gene with a CDR3 identity cutoff of 90% (or 85% for evolutionarily more remote variants). For the most potent NAb, TRBD-R-4G5, from an RBD-immunized mouse (M2), 34 HC variants were identified that overlapped with an “island” of high sequence similarity to TRBD-R-4G5 on the plot, whereas more KC variants (1183) were found, likely due to the lack of diversity in the KCDR3 region. A similar pattern was observed for the potent bNAb, I3V9-R-1G9, from an SApNP-immunized mouse (M2). By comparison, fewer putative somatic variants were identified for other NAbs in the antigen-specific B cell repertoires regardless of the sorting probe used (fig. S4, D to F), suggesting that these NAbs either were from less prevalent lineages or were generated in response to a previous injection (each mouse received four doses) (*41*). Similar observations were reported for the vaccination of nonhuman primates and humans in longitudinal repertoire analyses of single-cell–sorted NAbs (*70*, *71*).

Single-cell isolation identified a panel of mouse mAbs with different neutralization breadth and potency against the wild-type SARS-CoV-2 strain and four major variants. The ELISA analysis suggested that the I3-01v9 SApNP can elicit NAbs with more diverse angles of approach to their epitopes than the RBD and soluble spike vaccines. Structural analysis by crystallography and EM will provide a more detailed understanding of epitope recognition by these mouse mAbs.

### Distribution and trafficking of I3-01v9 SApNPs in mouse lymph nodes

**Fig. 4.**
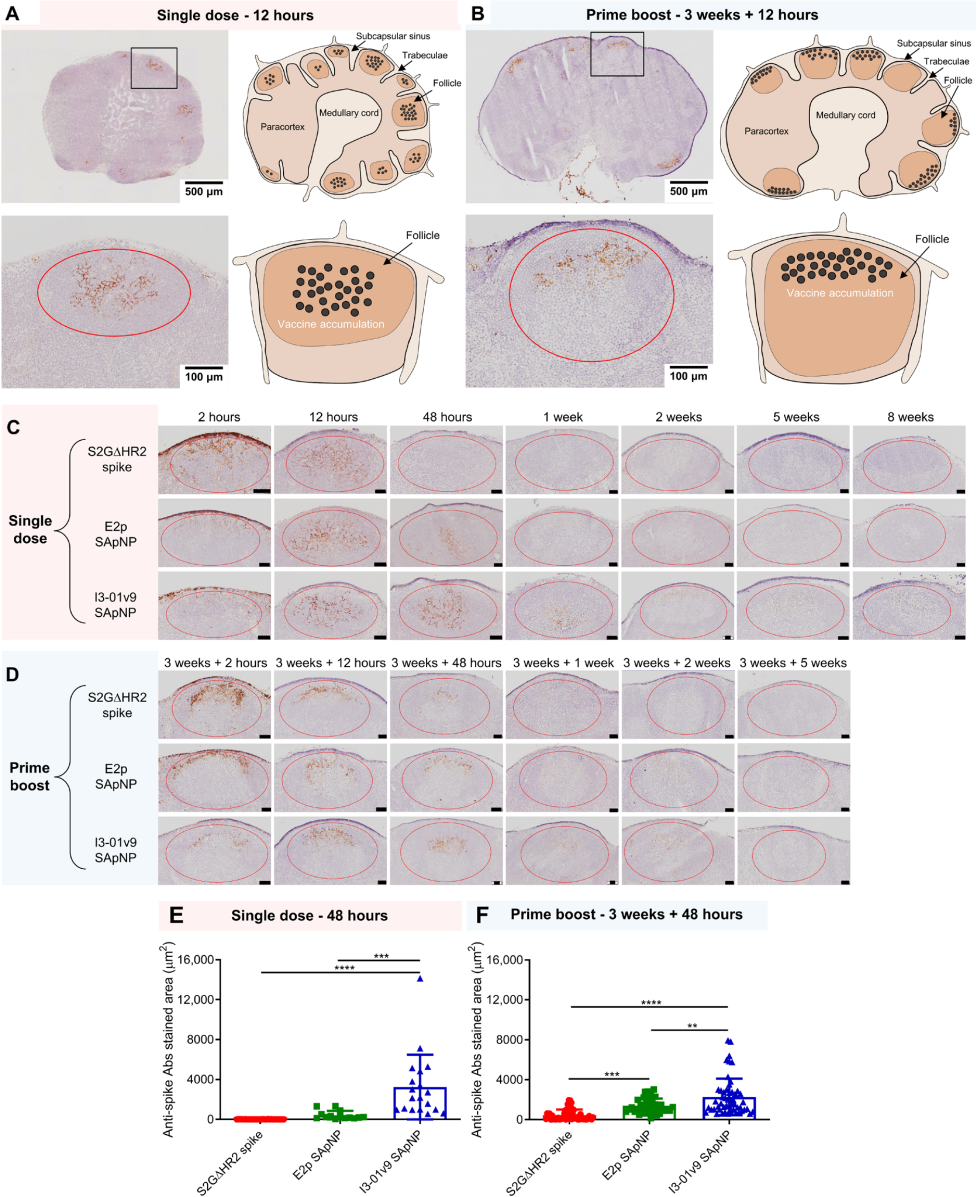
SARS-CoV-2 SApNP vaccines induce long-term lymph node follicle retention. (**A** and **B**) S2GΔHR2-presenting I3-01v9 SApNP vaccine distribution in a lymph node 12 hours after (A) a single-dose or (B) prime-boost footpad injections (10 μg per footpad, 40 μg per mouse). A schematic illustration of SApNPs in lymph node follicles is shown. (**C** and **D**) Histological images of the S2GΔHR2 spike and S2GΔHR2-presenting E2p and I3-01v9 SApNP vaccine trafficking and retention in lymph node follicles 2 hours to 8 weeks after (C) single-dose or (D) prime-boost injections, with a scale bar of 50 μm shown for each image. (**E** and **F**) Quantification of vaccine accumulation in lymph node follicles 48 hours after (E) a single-dose or (F) prime-boost injections. In mouse immunization, soluble spike was mixed with AddaVax, E2p SApNP was mixed with AddaVax, and I3-01v9 SApNP was mixed with AP. Data were collected from more than 10 lymph node follicles (*n* = 3 to 4 mice per group). The data points are expressed as means ± SD. The data were analyzed using one-way ANOVA followed by Tukey’s multiple comparison post hoc test. **P < 0.01, ***P < 0.001, and ****P < 0.0001.

After validating these vaccines against variants at both the plasma and mAb levels, we studied in vivo behaviors of the S2GΔHR2 spike and two large 60-mer SApNPs to understand why SApNPs outperform soluble spikes in bNAb elicitation. In principle, these SApNPs need to be transported to lymph nodes, retained, and presented to various immune cell populations to induce robust innate and adaptive immune responses. Here, we first examined the transport and distribution of I3-01v9 SApNPs in mouse lymph nodes via footpad injections (10 μg per footpad). The mice were euthanized 12 hours after single-dose (Fig. 4A) and prime-boost (Fig. 4B) regimens. The axillary, brachial, and popliteal sentinel lymph nodes were isolated for histological analysis. The lymph node tissues were stained with the human anti-spike antibody P2B-2F6 (*72*) to characterize SARS-CoV-2 spikes presented on the I3-01v9 SApNPs. Consistent with a previous study (*73*), SApNPs accumulated in lymph node follicles, regardless of the number of doses. SApNPs were sequestrated in the center of lymph node follicles after a single dose (Fig. 4A, images on the left, schematics on the right) but were located along the outer layer of expanded lymph node follicles after the second injection due to preexisting humoral immunity (i.e., GC reactions) that was induced by the first dose (Fig. 4B, images on the left, schematics on the right). Overall, most of the SApNPs accumulated in lymph node follicles, but their distribution differed slightly, depending on the doses.

In this context, we examined patterns of trafficking and lymph node follicle retention for soluble S2GΔHR2 spike versus the S2GΔHR2-presenting E2p and I3-01v9 SApNPs. To facilitate this analysis, the mice were euthanized 2 hours to 8 weeks after a single dose (Fig. 4C) and 2 hours to 5 weeks after the boost (Fig. 4D). The antigen dose was normalized to the total amount of protein (40 μg per mouse) that was injected into four footpads (10 μg per footpad). As shown in Fig. 4C, the S2GΔHR2 spikes that trafficked into lymph node follicles at 2 hours cleared within 48 hours. In contrast, the two large SApNPs accumulated in the subcapsular sinus at 2 hours and then trafficked into follicles 12 hours after the single-dose injection. Notably, I3-01v9 SApNPs remained detectable in lymph node follicles after 2 weeks, suggesting sixfold longer retention than the S2GΔHR2 spike (Fig. 4C). The results for these protein NPs are thus consistent with the pattern of size dependency that was observed for ovalbumin-conjugated gold NPs in a previous study (*73*), in which small (5 to 15 nm) NPs cleared shortly after the injection, whereas large (50 to 100 nm) NPs were retained in lymph node follicles for weeks. Similar patterns of antigen retention were observed after the second injection, although the boost appeared to exert a more positive effect on the soluble spike, which could be detected in lymph node follicles at 48 hours (Fig. 4D). Nonetheless, prolonged retention was observed for both E2p and I3-01v9 SApNPs 2 weeks after the boost injection. Overall, the multivalent display of S2GΔHR2 spikes on the I3-01v9 SApNP resulted in 325- and 4-fold greater accumulation in lymph node follicles compared with the soluble spike 48 hours after the single-dose (Fig. 4E) and prime-boost (Fig. 4F) injections, respectively. These findings reveal the advantage of a leading vaccine candidate identified in our previous study, S2GΔHR2-10GS-I3-01v9-L7P (*41*), in terms of antigen retention in lymph node follicles.

### Retention and presentation of I3-01v9 SApNPs on FDC dendrites

**Fig. 5.**
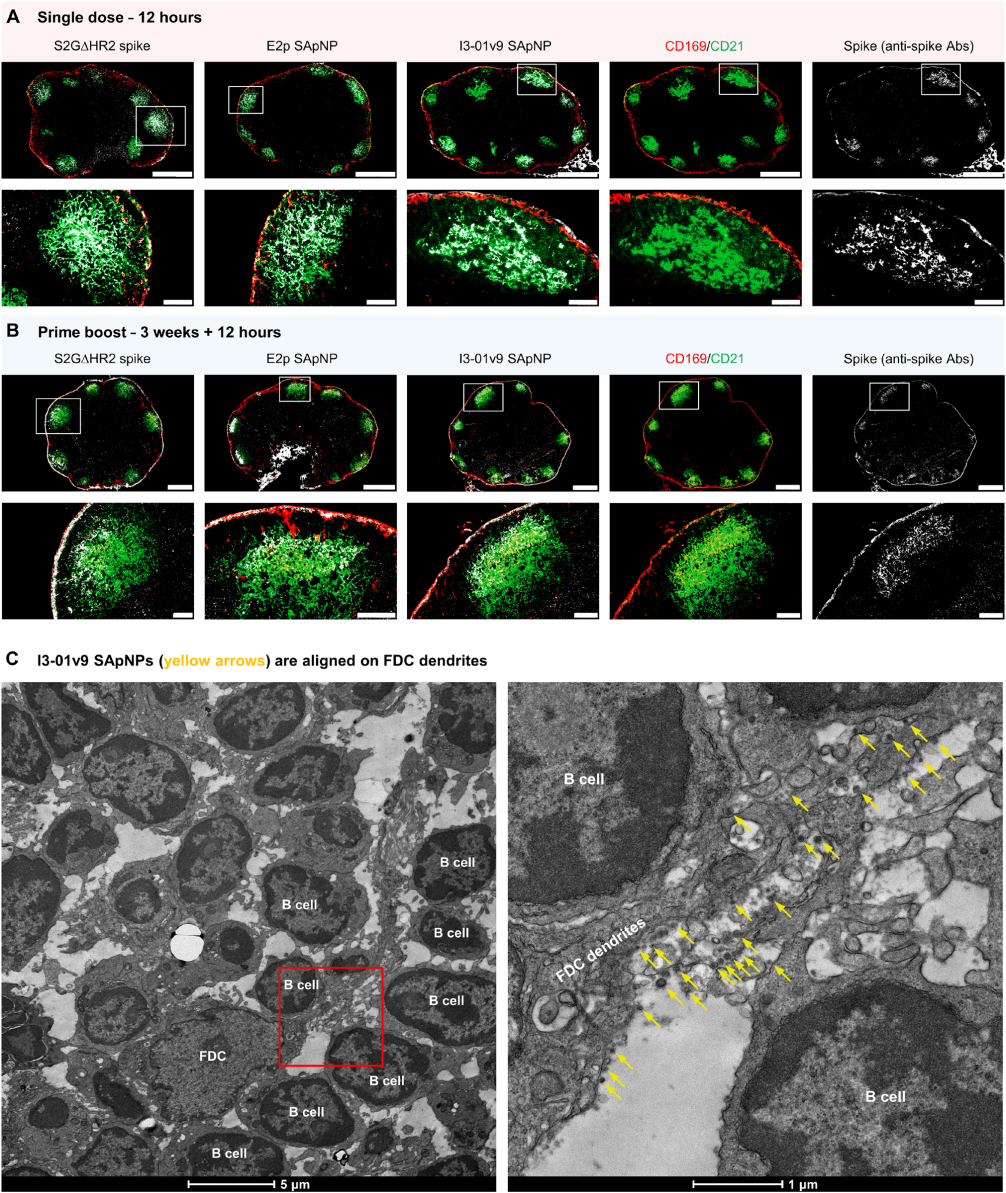
SARS-CoV-2 SApNP vaccines interact with FDCs and are presented on FDC dendrites to B cells. (**A** and **B**) S2GΔHR2 spike and S2GΔHR2-presenting E2p and I3-01v9 SApNP vaccine interaction with FDC networks in lymph node follicles 12 hours after (A) a single-dose or (B) prime-boost injections (10 μg per footpad, 40 μg per mouse). Vaccine antigens (the S2GΔHR2 spike and S2GΔHR2-presenting E2p and I3-01v9 SApNPs) colocalized with FDC networks. Immunostaining is color-coded (green, CD21; red, CD169; white, anti-spike), with scale bars of 500 and 100 μm shown for a complete lymph node and an enlarged image of a follicle, respectively. (**C**) Representative TEM images of an FDC surrounded by multiple B cells. S2GΔHR2-presenting I3-01v9 SApNPs (yellow arrows) presented on FDC dendrites.

Antigen retention and presentation in lymph node follicles are prerequisites to the stimulation of robust B cell responses and GC reactions (*34*, *36*). Resident cells spatially rearrange antigens and present them to B cells. FDCs are resident stromal cells in follicles and retain soluble antigens, immune complexes, virus-like particles (VLPs), viruses, and bacteria (*73*–*76*). FDCs are also key to GC initiation, maintenance, and B cell affinity maturation (*37*, *77*, *78*). Here, we hypothesized that FDCs comprise the major cell population in lymph node follicles that retain SARS-CoV-2 spikes and spike-presenting SApNPs. To test this hypothesis, we administered vaccines via footpad injections and collected mouse lymph nodes at the peak of accumulation (12 hours) after single-dose (Fig. 5A) and prime-boost (Fig. 5B) injections. Lymph node tissues were stained with the anti-spike antibody P2B-2F6 (*72*) for the S2GΔHR2 spike, as well as anti-CD21 and anti-CD169 antibodies for FDCs and subcapsular sinus macrophages, respectively. The spike and SApNP (E2p or I3-01v9) signals colocalized with FDC (CD21^+^) networks in lymph node follicles (Fig. 5, A and B). This result confirmed the critical role of FDC networks in mediating vaccine retention in lymph node follicles.

The induction of potent bNAb responses by spike-presenting SApNPs in mice suggests the effective activation of naïve B cells and subsequent recalls by cross-linking B cell receptors (*76*, *79*, *80*). We visualized the interface between FDC networks and B cells to better understand how FDC networks present SApNPs to engage B cells. Briefly, fresh lymph nodes were isolated and directly immersed in fixative. The processed tissue samples were sectioned and stained on copper grids for TEM analysis. We first determined whether SApNPs, such as the S2GΔHR2-presenting I3-01v9 SApNP, remain intact in vivo (fig. S5). Mouse lymph nodes were isolated 2 hours after the injection of a high dose (50 μg) of the nonadjuvanted I3-01v9 SApNP. The TEM images revealed that round-shape granules corresponding to intact SApNP aligned on the macrophage surface or inside endolysosomes of the macrophage in a lymph node (fig. S5). We next studied the relative location between FDCs and I3-01v9 SApNPs and how FDCs present SApNPs to B cells. Mouse lymph nodes were collected 2, 12, and 48 hours after a single dose (50 μg) and 12 hours after the boost of the I3-01v9 SApNP vaccine. The FDCs exhibited the characteristic morphology of long dendrites that surrounded and interacted with B cells in lymph node follicles (Fig. 5C and fig. S6). Few I3-01v9 SApNPs were observed on FDC dendrites at 2 hours (fig. S6D), whereas, notably, more SApNPs migrated to and aligned on FDC dendrites at 12 and 48 hours (Fig. 5C and fig. S6, A to C, yellow arrows). The TEM images indicated that FDCs can present many SApNPs to neighboring B cells in this “hugging mode” in which their long dendrites brace B cells to maximize interactions between multivalently displayed spikes and B cell receptors. These results demonstrated the intrinsic nature of FDCs as a reservoir for the sequestration, retention, and presentation of VLPs, or SApNPs with similar molecular traits, to initiate GC reactions.

### Robust GC reactions induced by spike-presenting SApNPs

**Fig. 6.**
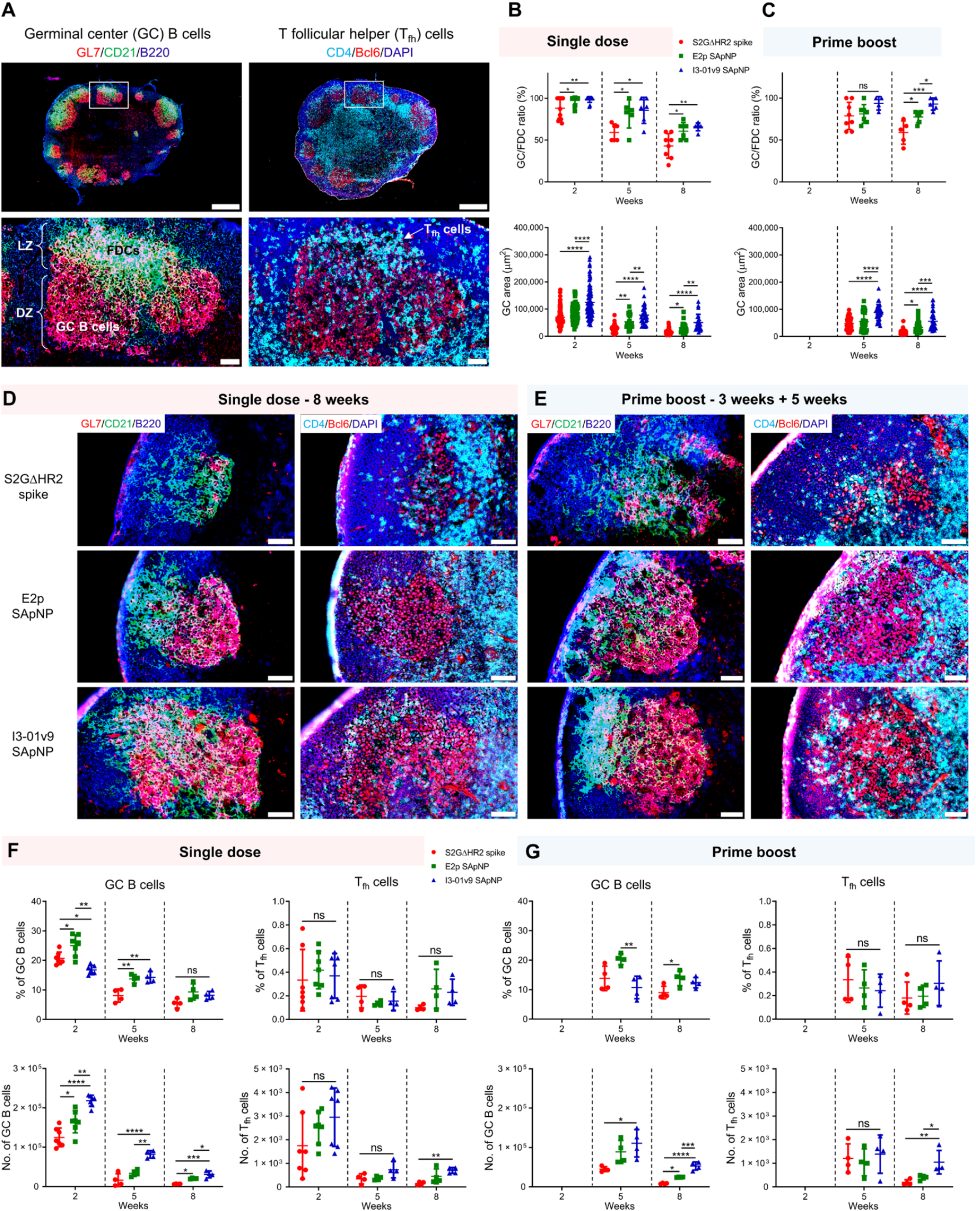
SARS-CoV-2 SApNP vaccines induce robust long-lived GCs. (**A**) Top: Representative immunohistological images of GCs at week 2 after a single-dose injection of the S2GΔHR2-presenting I3-01v9 SApNP vaccine (10 μg per injection, 40 μg per mouse). Bottom: GC B cells (GL7^+^, red) adjacent to FDCs (CD21^+^, green) in lymph node follicles (left) and T_fh_ cells in the light zone (LZ) of GCs (right). Scale bars of 500 and 50 μm are shown for a complete lymph node and an enlarged image of a follicle, respectively. DZ, dark zone. (**B** and **C**) Quantification of GC reactions using immunofluorescent images: GC/FDC ratio and sizes of GCs 2, 5, and 8 weeks after (B) single-dose or (C) prime-boost injections (*n* = 4 to 7 mice per group). The GC/FDC ratio is defined as whether the GC formation is associated with an FDC network (%). (**D** and **E**) Representative immunohistological images of GCs in mice immunized using S2GΔHR2 spike or S2GΔHR2-presenting E2p and I3-01v9 SApNP vaccines at week 8 after (D) single-dose or (E) prime-boost injections, with a scale bar of 50 μm shown for each image. DAPI, 4′,6-diamidino-2-phenylindole. (**F** and **G**) Quantification of GC reactions using flow cytometry: percentage and number of GC B cells and T_fh_ cells 2, 5, and 8 weeks after (F) single-dose or (G) prime-boost injections. The data points are shown as means ± SD. The data were analyzed using one-way ANOVA followed by Tukey’s multiple comparison post hoc test for each time point. *P < 0.05, **P < 0.01, ***P < 0.001, and ****P < 0.0001.

Long-lived GC reactions induce immune stimulation for B cell selection and affinity maturation, as well as production of immune memory and bNAb responses (*34*, *35*, *40*). Here, we investigated whether the prolonged retention of S2GΔHR2-presenting E2p and I3-01v9 SApNPs induces more robust GCs in lymph node follicles than the soluble S2GΔHR2 spike. Immunohistological analysis was performed to characterize GC B cells (GL7^+^) and T follicular helper (T_fh_) cells (CD4^+^Bcl6^+^). For the I3-01v9 SApNP, 2 weeks after immunization, we observed robust GCs in lymph node B cell follicles (B220^+^) with well-formed dark zone and light zone compartments, which contain GC B cells, FDCs, and T_fh_ cells (Fig. 6A) (*35*, *81*–*83*). We then extended the analysis to the S2GΔHR2 spike and spike-presenting SApNPs 2, 5, and 8 weeks after the single-dose injection (Fig. 6B and fig. S7, A to C) and 2 and 5 weeks after the boost (Fig. 6C and fig. S7, D and E). Two metrics, the GC/FDC ratio [i.e., whether GC formation is associated with an FDC network (%)] and GC size (i.e., occupied area), were used. Overall, the soluble spike and both large SApNPs induced robust GCs 2 weeks after immunization (Fig. 6B and fig. S7A). The E2p and I3-01v9 SApNPs that present 20 spikes induced robust, long-lived GCs, whereas the spike alone failed to sustain robust GCs at week 8 with either the single-dose (Fig. 6, B and D) or prime-boost (Fig. 6, C and E) injections. The I3-01v9 SApNP generated larger GCs than the soluble spike, 2.0-fold larger after the single dose (Fig. 6, B and D) and 2.4-fold larger after the boost (Fig. 6, C and E), measured at week 8.

We further characterized GC reactions by flow cytometry. Fresh mouse lymph nodes were disaggregated into a single cell suspension and stained with an antibody cocktail to quantify GC B cells and T_fh_ cells (fig. S8A). The results were consistent with the immunohistological analysis, in which all spike-based vaccine antigens, including the S2GΔHR2 spike and SApNPs, showed robust GCs at week 2 after the injection that declined over time, as measured at weeks 5 and 8 (Fig. 6F). The E2p and I3-01v9 SApNPs generated a larger population of GC B cells than both the S2P_ECTO_ and S2GΔHR2 spikes at week 2 (fig. S8, B and C). Although the boost dose had little impact on the frequency of GC B cells and T_fh_ cells, it appeared to extend GC formation within lymph nodes (Fig. 6, F and G), which may promote B cell development toward bNAbs. Notably, the GC B cell and T_fh_ cell populations elicited by the soluble S2GΔHR2 spike were barely detectable 5 weeks after immunization (Fig. 6, F and G). This result was reminiscent of a recent study of an mRNA vaccine, in which GC reactions diminished to baseline levels at week 4 after a single-dose injection (*84*). The S2GΔHR2-presenting I3-01v9 SApNP generated 3.7/5.2-fold more GC B cells and 3.7/4.4-fold more T_fh_ cells than the soluble S2GΔHR2 spike at week 8 after one/two-dose immunization (Fig. 6, F and G). Therefore, SApNPs that were retained on FDC dendrites could present NAb epitopes to enable more effective B cell recognition than the soluble spike and consequently induce more robust and long-lived GCs in lymph nodes. Patterns of trafficking and retention may be specific to antigen size, as shown previously (*73*) and in the present study (Figs. 4 and 5), but GC reactions are largely determined by vaccine adjuvants. This effect was briefly demonstrated for the E2p and I3-01v9 SApNPs, which were previously formulated with the AddaVax and AP adjuvants (*41*). At week 2 after a single-dose injection, the adjuvanted SApNPs induced stronger GC reactions than the nonadjuvanted groups (fig. S9). This result can also explain the differences in plasma neutralization between the adjuvanted and nonadjuvanted I3-01v9 SApNPs (Fig. 2).

NGS has been used to assess vaccine-draining lymph node B cell responses (*85*). Here, we characterized lymph node B cells at the repertoire level for three groups of mice immunized with two doses (3.3 μg each) of the S2GΔHR2 spike, E2p, and I301v9 SApNPs via footpad injections. At this dose level, the spike showed less effective plasma neutralization of variants than the large SApNPs (Fig. 1E). Given their differences in retention, presentation, and GC reaction (Figs. 4 to 6), they were expected to yield different lymph node B cell profiles. Indeed, antigen-specific sorting identified more spike-targeting lymph node B cells from the I3-01v9 SApNP group than both the spike and E2p SApNP groups (fig. S10A). The antibody NGS data were processed by the mouse antibodyomics pipeline (fig. S10B) (*69*) to derive quantitative B cell profiles (fig. S10, C to E). Compared with the spike, the I3-01v9 SApNP appeared to activate fewer V_H_/V_K_ genes (fig. S10F, left two) while generating a larger population of spike-specific lymph node B cells (fig. S10A). The three vaccine groups exhibited a similar degree of SHM for V_H_ genes, with the I3-01v9 SApNP showing the highest SHM for V_K_ genes (fig. S10F, middle two). A highly uniform HCDR3 length distribution (~10 amino acids) was observed for mice in the I3-01v9 SApNP group with little variation, as measured by the root mean square fluctuation (fig. S10F, right two). In our previous studies (*86*, *87*), a similar approach was applied to assess hepatitis C virus (HCV) and Ebola virus (EBOV) vaccine-induced B cell responses in the spleen, a major lymphoid organ (*88*), after mice received four intraperitoneal injections. We observed distinct B cell profiles associated with the viral antigen and NP platform (*86*, *87*). Here, the lymph node B cell profiles appeared to be rather different, revealing the complex inner workings of another primary site for vaccine-induced immunity. Notably, I3-01v9 SANP exhibited more “focused” B cell activation and development in vaccine-draining lymph nodes, as indicated by fewer activated germline genes and a narrower HCDR3 length distribution. More in-depth studies are needed to investigate the effect of injection route, adjuvant, and lymphoid organ, in addition to viral antigen and NP platform, on the vaccine-induced B cell repertoires. Single-cell immune profiling and antibody isolation (*89*) may provide further insights into the clonality of vaccine-induced B cell lineages within lymph nodes.

## DISCUSSION

To end the COVID-19 pandemic, vaccines need to effectively block current and emerging SARS-CoV-2 variants that evade NAb responses by mutating key epitopes on the viral spike (*31*). To overcome this challenge, some suggested that COVID-19 vaccines need to be updated on a regular basis (*30*–*32*), whereas others developed mosaic or cocktail vaccines for related sarbecoviruses (*46*, *90*). These vaccine strategies need to be evaluated for long-term protection, because SARS-CoV-2 is evolving rapidly and may acquire new mutations to evade vaccine-induced immunity (e.g., B.1.617) (*11*). In our previous study (*41*), the spike-presenting SApNPs induced a potent NAb response to SARS-CoV-1, which is evolutionarily much more distant to the wild-type SARS-CoV-2 strain, Wuhan-Hu-1, than all its circulating variants. Emerging data from human serum analysis suggested that vaccines derived from early pandemic strains may provide broad protection against current variants (*33*). On the basis of these findings, we hypothesized that SApNPs presenting stabilized ancestral Wuhan-Hu-1 spikes may provide an effective vaccine against SARS-CoV-2 variants. In the present study, we sought to confirm this hypothesis by testing four major variants and, if proven true, investigate the mechanism underlying such a broadly protective vaccine.

We explored several critical aspects related to the vaccine response, with a focus on the lead candidate identified in our previous study, S2GΔHR2-10GS-I3-01v9-L7P (*41*). We first tested vaccine-induced mouse plasma, which represents a polyclonal response, against four SARS-CoV-2 variants. Mouse plasma generated previously (*41*) and in new studies using different regimens (e.g., injection route, dosage, and adjuvant) potently neutralized the variants. Notably, SApNPs retained their high ID_50_ titers at a dosage as low as 3.3 μg, whereas formulations with the STING and TLR9 agonists further enhanced the I3-01v9 SApNP-induced neutralizing response. While plasma neutralization data may be interpreted with caution due to assay variation (*44*), single-cell–sorted mAbs provided unambiguous evidence of the vaccine-induced bNAb response. Our results revealed that a plethora of NAb lineages were generated upon vaccination, with I3-01v9 SApNP being the most effective at eliciting bNAbs. In addition, our results confirmed the necessity of a prime-boost strategy for eliciting a potent NAb response, regardless of the regimen (e.g., injection route, dosage, and adjuvant). Such an NAb response, once generated, can persist for an extended period of time after vaccination. Although SARS-CoV-2 challenge in relevant animal models gives more accurate assessment of vaccine protection (*91*), NAb titers have been found to be highly predictive of immune protection from symptomatic infections in a large cohort study (*92*). Protein vaccines, despite the well-established records of safety and effectiveness, have yet to be deployed to mitigate the COVID-19 pandemic (*93*–*95*). One protein vaccine, NVX-CoV2373 (micelle-attached spikes formulated with the Matrix-M adjuvant), showed ~90% efficacy in human trials (*19*). Our study indicates that SApNPs displaying 20 stabilized spikes provide a promising protein vaccine candidate that can be used either alone or as a booster for nucleic acid (e.g., mRNA and viral vector) vaccines in the battle against emerging SARS-CoV-2 variants (*11*).

We explored the mechanism of SApNP versus spike vaccines following the previously used strategy to analyze the in vivo behaviors of antigen-attached gold NPs (*73*). In principle, SApNP vaccines must induce long-lasting GCs to facilitate the development of bNAbs. Effective vaccine retention and presentation are critical for inducing and sustaining GC reactions, which, in turn, promote the proliferation and affinity maturation of antigen-specific B cells. Indeed, we found that the I3-01v9 SApNP, our leading vaccine candidate (*41*), elicited sixfold longer retention and fourfold greater accumulation in lymph node follicles than the stabilized S2GΔHR2 spike alone with a prime-boost regimen. This can be attributed to the intrinsic physiological properties of lymph nodes that mediate vaccine trafficking and retention in follicles in a size-dependent manner, which would favor retaining large (>50 nm) VLPs (*73*–*75*, *80*, *96*). Supporting this notion are the TEM images of retained SApNPs aligned on long FDC dendrites, suggesting that such protein NPs can present spike antigens to B cells for rapid initiation and then sustain GC reactions in lymph node follicles for an extended period of time. Specifically, the I3-01v9 SApNP generated 2.4-fold larger GCs and greater numbers of GC B cells (5.2-fold) and T_fh_ cells (4.4-fold) than the soluble S2GΔHR2 spike with the prime-boost regimen. These findings provide quantitative evidence that spike-presenting SApNPs are uniquely suited for inducing long-lived robust GCs in lymph node follicles. Our analyses thus shed light on the mechanism by which the I3-01v9 SApNP can elicit a more effective bNAb response than the soluble spike.

Rational design of next-generation COVID-19 vaccines requires an in-depth understanding of bNAb elicitation (*31*). Superior NAb (but not necessarily bNAb) responses have been reported for several vaccine candidates that use particulate display (*90*, *97*–*104*). The I3-01v9 SApNP elicited a potent bNAb response to four variants, overcoming a major challenge facing the current COVID-19 vaccines. Mechanistic studies of vaccine trafficking, retention, presentation, and GC reactions provided valuable insights into the spike and SApNP-induced immunity (*95*, *105*, *106*). Such knowledge, if can be obtained for other vaccine platforms (e.g., inactivated whole virions, mRNAs, and viral vectors), will facilitate rational selection of the most effective vaccine candidates to mitigate the pandemic and ultimately stop the spread of SARS-CoV-2.

## MATERIALS AND METHODS

### SARS-CoV-2 spike and SApNP vaccine antigens

The design, expression, and purification of a stabilized SARS-CoV-2 spike, S2GΔHR2, and three SApNPs that present either 8 or 20 S2GΔHR2 spikes were described in our recent study (*41*). Briefly, the spike gene of the SARS-CoV-2 isolate Wuhan-Hu-1 (GenBank accession no. MN908947) was modified to include the mutations ^682^GSAGSV^687^ and K986G/V987G, in addition to truncation of the HR2 stalk (ΔE1150-Q1208). The viral capsid protein SHP (Protein Data Bank: 1TD0) was added as a C-terminal trimerization motif to stabilize the S2GΔHR2 trimer, resulting in a soluble S2GΔHR2-5GS-1TD0 spike (*41*). The S2GΔHR2 spike was genetically fused to FR, multilayered E2p, and multilayered I3-01v9 with 5GS, 5GS, and 10GS linkers, respectively, resulting in three S2GΔHR2-presenting SApNPs (*41*). An S2P_ECTO_-5GS-1TD0 spike construct that contained the mutations ^682^GSAGSV^687^ and K986G/V987G but without HR2 deletion (*41*) was included for comparison. All vaccine antigens were transiently expressed in ExpiCHO cells and purified by a CR3022 antibody column and size-exclusion chromatography (SEC) as described previously (*41*). Briefly, ExpiCHO cells were thawed and incubated with ExpiCHO Expression Medium (Thermo Fisher Scientific) in a shaker incubator at 37°C at 135 rotations per minute (rpm) with 8% CO_2_. When the cells reached a density of 10 × 10^6^ ml^−1^, ExpiCHO Expression Medium was added to reduce cell density to 6 × 10^6^ ml^−1^ for transfection. The ExpiFectamine CHO/plasmid DNA complexes were prepared for 100-ml transfection in ExpiCHO cells according to the manufacturer’s instructions. For a given construct, 100 μg of plasmid and 320 μl of ExpiFectamine CHO reagent were mixed in 7.7 ml of cold OptiPRO medium (Thermo Fisher Scientific). After the first feed on day 1, ExpiCHO cells were cultured in a shaker incubator at 33°C at 115 rpm with 8% CO_2_ according to the Max Titer protocol with an additional feed on day 5 (Thermo Fisher Scientific). Culture supernatants were harvested 13 to 14 days after transfection, clarified by centrifugation at 4000 rpm for 25 min, and filtered using a 0.45-μm filter (Thermo Fisher Scientific). The CR3022 antibody column was used to extract SARS-CoV-2 antigens from the supernatants, followed by SEC on a Superdex 200 10/300 GL column (for scaffolded RBD trimers), a Superose 6 16/600 GL column (for the S2GΔHR2 spike, with and without Avi-tag), or a Superose 6 10/300 GL column (for SApNPs). Protein concentration was determined using ultraviolet absorbance at 280nm (UV_280_) with theoretical extinction coefficients.

### Animal immunization and sample collection

Similar immunization protocols were reported in our previous vaccine studies (*41*, *86*, *87*). Briefly, the Institutional Animal Care and Use Committee (IACUC) guidelines were followed for all of the animal studies. BALB/c mice (6 weeks old) were purchased from the Jackson Laboratory and kept in ventilated cages in environmentally controlled rooms at The Scripps Research Institute. The mouse studies were conducted according to the Association for the Assessment and Accreditation of Laboratory Animal Care guidelines, and the protocols were approved by the IACUC. For the immunogenicity study, the mice were intraperitoneally immunized at weeks 0 and 3 with 200 μl of antigen/adjuvant mix containing 5 to 50 μg of vaccine antigen and 100 μl of adjuvant (*41*) or intradermally immunized at weeks 0 and 3 with 80 μl of antigen/adjuvant mix containing 3.3 μg of vaccine antigen and 40 μl of adjuvant. Intradermal immunization was done through injections into four footpads, each with 20 μl of antigen/adjuvant mix. For the mechanistic study of vaccine trafficking, retention, and induced GCs (Figs. 4 to 6), the mice were immunized at weeks 0 and 3 with 80 μl of antigen/adjuvant mix containing 40 μg of vaccine antigen per mouse. Of note, soluble spike was formulated with AddaVax, E2p SApNP was formulated with AddaVax, and I3-01v9 SApNP was formulated with AP for immunization. To visualize the I3-01v9 SApNPs in lymph node tissues using TEM, each mouse was immunized at weeks 0 and 3 with 140 μl of antigen/adjuvant mix containing 100 μg of vaccine antigen (40 μl of adjuvant) into the two hind footpads. Vaccines were intradermally administered into mouse footpads using a 29-gauge insulin needle under 3% isoflurane anesthesia with oxygen. Blood was drawn from the maxillary/facial vein into an EDTA-coated tube 2 weeks after each immunization. Plasma was isolated from blood after centrifugation at 14 000 rpm for 10 min. Plasma was heat-inactivated at 56°C for 30 min, with the supernatant collected after centrifugation at 8000 rpm for 10 min. Plasma was used in pseudovirus neutralization assays to determine vaccine-induced NAb responses. The axillary, brachial, and popliteal sentinel lymph nodes were collected at the end time point for further analysis.

### Experimental adjuvants and formulation

The adjuvants squalene-oil-in-water (AddaVax), AH, AP, 2′3′-c-di-AM(PS)2 (Rp,Rp) (STING ligand), monophosphoryl lipid A from *Salmonella* *minnesota* R595 (TLR4 agonist), imidazoquinoline compound R848 (TLR7/8 agonist), and CpG ODN 1826, Class B (murine) (TLR9 agonist) were purchased from InvivoGen. PIKA, a TLR3 agonist (polyinosinic-polycytidylic acid) with enhanced T cell and antibody responses reported for a phase 1 rabies vaccine trial (*107*), was used as an adjuvant. PIKA was generously provided by Yisheng Biopharma and included in this study as an adjuvant that activates the TLR3 pathway. Macrophage inhibitor CLs (Liposoma BV, catalog no. CP-005-005) were used to eliminate subcapsular sinus macrophages in lymph nodes to promote more robust B cell activation. Mouse immunization was performed to examine the effects of 16 adjuvants or adjuvant combinations on the I3-01v9 SApNP-induced immune response with respect to the nonadjuvanted vaccine (PBS instead of an adjuvant). Vaccine antigen and adjuvants were mixed thoroughly 10 min before immunization. Each mouse was intradermally immunized at weeks 0, 3, and 6 with 120 to 140 μl of antigen/adjuvant mix containing 20 μg of vaccine antigen (I3-01v9 SApNP) and 80 to 100 μl of adjuvant, which was evenly split and injected into four footpads. The adjuvant dose was chosen according to the manufacturer’s recommendation, specifically, 40 μl per mouse for AddaVax, AH and AP, 40 μg per mouse for STING agonist, 40 μl per mouse for TLR3 agonist, 10 μg per mouse for TLR4 agonist, 40 μg per mouse for TLR7/8 agonist, 40 μg per mouse for TLR9 agonist, and 60 μl per mouse for CLs. Mouse blood was isolated at weeks 5 and 8 after two and three intradermal injections, respectively. Spleens and lymph nodes were harvested at week 8 for immunological analyses. Spleen samples were ground through a 70-μm cell strainer to release splenocytes into a cell suspension. Splenocytes were spun down at 400*g* for 10 min, washed with PBS, and treated with the ammonium-chloride-potassium lysing buffer (Lonza). Splenocytes were then frozen with 3 ml of Bambanker freezing medium.

### SARS-CoV-2 pseudovirus neutralization assay

The SARS-CoV-2-pp neutralization assays were described in our previous study (*41*). Briefly, SARS-CoV-2-pps were generated by the cotransfection of human embryonic kidney (HEK) 293T cells with the HIV-1 pNL4-3.lucR-E- plasmid (obtained from the National Institutes of Health AIDS reagent program; www.aidsreagent.org/) and the expression plasmid encoding the *S* gene of five SARS-CoV-2 strains, including the wild-type Wuhan-Hu-1 strain (GenBank accession no. MN908947), three VOCs [global initiative on sharing all influenza data (GISAID) accession no. EPI_ISL_601443, EPI_ISL_678597, and EPI_ISL_792680 for B.1.1.7, B.1.351, and P.1, respectively], and B.1.617_Rec_, a reconstituted strain based on a detailed analysis of the B.1.617 lineage (*11*). The HEK293T-hACE2 cell line (catalog no. NR-52511) and pcDNA3.1(-) vector containing the *S* gene of the wild-type Wuhan-Hu-1 strain (catalog no. NR52420) were requested from the BEI Resources (www.beiresources.org/) on 23 September 2020 and used in the pseudovirus neutralization assays (*43*). On the basis of sequence alignment, spike mutations were incorporated into the *S* gene of the Wuhan-Hu-1 strain (catalog no. NR52420) to create respective expression plasmids for B.1.1.7, B.1.351, P.1, and B.1.617_Rec_. For B.1.617_Rec_, G142D, L452R, E484Q, D614G, and P681R were included as representative spike mutations in this SARS-CoV-2 lineage (*11*). SARS-CoV-2-pp neutralization by immunized mouse plasma and human or mouse mAbs was performed according to our previously described protocol (*41*). Using the same cotransfection expression system as described above for the SARS-CoV-2-pps, we produced pseudoviruses carrying the MLV Env, MLV-pps, for use as a negative control (*41*). Percent neutralization data were analyzed using GraphPad Prism 9.1.2 software. ID_50_/IC_50_ values were calculated using constraints for percent neutralization (0 to 100%), whereas unconstrained neutralization plots are shown in Fig. 1 and figs. S1 to S3.

### Enzyme-linked immunosorbent assay

Each well of a Costar 96-well assay plate (Corning) was first coated with 50 μl of PBS containing 0.2 μg of the appropriate antigens. The plates were incubated overnight at 4°C and then washed five times with wash buffer containing PBS and 0.05% (v/v) Tween 20. Each well was then coated with 150 μl of blocking buffer consisting of PBS and blotting-grade blocker (40 mg/ml; Bio-Rad). The plates were incubated with blocking buffer for 1 hour at room temperature and then washed five times with wash buffer. Mouse mAbs, in the IgG form, were diluted in blocking buffer to a maximum concentration of 10 μg/ml followed by a 10-fold dilution series. For each dilution, a total volume of 50 μl was added to the appropriate wells. Each plate was incubated for 1 hour at room temperature and then washed five times with PBS containing 0.05% Tween 20. A 1:5000 dilution of horseradish peroxidase (HRP)–conjugated goat anti-human IgG antibody (Jackson ImmunoResearch Laboratories) was then made in wash buffer (PBS containing 0.05% Tween 20), with 50 μl of this diluted secondary antibody added to each well. The plates were incubated with the secondary antibody for 1 hour at room temperature and then washed six times with PBS containing 0.05% Tween 20. Last, the wells were developed with 50 μl of tetramethylbenzidene (TMB) (Life Sciences) for 3 to 5 min before stopping the reaction with 50 μl of 2 N sulfuric acid. The resulting plate readouts were measured at a wavelength of 450 nm. The ELISA data were analyzed to calculate EC_50_ values using GraphPad Prism 9.1.2 software.

### Histology, immunostaining, and imaging

The mice were euthanized 2 hours to 8 weeks after a single-dose immunization and 2 hours to 5 weeks after the boost immunization. The axillary, brachial, and popliteal sentinel lymph nodes were isolated for histological analysis. Fresh lymph nodes were rapidly merged into frozen section compound (VWR International, catalog no. 95057-838) in a plastic cryomold (Tissue-Tek at VWR, catalog no. 4565) using liquid nitrogen to preserve antigens on the cell membrane and spike. Lymph node samples were stored at −80°C and sent to the Centre for Phenogenomics (http://phenogenomics.ca) on dry ice for sample processing and imaging. Tissue sections (8 μm) were cut on a cryostat (Cryostar NX70) and collected on charged slides. Sections were postfixed in 10% neutral buffered formalin and permeabilized in PBS containing 0.5% Triton X-100 before immunostaining. Protein Block (Agilent) was used to block nonspecific antibody binding before incubating the sections with primary antibody overnight at 4°C. After washing in tris-buffered saline with 0.1% Tween 20 detergent (TBST), the sections were incubated in fluorophore-conjugated secondary antibodies for 1 hour at room temperature. Lymph node tissue sections were stained with human anti-spike antibody P2B-2F6 (1:50) (*72*) and biotinylated goat anti-human secondary antibody (1:300; Abcam, catalog no. ab7152), followed by streptavidin-HRP reagent (Vectastain Elite ABC-HRP Kit, Vector, catalog no. PK-6100) and diaminobenzidine (DAB) (ImmPACT DAB, Vector, catalog no. SK-4105) to study the distribution and retention of the soluble S2GΔHR2 spike alone and S2GΔHR2 spike-presenting E2p and I3-01v9 SApNPs. For immunofluorescent staining, tissue sections were stained for FDCs using anti-CD21 antibody (1:1800; Abcam, catalog no. ab75985) followed by anti-rabbit secondary antibody conjugated with Alexa Fluor 555 (1:200; Thermo Fisher Scientific, catalog no. A21428), stained for B cells using anti-B220 antibody (1:100; eBioscience, catalog no. 14-0452-82) followed by anti-rat secondary antibody conjugated with Alexa Fluor 674 (1:200; Thermo Fisher Scientific, catalog no. A21247), and stained for subcapsular sinus macrophages using anti-sialoadhesin (CD169) antibody (1:600; Abcam, catalog no. ab53443) followed by anti-rat secondary antibody conjugated with Alexa Fluor 488 (1:200; Abcam, catalog no. ab150165). GC B cells were labeled using rat anti-GL7 antibody [fluorescein isothiocyanate (FITC); 1:250; BioLegend, catalog no. 144604]. T_fh_ cells were labeled using anti-CD4 antibody (1:100; BioLegend, catalog no. 100402) followed by anti-rat secondary antibody conjugated with Alexa Fluor 488 (1:1000; Abcam, catalog no. ab150165) and Bcl6 antibody (1:300; Abcam, catalog no. ab220092) and by anti-rabbit secondary antibody conjugated with Alexa Fluor 555 (1:1000; Thermo Fisher Scientific, catalog no. A21428). Nuclei were then counterstained with 4′,6-diamidino-2-phenylindole (100 ng/ml; Sigma-Aldrich, catalog no. D9542). The stained tissue sections were scanned using an Olympus VS-120 slide scanner and imaged using a Hamamatsu ORCA-R2 C10600 digital camera for all bright-field and fluorescent images. Bright-field images of stained S2GΔHR2 spike and S2GΔHR2 spike-presenting SApNPs in lymph node follicles and fluorescent images of GCs were quantified using ImageJ software (*108*).

### EM analysis of protein NPs and lymph node tissues

Electron microscopy (EM) analysis was performed by the Core Microscopy Facility at The Scripps Research Institute. For the negative staining EM analysis of protein NPs, the S2GΔHR2-10GS-I3-01v9-L7P SApNP samples were prepared at a concentration of 0.01 mg/ml. Carbon-coated copper grids (400 mesh) were glow-discharged, and 10 μl of each sample was adsorbed for 2 min. Excess sample was wicked away, and grids were negatively stained with 2% uranyl formate for 2 min. Excess stain was wicked away, and the grids were allowed to dry. For the EM analysis of mouse tissues, the lymph nodes were dissected from each animal and immersed in oxygenated 2.5% glutaraldehyde and 4% paraformaldehyde in 0.1 M Na cacodylate buffer (pH 7.4) fixative overnight at 4°C. After washing in 0.1 M sodium cacodylate buffer, the tissue samples were postfixed in buffered 1% osmium tetroxide and 1.5% potassium ferrocyanide for 1 to 1.5 hours at 4°C, rinsed in the same buffer, and then stained en bloc with 0.5% uranyl acetate overnight at 4°C. The tissue samples were washed in double-distilled H_2_O and dehydrated through a graded series of ethanol followed by acetone, infiltrated with LX-112 (Ladd) epoxy resin, and polymerized at 60°C. Ultrathin lymph node sections (at 70 nm thickness) were prepared for imaging. Samples were analyzed at 80 kV with a Talos L120C TEM (Thermo Fisher Scientific), and images were acquired with a CETA 16M CMOS camera.

### Lymph node disaggregation, cell staining, and flow cytometry

GC reactions, including the percentage of GC B cells (GL7^+^B220^+^) and T_fh_ cells (CD3^+^CD4^+^CXCR5^+^PD-1^+^), and the number of GC B cells and T_fh_ cells were studied by flow cytometry (fig. S5A). The mice were euthanized 2, 5, and 8 weeks after a single-dose immunization and 2 and 5 weeks after the boost immunization. Fresh axillary, brachial, and popliteal sentinel lymph nodes were collected and mechanically disaggregated. These lymph node samples were merged in enzyme digestion solution containing 958 μl of Hanks’ balanced salt solution (HBSS) buffer (Thermo Fisher Scientific, catalog no. 14185052), 40 μl of collagenase IV (10 mg/ml; Sigma-Aldrich, catalog no. C5138), and 2 μl of deoxyribonuclease (10 mg/ml; Roche, catalog no. 10104159001) in an Eppendorf tube. After incubation at 37°C for 30 min, lymph node samples were filtered through a 70-μm cell strainer and spun down at 400*g* for 10 min. The supernatant was discarded, and the cell pellet was resuspended in HBSS blocking solution containing 0.5% (w/v) bovine serum albumin and 2 mM EDTA. The nonspecific binding of Fc receptors was blocked using anti-CD16/32 antibody (BioLegend, catalog no. 101302) on ice for 30 min. Cocktail antibodies, Zombie NIR live/dead stain (BioLegend, catalog no. 423106), Brilliant Violet 510 anti-mouse/human CD45R/B220 antibody (BioLegend, catalog no. 103247), FITC anti-mouse CD3 antibody (BioLegend, catalog no. 100204), Alexa Fluor 700 anti-mouse CD4 antibody (BioLegend, catalog no. 100536), phycoerythrin (PE) anti-mouse/human GL7 antibody (BioLegend, catalog no. 144608), Brilliant Violet 605 anti-mouse CD95 (Fas) antibody (BioLegend, catalog no. 152612), Brilliant Violet 421 anti-mouse CD185 (CXCR5) antibody (BioLegend, catalog no. 145511), and PE/Cyanine7 anti-mouse CD279 (PD-1) antibody (BioLegend, catalog no. 135216) were then mixed with the cells and placed on ice for 30 min. After washing cells with HBSS blocking solution after antibody staining, the samples were fixed using 1.6% paraformaldehyde (Thermo Fisher Scientific, catalog no. 28906) in HBSS on ice for 30 min. The cell samples were stored in HBSS blocking solution for the flow cytometry study. Sample events were acquired by a five-laser BD Biosciences LSR II analytical flow cytometer with BD FACSDiva software version 6.0 at the Core Facility of The Scripps Research Institute. The data were further processed using FlowJo 10 software.

### DC production, T cell culture, activation, and flow cytometry analysis

Mouse bone marrow was cultured in RPMI 1640 medium containing 10% fetal bovine serum (FBS) and recombinant mouse Fms-like tyrosine kinase 3 ligand (Flt3L, 50 ng/ml) and stem cell factor (SCF, 10 ng/ml) for 9 days as previously described (*109*). To induce DC activation, immature DCs were incubated with lipopolysaccharide (100 ng/ml) and R848 (Resiquimod, 100 ng/ml) overnight, which activated TLR4 or TLR7/8 signaling, respectively. Cells were harvested for the experiments. CD11c^+^ DCs were sorted using magnetic beads (Miltenyi-Biotec, CA). Splenic mononuclear cells from each group of immunized mice were cultured in the presence of DCs pulsed with or without I3-01v9 SApNP (1 × 10^−7^ mM) in complete Iscove’s modified Dulbecco’s medium (IMDM) containing IL-2 (5.0 ng/ml). Cells were collected 16 hours later for intracellular cytokine staining and flow cytometry. All antibodies used for immunofluorescence staining were purchased from eBioscience (San Diego, CA), BioLegend (San Diego, CA), or BD Biosciences (San Jose, CA). Magnetic microbead-conjugated streptavidin was purchased from Miltenyi-Biotec (Auburn, CA). Recombinant human IL-2 protein was purchased from R&D Systems (Minneapolis, MN). Recombinant mouse Flt3L and mouse SCF were purchased from Shenandoah Biotech (Warwick, PA). Cells were stained with appropriate concentrations of mAbs. Dead cells were excluded using Fixable Viability Dye (eBioscience, CA). Flow cytometry was performed using LSRII (BD Bioscience, CA).

### Bulk and single-cell sorting of SARS-CoV-2 antigen-specific mouse B cells

Spleens or lymph nodes were harvested from mice 15 days after the last immunization, and the cell suspension was prepared. Dead cells were excluded by staining with the Fixable Aqua Dead Cell Stain kit (Thermo Fisher Scientific, catalog no. L34957). FcγIII (CD16) and FcγII (CD32) receptors were blocked by adding 20 μl of 2.4G2 mAb (BD Pharmigen, catalog no. N553142). The cells were then incubated with 10 μg of a biotinylated RBD-5GS-foldon-Avi trimer or biotinylated S2GΔHR2-5GS-foldon-Avi spike. Briefly, the probes were generated by the biotinylation of Avi-tagged SARS-CoV-2 antigens using biotin ligase BirA according to the manufacturer’s instructions (Avidity). Biotin excess was removed by SEC on either a Superdex 200 10/300 column (GE Healthcare) for the RBD probe or a HiLoad Superose 6 16/600 column (GE Healthcare) for the spike probe. In the SEC profiles, the probe peak was well separated from the peak of biotin ligase (fig. S3A). Cells and biotinylated proteins were incubated for 5 min at 4°C, followed by the addition of 2.5 μl of anti-mouse IgG fluorescently labeled with FITC (Jackson ImmunoResearch Laboratories, catalog no. 115-095-071) and incubated for 15 min at 4°C. Last, 5 μl of premium-grade allophycocyanin (APC)–labeled streptavidin was added to the cells and incubated for 15 min at 4°C. In each step, the cells were washed with 0.5 ml of PBS and the sorting buffer (PBS with 2% FBS). FITC^+^ APC^+^ probe-specific B cells were sorted using MoFloAstrios EQ (Beckman Coulter). For bulk sorting, positive cells were sorted into an Eppendorf microtube with 20 μl of lysis buffer. For single B cell sorting, individual positive cells were sorted into the inner wells of a 96-well plate with 20 μl of pre-reverse transcription lysis mix containing 0.1 μl of NP-40 (Sigma-Aldrich), 0.5 μl of RNAse Inhibitor (Thermo Fisher Scientific), 5 μl of 5× First Strand Buffer, and 1.25 μl of dithiothreitol from the SuperScript IV kit (Invitrogen), with 13.15 μl of H_2_O per well.

### Antibody cloning from Env-specific single B cells and antibody production

The antibody cloning of SARS-CoV-2 antigen-sorted single B cells was conducted as follows. A mix containing 3 μl of Random Hexamers (GeneLink), 2 μl of deoxynucleotide triphosphates, and 1 μl of SuperScript IV enzyme (Thermo Fisher Scientific) was added to each well of a single-cell–sorted 96-well plate that underwent thermocycling according to the program outlined in the SuperScript IV protocol, resulting in 25 μl of complementary DNA (cDNA) for each single cell. cDNA (5 μl) was then added to a polymerase chain reaction (PCR) mix containing 12.5 μl of 2× Multiplex PCR mix (QIAGEN), 9 μl of H_2_O, 0.5 μl of forward primer mix, and 0.5 μl of reverse mouse primer mix (*110*) for HC and KC within each well. A second PCR reaction was then performed using 5 μl of the first PCR as the template and respective mouse primers (*110*) according to the same recipe as the first PCR. The PCR products were run on 1% Agarose gel and those with correct heavy and light chain bands were then used for Gibson ligation (New England Biolabs), cloning into human IgG expression vectors, and transformation into competent cells. Mouse mAbs were expressed by the transient transfection of ExpiCHO cells (Thermo Fisher Scientific) with equal amounts of paired HC and KC plasmids. Antibody proteins were purified from the culture supernatant after 12 to 14 days using Protein A bead columns (Thermo Fisher Scientific).

### NGS and bioinformatics analysis of mouse B cells

Previously, a 5′-rapid amplification of cDNA end (RACE)–PCR protocol was developed for the deep sequencing analysis of mouse B cell repertoires (*69*). In the present study, this protocol was applied to analyze bulk-sorted, RBD/spike-specific mouse B cells. Briefly, 5′-RACE cDNA was obtained from bulk-sorted B cells of each mouse with the SMART-Seq v4 Ultra Low Input RNA Kit for Sequencing (TaKaRa). The IgG PCRs were set up with Platinum Taq High-Fidelity DNA Polymerase (Life Technologies) in a total volume of 50 μl, with 5 μl of cDNA as the template, 1 μl of 5′-RACE primer, and 1 μl of 10 μM reverse primer. The 5′-RACE primer contained a Personal Genome Machine (PGM)/S5 P1 adaptor, and the reverse primer contained a PGM/S5 A adaptor. The mouse 3′-C_γ_1-3/3′-C_μ_ inner primers and 3′-mC_κ_ outer primer (*110*) were adapted as reverse primers for the 5′-RACE PCR processing of HC and KC. A total of 25 cycles of PCR were performed, and the expected PCR products (500 to 600 bp) were gel purified (QIAGEN). NGS was performed on the Ion S5 GeneStudio system. Briefly, HC and KC libraries from the same mouse were quantitated using a Qubit 2.0 Fluorometer with the Qubit dsDNA HS Assay Kit and then mixed at a 3:1 ratio before being pooled with antibody libraries of other mice at an equal ratio for sequencing. Template preparation and (Ion 530) chip loading were performed on Ion Chef using the Ion 520/530 Ext Kit, followed by sequencing on the Ion S5 system with default settings (*86*). The mouse antibodyomics pipeline (*69*) was used to process raw NGS data; to derive quantitative profiles for germline gene frequency, the degree of SHM, and CDR3 loop length distribution; and to generate 2D divergence/identity plots to visualize mAbs in their respective repertoires (*86*).

### Statistical analysis

Data were collected from four to seven mice per group. All of the statistical analyses were performed and graphs were generated using GraphPad Prism 9.1.2 software. In the analysis of vaccine-induced plasma neutralization, different vaccine groups were compared using one-way analysis of variance (ANOVA), whereas, for a given vaccine group, ID_50_ titers of the same plasma sample against different variants were compared using repeated measures one-way ANOVA. In both cases, they were followed by Dunnett’s multiple comparison post hoc test. For the vaccine accumulation and GC study, different vaccine groups were compared using one-way ANOVA, followed by Tukey’s multiple comparison post hoc test. Statistical significance was indicated as the following: not significant, *P < 0.05, **P < 0.01, ***P < 0.001, and ****P < 0.0001.

## Notes

View/request a protocol for this paper from *Bio-protocol*.
