# Supplementary material for: Mechanism of a COVID-19 nanoparticle vaccine candidate that elicits a broadly neutralizing antibody response to SARS-CoV-2 variants
Source: Sci Adv. 2021 Oct 20;7(43):eabj3107. doi: 10.1126/sciadv.abj3107 (PMC8528426; doi:10.1126/sciadv.abj3107)
Supplement: Supplementary file 1 — Figs. S1 to S10 [file sciadv.abj3107_sm.pdf]

Science Advances
AAAS

Supplementary Materials for

Mechanism of a COVID-19 nanoparticle vaccine candidate that elicits a broadly neutralizing antibody response to SARS-CoV-2 variants

Yi-Nan Zhang, Jennifer Paynter, Cindy Sou, Tatiana Fourfouris, Ying Wang, Ciril Abraham, Timothy Ngo, Yi Zhang, Linling He, Jiang Zhu*

*Corresponding author. Email: jiang@scripps.edu

Published 20 October 2021, *Sci. Adv.* **7**, eabj3107 (2021)
DOI: 10.1126/sciadv.abj3107

This PDF file includes:

Figs. S1 to S10

fig. S1

A **Week 5 mouse plasma neutralization against SARS-CoV-2 strains Wuhan-Hu-1, B.1.1.7, B1.351, P.1 and B.1.617$_{Rec}$**

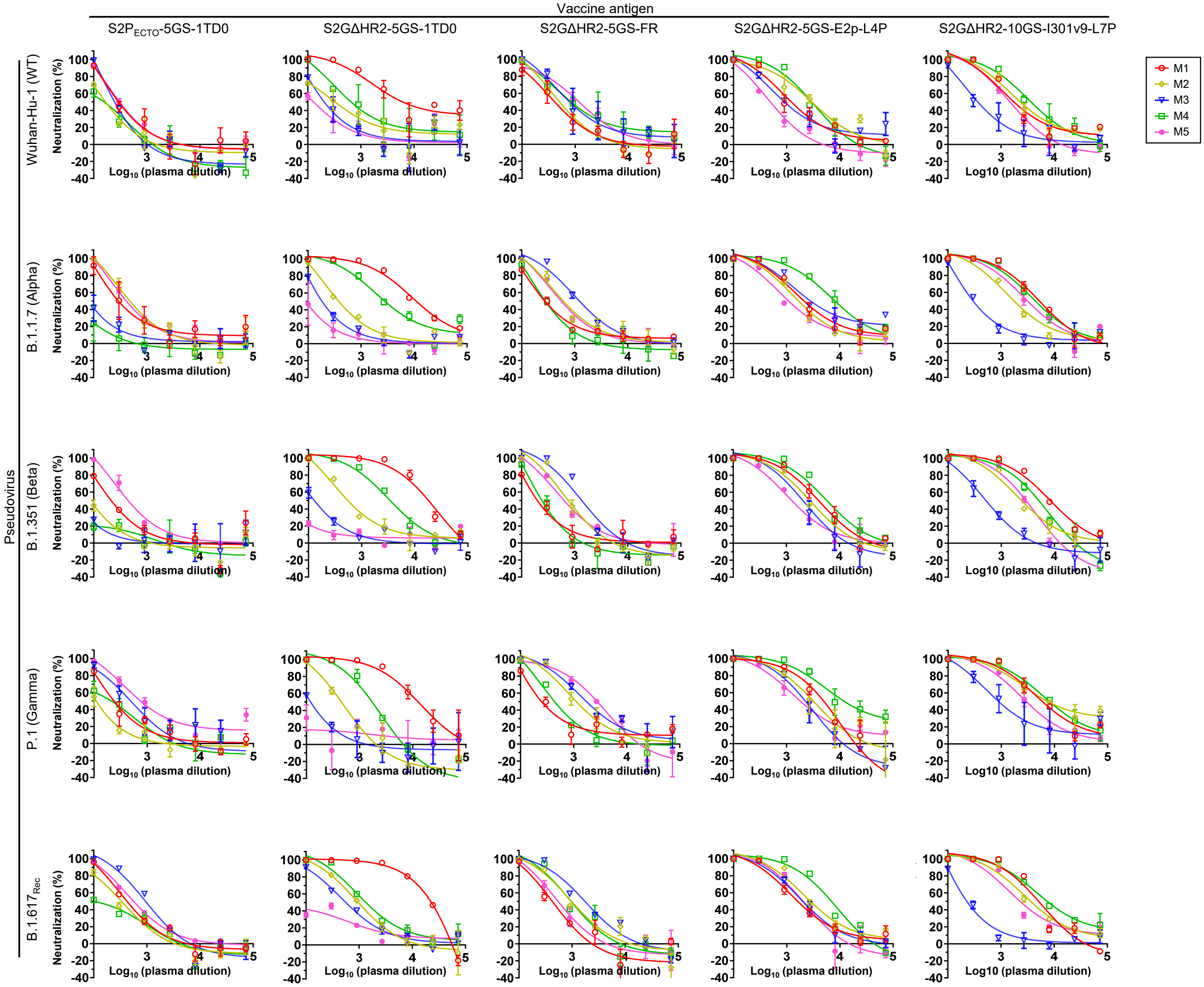

B **Week 5 mouse plasma neutralization ID$_{50}$ titers**

	Wuhan-Hu-1 (WT)					B.1.1.7 (Alpha)					B.1.351 (Beta)					P.1 (Gamma)					B.1.617$_{Rec}$				
Vaccine antigen	M1	M2	M3	M4	M5	M1	M2	M3	M4	M5	M1	M2	M3	M4	M5	M1	M2	M3	M4	M5	M1	M2	M3	M4	M5
S2PECTO-5GS-1TD0 (50 μg, i.p.)	316	150	233	123	326	377	541	<100	<100	487	223	<100	<100	<100	506	274	<100	465	186	967	443	302	913	151	610
S2GΔHR2-5GS-1TD0 (50 μg, i.p.)	7719	370	227	911	131	11868	444	202	3252	<100	17560	654	123	3550	<100	12563	387	101	1467	<100	17029	980	607	1530	124
S2GΔHR2-5GS-FR (50 μg, i.p.)	425	544	962	1135	1154	326	700	1327	293	629	215	764	1353	236	695	316	1244	1978	488	2355	379	994	1701	954	535
S2GΔHR2-5GS-E2p-L4P (50 μg, i.p.)	1442	3020	1107	2849	501	2458	1846	3473	8633	1331	4018	2757	2002	5971	1375	3650	3085	1966	14185	2104	1803	3045	2105	6394	1941
S2GΔHR2-10GS-I301v9-L7P (50 μg, i.p.)	1992	2648	428	3910	1470	5313	1953	355	4715	3337	8641	2602	506	4336	2480	6461	9520	1070	8834	3180	4969	4031	259	8562	2612

C Week 5 mouse plasma neutralization against SARS-CoV-2 strains Wuhan-Hu-1, B.1.1.7, B1.351, P.1 and $B.1.617_{Rec}$

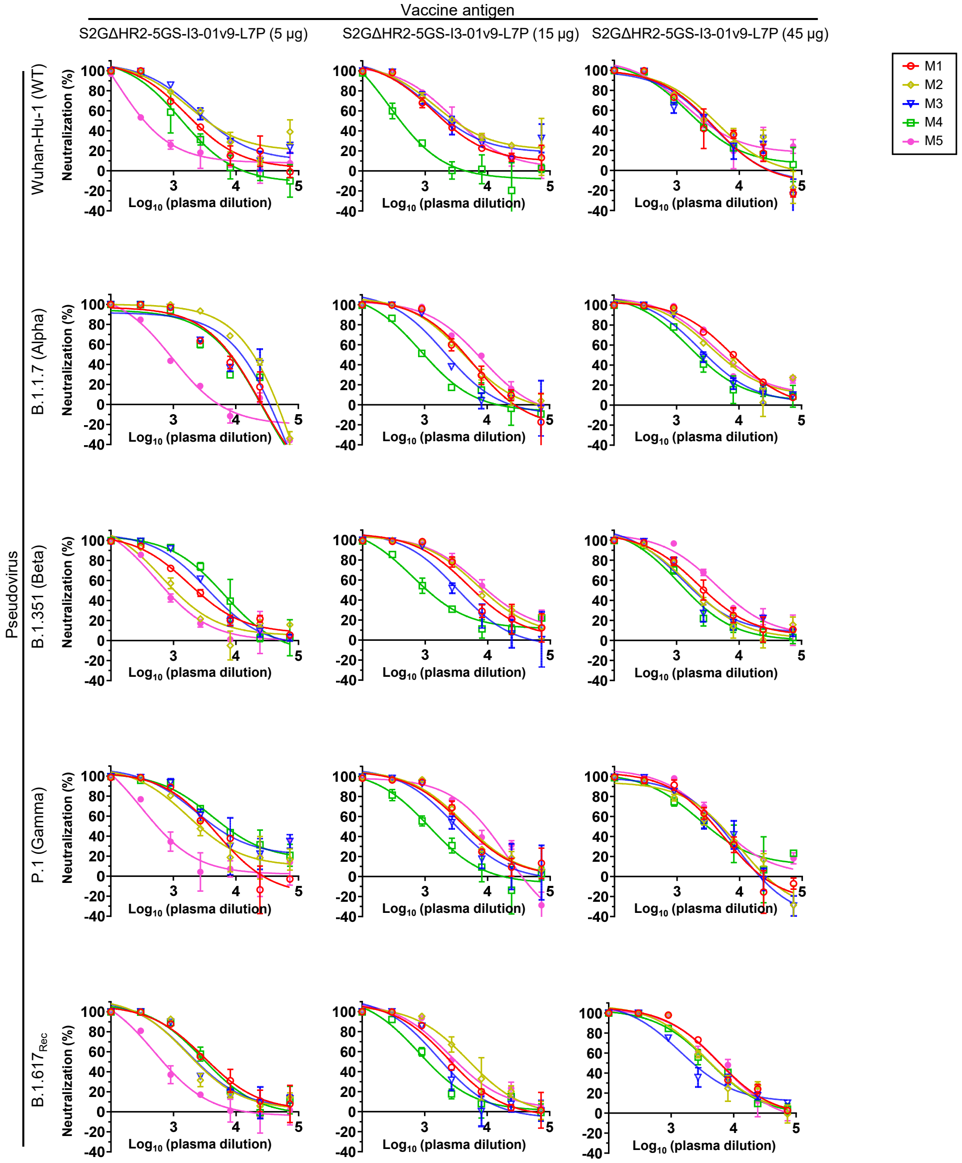

D Week 5 mouse plasma neutralization ID_{50} titers

	Wuhan-Hu-1 (WT)					B.1.1.7 (Alpha)					B.1.351 (Beta)					P.1 (Gamma)					$B.1.617_{Rec}$				
Vaccine antigen	M1	M2	M3	M4	M5	M1	M2	M3	M4	M5	M1	M2	M3	M4	M5	M1	M2	M3	M4	M5	M1	M2	M3	M4	M5
S2GΔHR2-10GS-I3-01v9-L7P (5 µg, i.p.)	2287	4102	4023	1364	455	4806	12463	5425	4080	796	2624	1163	3711	5420	848	3550	2939	5351	7806	620	3911	2467	2591	3412	731
S2GΔHR2-10GS-I3-01v9-L7P (15 µg, i.p.)	2381	3946	2911	425	3493	4050	4354	2280	1011	6750	6220	8930	3425	1290	10607	4560	5007	3325	1143	6478	2721	5353	1941	1220	3513
S2GΔHR2-10GS-I3-01v9-L7P (45 µg, i.p.)	2772	3753	2644	2433	3186	8072	5394	3222	2401	6157	3062	2141	2119	1538	5770	3627	3585	3469	3503	5235	6128	4567	2683	4285	5841

fig. S1

E **Week 5 mouse plasma neutralization against SARS-CoV-2 strains Wuhan-Hu-1, B.1.1.7, B1.351, P.1 and B.1.617$_{Rec}$**

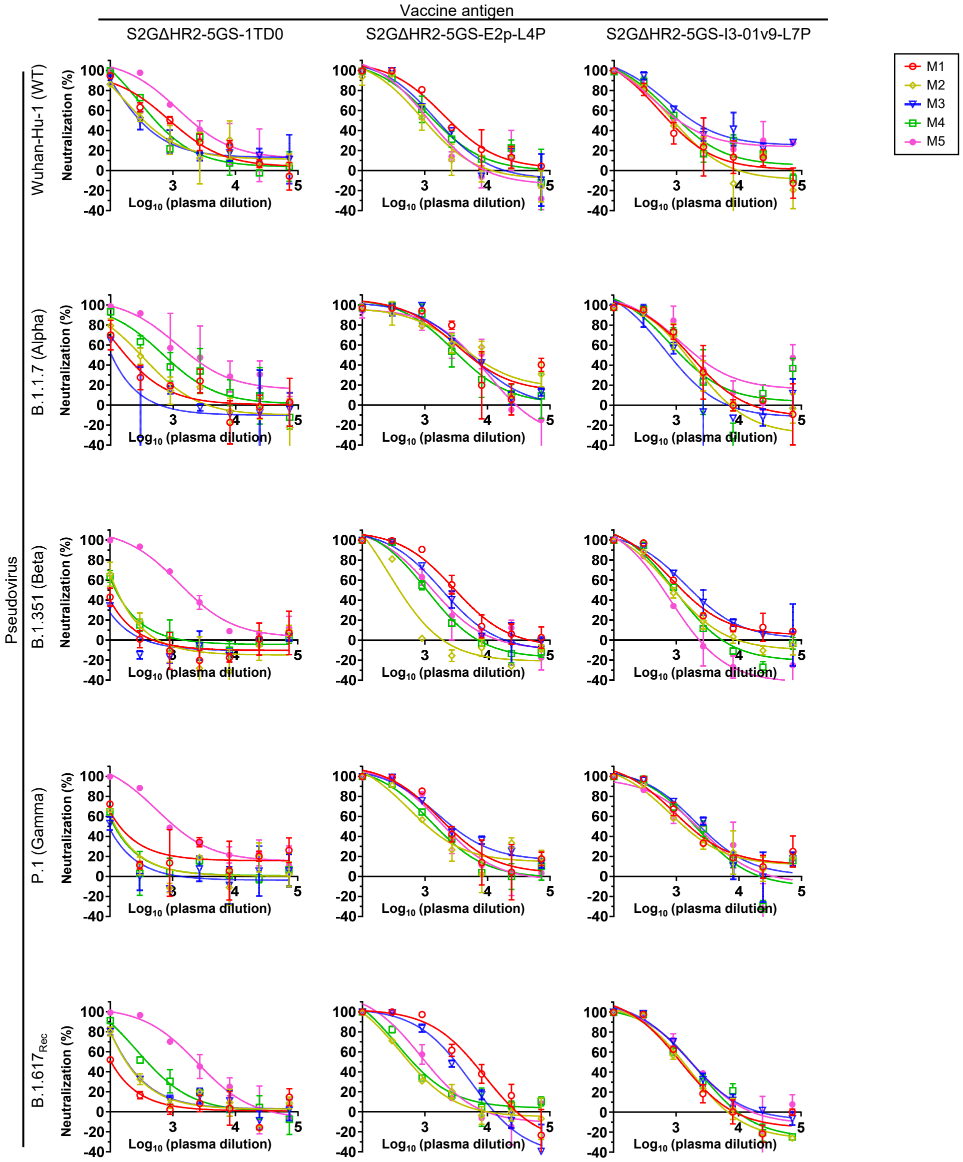

F **Week 5 mouse plasma neutralization ID_{50} titers**

Vaccine antigen	Wuhan-Hu-1 (WT)					B.1.1.7 (Alpha)					B.1.351 (Beta)					P.1 (Gamma)					B.1.617$_{Rec}$				
	M1	M2	M3	M4	M5	M1	M2	M3	M4	M5	M1	M2	M3	M4	M5	M1	M2	M3	M4	M5	M1	M2	M3	M4	M5
S2GΔHR2-5GS-1TD0 (3.3 μg, i.d.)	923	435	416	640	2345	179	278	<100	721	2440	<100	<100	<100	105	1787	146	100	<100	<100	1446	<100	208	213	429	2329
S2GΔHR2-5GS-E2p-L4P (3.3 μg, i.d.)	2680	988	1586	1530	1178	5243	6549	5467	3676	4300	3149	377	1884	1048	1372	2533	1335	3524	1573	2120	4788	599	2385	734	1048
S2GΔHR2-10GS-I3-01v9-L7P (3.3 μg, i.d.)	851	884	2593	1263	1504	1617	1271	776	1267	2292	1418	921	1866	891	522	1923	1616	2530	1980	1968	1171	1140	1746	1460	1696

G **Week 26 mouse plasma neutralization against SARS-CoV-2 Wuhan-Hu-1 isolate**

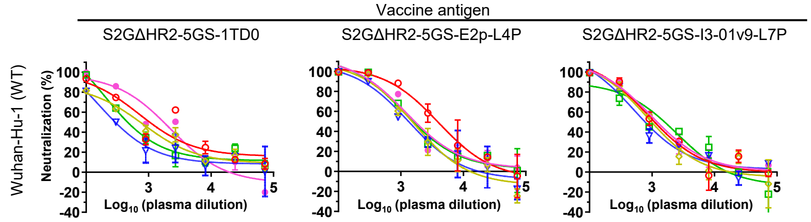

H **Week 26 mouse plasma neutralization ID_{50} titers**

Vaccine antigen	Wuhan-Hu-1 (WT)				
	M1	M2	M3	M4	M5
S2GΔHR2-5GS-1TD0 (3.3 μg, i.d.)	1451	748	348	608	1502
S2GΔHR2-5GS-E2p-L4P (3.3 μg, i.d.)	3800	1446	1309	1728	1844
S2GΔHR2-10GS-I3-01v9-L7P (3.3 μg, i.d.)	1192	1024	901	1147	1323

fig. S1

I **Human monoclonal antibody neutralization against SARS-CoV-2 strains Wuhan-Hu-1, B.1.1.7, B1.351, P.1 and B.1.617$_{Rec}$**

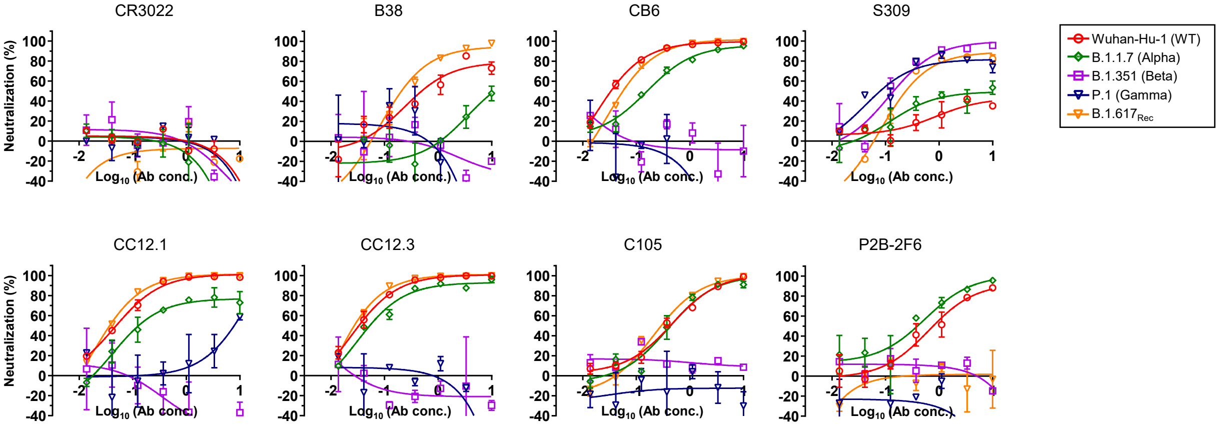

J **Week 5 mouse plasma neutralization against MLV-pp**

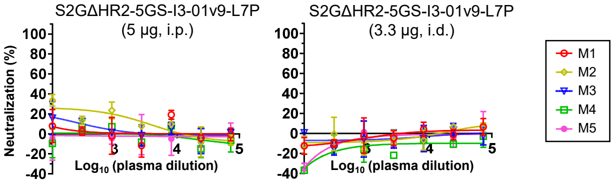

K **Week 5 mouse plasma neutralization ID$_{50}$ titers**

	MLV-pp				
Vaccine antigen	M1	M2	M3	M4	M5
S2GΔHR2-10GS-I3-01v9-L7P (5 μg, i.p.)	<100	<100	<100	<100	<100
S2GΔHR2-10GS-I3-01v9-L7P (3.3 μg, i.d.)	<100	<100	<100	<100	<100

fig. S1. Spike and spike-presenting SApNP vaccine-induced neutralizing antibody responses against the wildtype SARS-CoV-2 strain and four variants. (**A**) Neutralization curves of mouse plasma from 5 vaccine groups at week 5 after 2 intraperitoneal (i.p.) injections. The plasma samples were generated in the previous study (Ref 41), where mice were immunized with 50 μg of adjuvanted vaccine antigen. The five vaccines include two spikes (S2P$_{ECTO}$-5GS-1TD0 and S2GΔHR2-5GS-1TD0) and three SApNPs (S2GΔHR2-5GS-FR, S2GΔHR2-5GS-E2p-LD4-PADRE (L4P), and S2GΔHR2-10GS-I3-01v9-LD7-PADRE (L7P)). (**B**) Summary of ID$_{50}$ titers measured for five SARS-CoV-2 spike-based vaccine groups in (A). (**C**) Neutralization curves of mouse plasma induced by the S2GΔHR2-presenting I3-01v9 SApNP vaccine at week 5 after two i.p. injections with different antigen doses (5 μg, 15 μg, and 45 μg). (**D**) Summary of ID$_{50}$ titers measured for the S2GΔHR2-presenting I3-01v9 SApNP vaccine in (C). (**E**) Neutralization curves of mouse plasma induced by the S2GΔHR2 spike and two S2GΔHR2-presenting SApNPs (E2p and I3-01v9) at week 5 after two intradermal (i.d.) footpad injections (0.8 μg per injection site, totaling 3.3 μg per mouse). (**F**) Summary of ID$_{50}$ titers measured for the three S2GΔSHR2-based vaccines against SARS-CoV-2-pps in (E). (**G**) Neutralization curves of mouse plasma induced by the S2GΔHR2 spike and two S2GΔHR2-presenting SApNPs at week 26 after two i.d. footpad injections (0.8 μg per injection site, totaling 3.3 μg per mouse). (**H**) Summary of ID$_{50}$ titers measured for the three S2GΔSHR2-based vaccines against SARS-CoV-2-pps in (G). (**I**) Neutralization curves of human monoclonal antibodies (mAbs). In (A)-(G), SARS-CoV-2-pps that carry spikes of five strains, including the wildtype Wuhan-Hu-1 strain and four variants, B.1.1.7, B.1.351, P.1, and B.1.617$_{Rec}$ were tested in neutralization assays. (**J**) Neutralization curves of mouse plasma from two S2GΔHR2-presenting I3-01v9 SApNP vaccine groups against MLV-pps. One group was taken from (C), where mice were given 5 μg of adjuvanted antigen via i.p. injection and the other group was taken from (E), where mice were given 3.3 μg of adjuvanted antigen via i.d. injection. (**K**) Summary of ID$_{50}$ titers measured for two S2GΔHR2-presenting I3-01v9 SApNP vaccine groups against MLV-pps. In all tables, the ID$_{50}$ values were calculated with the %neutralization range constrained within 0.0-100.0%. Color coding indicates the level of ID$_{50}$ titer (white: no neutralization; green to red: low to high).

fig. S2

A **Week 2 mouse plasma neutralization against SARS-CoV-2 Wuhan-Hu-1 isolate (antigen for all groups: S2GΔHR2-10GS-I3-01v9-L7P, 20 μg)**

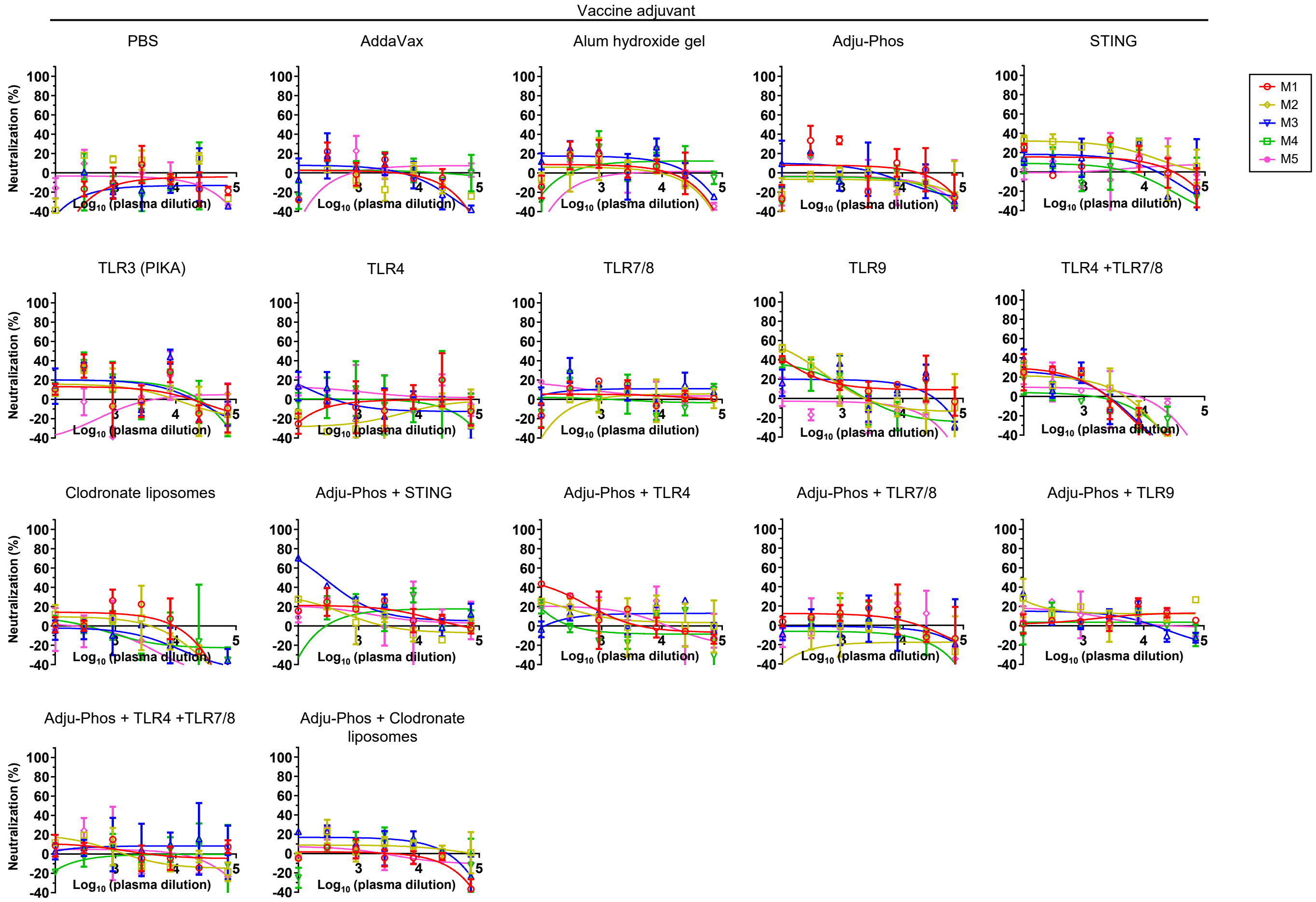

B **Week 2 mouse plasma neutralization ID_{50} titers**

Adjuvant	M1	M2	M3	M4	M5	Average
PBS	<100	<100	<100	<100	<100	0 (± 0)
AddaVax	<100	<100	<100	<100	<100	0 (± 0)
Aluminium hydroxide gel	<100	<100	<100	<100	<100	6 (± 14)
Adju-Phos	<100	<100	<100	<100	<100	3 (± 8)
STING	<100	<100	<100	<100	<100	32 (± 36)
TLR3 (PIKA)	<100	<100	<100	<100	<100	26 (± 17)
TLR4	<100	<100	<100	<100	<100	7 (± 9)
TLR7/8	<100	<100	<100	<100	<100	7 (± 11)
TLR9	<100	125	<100	<100	<100	65 (± 46)
TLR4 +TLR7/8	<100	<100	<100	<100	<100	41 (± 15)
Clodronate liposomes	<100	<100	<100	<100	<100	5 (± 6)
Adju-Phos + STING	<100	<100	272	<100	<100	77 (± 111)
Adju-Phos + TLR4	<100	<100	<100	<100	<100	40 (± 36)
Adju-Phos + TLR7/8	<100	<100	<100	<100	<100	2 (± 4)
Adju-Phos + TLR9	<100	<100	<100	<100	<100	24 (± 21)
Adju-Phos + TLR4 +TLR7/8	<100	<100	<100	<100	<100	9 (± 8)
Adju-Phos + Clodronate liposomes	<100	<100	<100	<100	<100	10 (± 19)

fig. S2

C **Week 5 mouse plasma neutralization against SARS-CoV-2 Wuhan-Hu-1 isolate (antigen for all groups: S2GΔHR2-10GS-I3-01v9-L7P, 20 μg)**

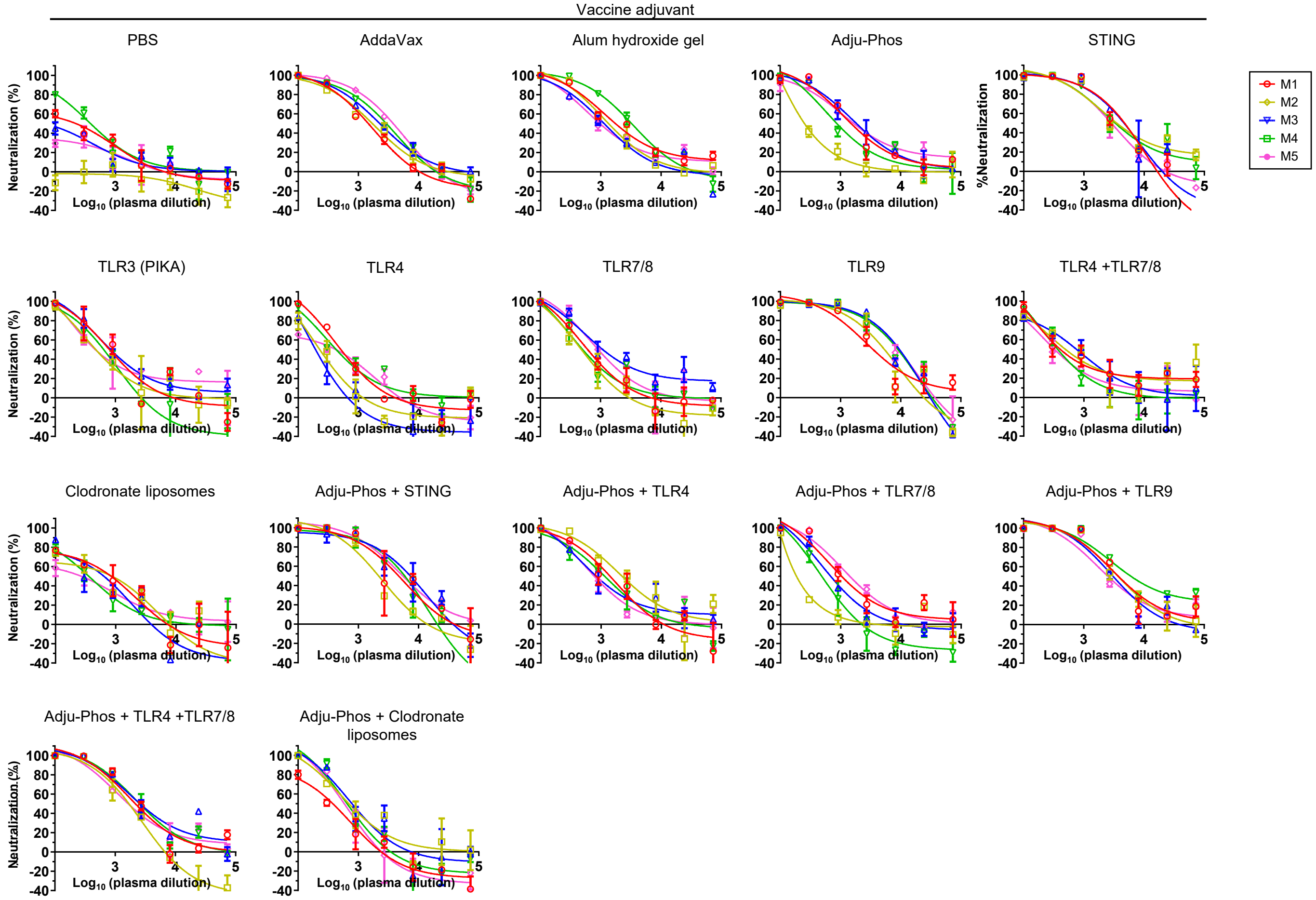

D **Week 5 mouse plasma neutralization ID_{50} titers**

Adjuvant	M1	M2	M3	M4	M5	Average
PBS	210	0	122	393	74	160 (± 151)
AddaVax	1308	1630	2255	2435	3194	2164 (± 735)
Aluminium hydroxide gel	2128	1443	1140	3145	1218	1815 (± 839)
Adju-Phos	1644	305	1983	997	1949	1376 (± 717)
STING	3204	4146	3631	3816	2715	3502 (± 556)
TLR3 (PIKA)	799	525	971	493	722	702 (± 198)
TLR4	511	196	147	486	304	329 (± 165)
TLR7/8	633	453	1496	588	981	830 (± 420)
TLR9	3760	4344	6632	6417	6989	5628 (± 1468)
TLR4 +TLR7/8	637	591	721	399	382	546 (± 150)
Clodronate liposomes	584	461	386	350	277	412 (± 117)
Adju-Phos + STING	4142	2044	5268	3705	5733	4178 (± 1448)
Adju-Phos + TLR4	1236	2360	1060	1114	841	1322 (± 598)
Adju-Phos + TLR7/8	1168	208	721	440	1538	815 (± 540)
Adju-Phos + TLR9	4017	3865	3145	7203	2755	4197 (± 1758)
Adju-Phos + TLR4 +TLR7/8	2293	1330	3045	2677	1940	2257 (± 663)
Adju-Phos + Clodronate liposomes	279	777	795	618	511	596 (± 213)

fig. S2

E **Week 8 mouse plasma neutralization against SARS-CoV-2 Wuhan-Hu-1 isolate (antigen for all groups: S2GΔHR2-5GS-I3-01v9-L7P, 20 μg)**

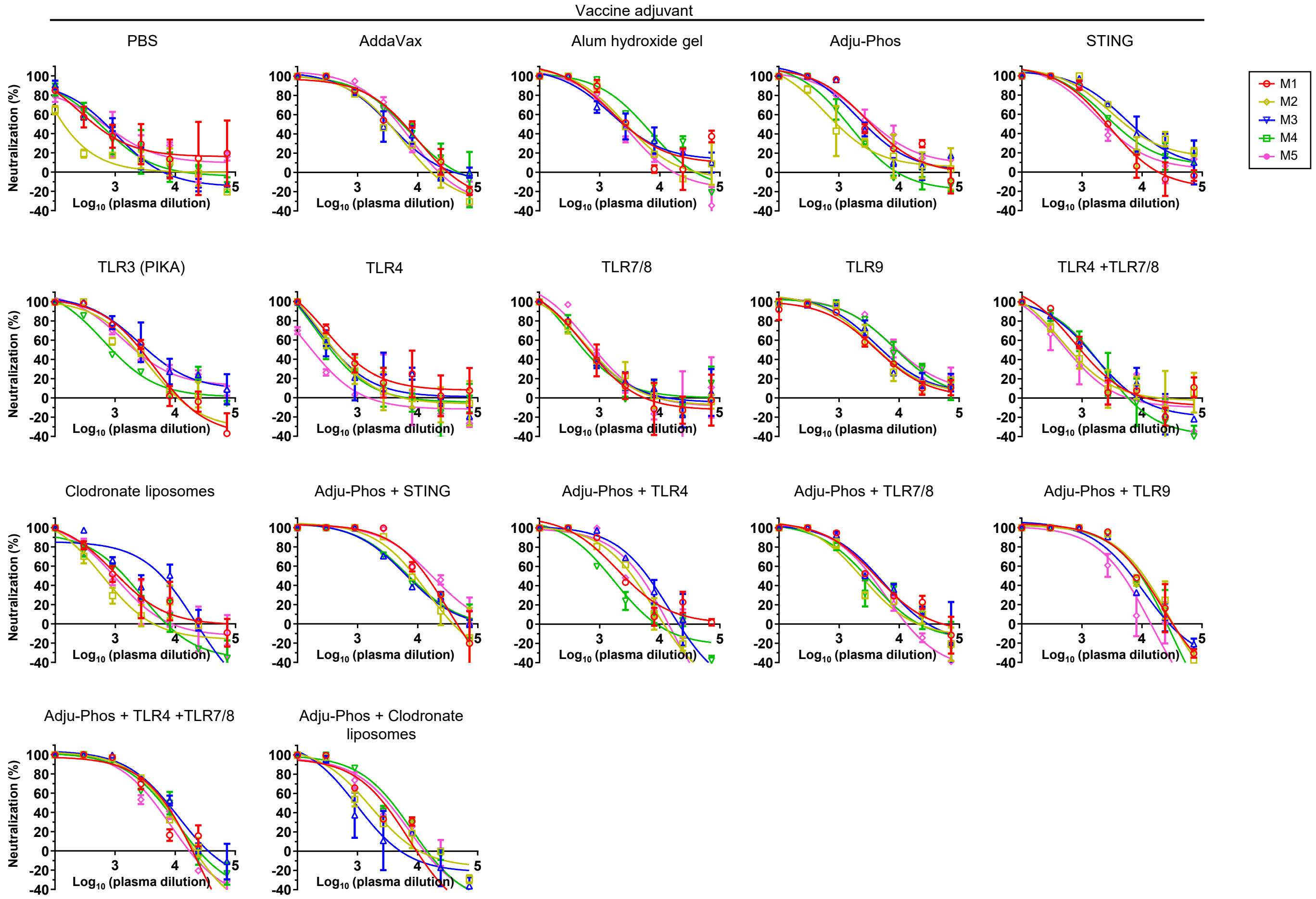

F **Week 8 mouse plasma neutralization ID_{50} titers**

Adjuvant	M1	M2	M3	M4	M5	Average
PBS	585	137	572	524	664	496 (± 207)
AddaVax	4508	2682	3284	5056	4242	3954 (± 958)
Aluminium hydroxide gel	2780	2784	3079	4805	2202	3130 (± 989)
Adju-Phos	3677	1096	2867	1365	4243	2650 (± 1388)
STING	2513	5913	7028	3841	2562	4371 (± 2027)
TLR3 (PIKA)	2156	1720	3934	998	2658	2293 (± 1101)
TLR4	709	444	492	399	150	439 (± 200)
TLR7/8	663	723	737	618	881	724 (± 99)
TLR9	4337	4755	5524	9313	9989	6784 (± 2663)
TLR4 +TLR7/8	982	679	1117	1017	561	871 (± 238)
Clodronate liposomes	1166	569	2467	989	886	1215 (± 733)
Adju-Phos + STING	11103	7838	6767	7236	17251	10039 (± 4375)
Adju-Phos + TLR4	2705	2407	4301	1339	2617	2674 (± 1061)
Adju-Phos + TLR7/8	4012	2216	3825	2489	2614	3031 (± 825)
Adju-Phos + TLR9	7763	7731	5947	6229	2862	6106 (± 1997)
Adju-Phos + TLR4 +TLR7/8	3602	4574	6443	4607	3462	4538 (± 1190)
Adju-Phos + Clodronate liposomes	1587	1351	843	2515	2041	1667 (± 641)

G **Week 5 mouse plasma neutralization against SARS-CoV-2 strains Wuhan-Hu-1, B.1.1.7, B1.351, P.1 and B.1.617$_{Rec}$ (antigen for all groups: S2GΔHR2-5GS-I3-01v9-L7P, 20 μg)**

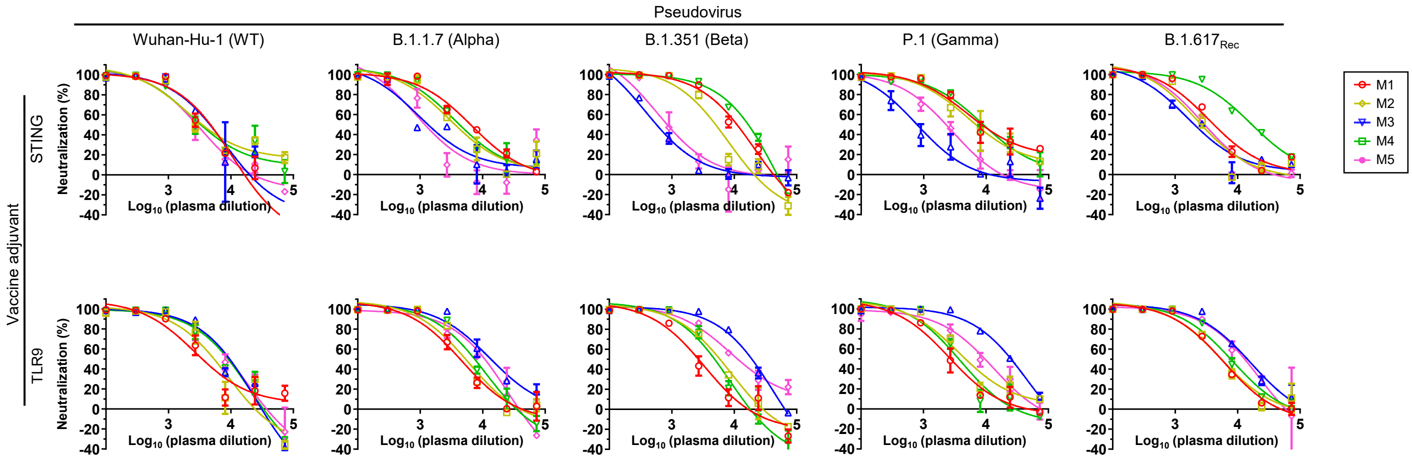

H **Week 5 mouse plasma neutralization ID_{50} titers**

	Wuhan-Hu-1 (WT)					B.1.1.7 (Alpha)					B.1.351 (Beta)					P.1 (Gamma)					B.1.617$_{Rec}$				
Vaccine adjuvant:	M1	M2	M3	M4	M5	M1	M2	M3	M4	M5	M1	M2	M3	M4	M5	M1	M2	M3	M4	M5	M1	M2	M3	M4	M5
STING	3204	4146	3631	3816	2715	6370	3845	1566	4559	1258	8966	4284	618	12385	902	9704	7650	772	9137	2034	4393	2719	2216	17438	3010
TLR9	3760	4344	6632	6417	6989	4315	5234	13994	6628	7660	2420	5156	16634	4019	12345	2770	5077	23415	3615	8614	5319	5588	14219	7722	11231

I **Induction of CD4 T-cell responses against spike-presenting I3-01v9 SApNP**

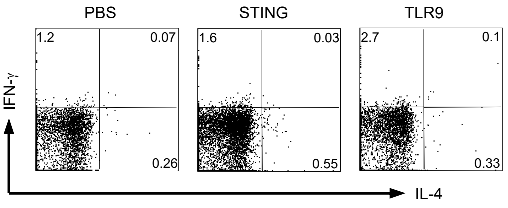

J **Induction of CD8 T-cell responses against spike-presenting I3-01v9 SApNP**

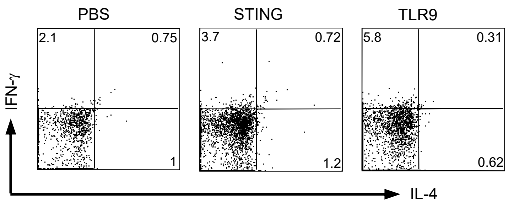

fig. S2. Immune responses against the wildtype SARS-CoV-2 stain and four variants induced by I3-01v9 SApNP formulated with different adjuvants. In this study, the S2GΔHR2-10GS-I3-01v9-L7P NP was either non-adjuvanted (PBS control) or formulated with various adjuvants/adjuvant mixes, resulting in 17 vaccine groups. Mice were immunized at w0, w3 and w6 through intradermal (i.d.) footpad injections (5 μg per injection site, totaling 20 μg per mouse). (**A**) Neutralization curves of mouse plasma against the wildtype Wuhan-Hu-1 strain at week 2 after single injection. (**B**) Summary of ID_{50} titers measured for all vaccine groups in (A). (**C**) Neutralization curves of mouse plasma against the wildtype Wuhan-Hu-1 strain at week 5 after two injections. (**D**) Summary of ID_{50} titers measured for all vaccine groups in (C). (**E**) Neutralization curves of mouse plasma against the wildtype Wuhan-Hu-1 strain at week 8 after three injections. (**F**) Summary of ID_{50} titers measured for all vaccine groups in (E). (**G**) Neutralization curves of mouse plasma from the STING and TLR9 adjuvant groups against the wildtype Wuhan-Hu-1 strain and four variants, B.1.1.7, B.1.351, P.1, and B.1.617$_{Rec}$ at week 5 after two injections. (**H**) Summary of ID_{50} titers measured for the two vaccine groups in (G). Representative flow cytometry graphs of (**I**) CD4 T cell, and (**J**) CD8 T cell responses against spike-presenting I3-01v9 SApNP. In all tables, the ID_{50} values were calculated with the %neutralization range constrained within 0.0-100.0%. Color coding indicates the level of ID_{50} titer (white: no neutralization; green to red: low to high). In (B), (D) and (F), average ID_{50} titer and standard deviation are shown to facilitate the comparison of adjuvant effect between different vaccine formulation groups.

fig. S3

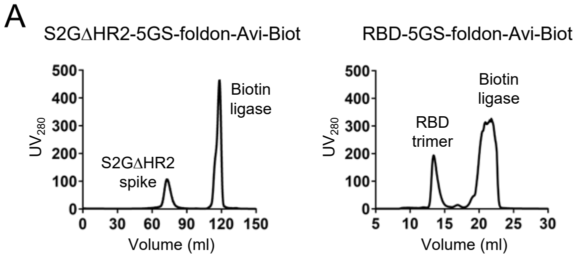

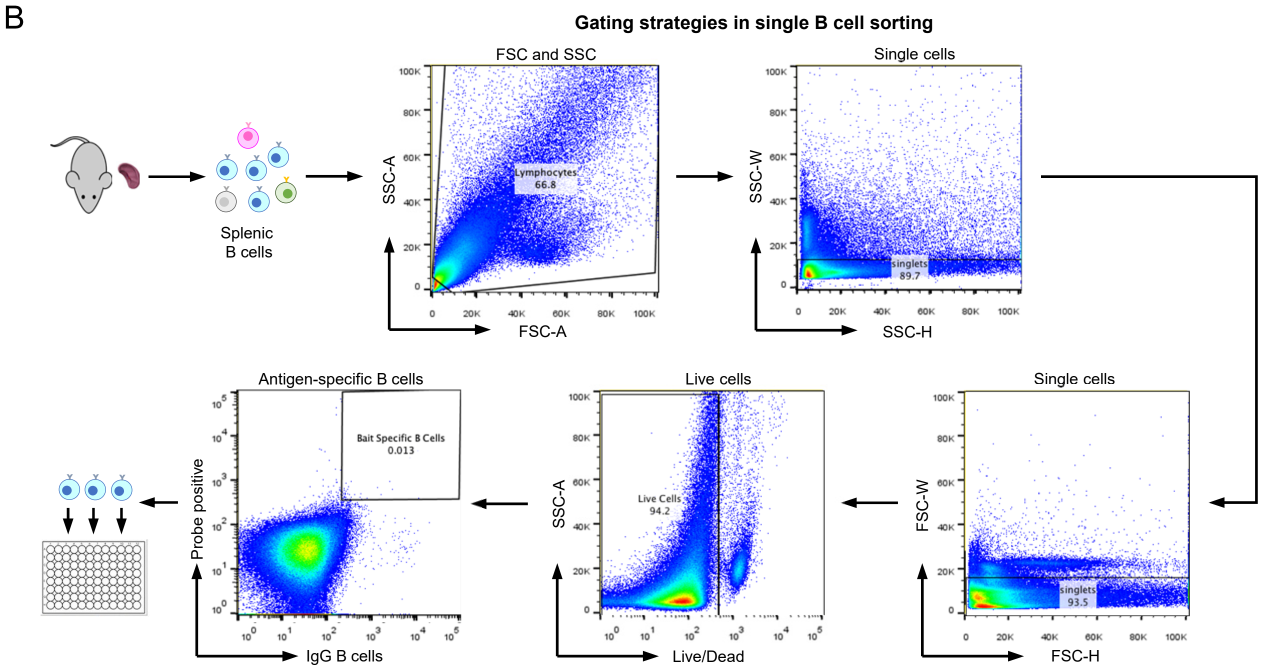

C

Nucleotide and amino acid sequences of monoclonal neutralizing antibodies isolated from M2 in the RBD-5GS-1TD0 group [a].

Heavy chain	Light chain
**>TRBD-R-4HB6 (D-S14A-G5M2-RBD-4HB6)**<br>GAGGTGAAGCTGGAGGAGTCTGGACCTGGCCTAGTGCAGCCCTCACAGAGCCTGTCCATCACCTGCACAGTCTC<br>TGGTTTCTCATTAACTACCTATGGTGTACACTGGGTTCGCCAGTCTCCAGGAAAGGGTCTGGAGTGGCTGGGAGT<br>GATATGGAGTGGTGGAAGCACAGACTATAATGCAGCTTTCATATCCAGACTGAGCATCAGTAAGGACAATTCCAA<br>GAGCCAAGTTTTCTTTGAAATGAACAGTCTGCAAGCTAATGACACAGCCATATATTACTGTGCCAGATGGGGTTAC<br>TGGTACTTCGATGTCTGGGGCGCAGGGACCACGGTCACCGTCTCCTCA	**>TRBD-R-4KB6 (D-S14A-G5M2-RBD-4KB6)**<br>AATATTGTGCTCACCCAATCTCCATCCTCCCTGGCTATGTCAGTAGGACAGAAGGTCACTATGAGCTGCAA<br>GTCCAGTCAGAGCCTTTTAAATAGTAGCAATCAAAAGAACTATTTGGCCTGGTACCAGCAGAAACCAGGA<br>CAGTCTCCTAAAGTTCTGGTATACTTTACATCCACTAGGGAATCTGGGGTCCCTGATCGCTTCATAGGCAG<br>TGGATCTGGGACAGATTTCACTCTTACCATCAGCAGTGTGCAGGCTGAAGACCTGGCAGATTACTTCTGT<br>CAGCAACATTATAGCACTCCGCTCACGTTCGGTGCTGGGACCAAGCTGGAAATAAAA
**>TRBD-R-4HF3 (D-S14A-G5M2-RBD-4HF3)**<br>AAAGTGCAGCTGTTGGAGACTGGGGCTGAGCTGGTGAAGCCTGGGGCCTCAGTGAAGATGTCCTGCAAGGCTTC<br>TGGCTACACATTTACCAGTTCCAATATACACTGGATAAAGCAGACACCTGGACAGGGCCTGGAATGGATTGGAGC<br>TATTTATCCAGGAAATGGTGATACTTCCTACACTCAGAAGTTCAAAGGCAAGGCCACATTGACTGCAGACAAATCC<br>TCCAGCACAGCCTACATGCAGCTCAGCAGCCTGACATCTGAGGACTCTGCGGTCTATTACTGTGCCCTCGACGAT<br>TACTATGCTTTGGACTACTGGGGTCAAGGAACCACGGTCACCGTCTCCTCA	**>TRBD-R-4KF3 (D-S14A-G5M2-RBD-4KF3)**<br>GACATCCTGATGACCCAATCTCCATCCTCCCTAGCTGTGTCAGTTGGAGAGAAGGTTGCTATGAGCTGCA<br>AGTCCAGTCAGAGCCTTTTATACAGTAGCAATCAGAAGAACTACTTGGCCTGGTACCAGCAGAAACCAGG<br>GCAGTCTCCTAAACTGCTGATTTACTGGGCATCCACTAGGGAATCTGGGGTCCCTGATCGCTTCACAGGC<br>AGTGGATCTGGGACAGATTTCACTCTCACCATCAGCAGTGTGAAGGCTGAAGACCTGGCTGTTTATTACT<br>GTCAGCAATATTATAGCTATCCGTGGACGTTCGGTGGAGGCACCAAGCTGGAAATCAAA
**>TRBD-R-4HG5 (D-S14A-G5M2-RBD-4HG5)**<br>GAGGTGAAGCTGGTGGAGTCTGGGACTGTGCTGGCAAGGCCTGGGGCTTCAGTGAAGATGTCCTGCAAGGCTT<br>CTGGCTACACCTTTACCAGCTACTGGATGCACTGGGTAAAACAGAGGCCTGGACGGGGTCTGGAATGGATTGGC<br>GCTATTTATCCTGGAAATAGTGATACTAACTATAACCAGAAGTTCAAGGGCAAGGCCAAACTGACTGCAGTCACAT<br>CCACCAGCACTGCCTACATGGACCTCAGCAGCCTGACAAATGAGGACTCTGCGGTCTATTACTGTACAAGAGATT<br>ACTACGGCTACGGGAACTACTGGGGCCAAGGCACCACTCTCACAGTCTCCTCA	**>TRBD-R-4KG5 (D-S14A-G5M2-RBD-4KG5)**<br>AAAATTGTGCTCACTCAGTCTCCAGCCTCCCTATGTGCATCTGTGGGAGAAACTGTCACCATCACATGTCG<br>AGCAAGTGAGAATATTTACAGTTATTTAGCATGGTATCAGCAGAAACAGGGAAAATCTCCTCAGCTCCTGG<br>TCTATAATGCAAAAACCTTAGCAGAAGGTGTGCCATCAAGGTTCAGTGGCAGTGGATCAGGCACACAGTT<br>TTCTCTGAAGATCAACAGCCTGCAGCCTGAAGATTTTGGGAGTTATTACTGTCAACATCATTATGGTACTC<br>CGCTCACGTTCGGTGCTGGGACCAAGCTGGAAATAAAA
**>TRBD-R-5HB9 (D-S14A-G5M2-RBD-5HB9)**<br>GACGTGAAGCTGGTGGAGTCTGGGCCTCAGCTGGTTAGGCCTGGGGCTTCAGTGAAGATATCCTGCAAGGCTTC<br>TGGTTACTCATTCACCAGCTACTGGATGCAGTGGTTGAAGCAGAGGCCTGGACAAGGTCTTGAGTGGATTGGCAT<br>GATTGATCCTTCCGATAGTGAAACTAGGTTAAATCAGAAGTTCAAGGACAAGGCCACATTGACTGTAGACAAATCC<br>TCCAGCACAGCCCACATGCAACTCAGCAGCCCGACATCTGAGGACTCTGCGGTCTATTATTGTGCAAGAAGGGA<br>TTACTACGGCAATAATCCCTTTGACTTCTGGGGCCAAGGCACCACTCTCACAGTCTCCTCA	**>TRBD-R-5KB9 (D-S14A-G5M2-RBD-5KB9)**<br>GACATTGTGATGTCACAGTCTCCACCCTCTGTACCTGTCACTCCTGGAGAGTCAGTATCCATCTCCTGCAG<br>GTCTAATAAGAGTCTCCTGCATAGTAATGGCAACACTTACTTGTATTGGTTCCTGCAGAGGCCAGGCCAGT<br>CTCCTCAGCTCCTGATATTTCGGATGTCCAACCTTGCCTCAGGAGTCCCAGACAGGTTCAGTGGCAGTGG<br>GTCAGGAACTGCTTTCACACTGAGAATCAGTAGAGTGGAGGCTGAGGATGTGGGTGTTTATTACTGTATG<br>CAACATCTAGAATATCCGTACACGTTCGGAGGGGGGACCAAGCTGGAAATAAAA
**>TRBD-R-4HB6 (D-S14A-G5M2-RBD-4HB6)**<br>EVKLEESGPGLVQPSQSLSITCTVSGFSLTTYGVHWVRQSPGKGLEWLGVIWSGGSTDYNAAFISRLSISKDNSKSQV<br>FFEMNSLQANDTAIYYCARWGYWYFDVWGAGTTVTVSS	**>TRBD-R-4KB6 (D-S14A-G5M2-RBD-4KB6)**<br>NIVLTQSPSSLAMSVGQKVTMSCKSSQSLLNSSNQKNYLAWYQQKPGQSPKVLVYFTSTRESGVPDRFIGSG<br>SGTDFTLTISSVQAEDLADYFCQQHYSTPLTFGAGTKLEIK
**>TRBD-R-4HF3 (D-S14A-G5M2-RBD-4HF3)**<br>KVQLLETGAELVKPGASVKMSCKASGYTFTSSNIHWIKQTPGQGLEWIGAIYPGNGDTSYTQKFKGKATLTADKSSST<br>AYMQLSSLTSEDSAVYYCALDDYYALDYWGQGTTVTVSS	**>TRBD-R-4KF3 (D-S14A-G5M2-RBD-4KF3)**<br>DILMTQSPSSLAVSVGEKVAMSCKSSQSLLYSSNQKNYLAWYQQKPGQSPKLLIYWASTRESGVPDRFTGSG<br>SGTDFTLTISSVKAEDLAVYYCQQYYSYPWTFGGGTKLEIK
**>TRBD-R-4HG5 (D-S14A-G5M2-RBD-4HG5)**<br>EVKLVESGTVLARPGASVKMSCKASGYTFTSYWMHWVKQRPGRGLEWIGAIYPGNSDTNYNQKFKGKAKLTAVTST<br>STAYMDLSSLTNEDSAVYYCTRDYYGYGNYWGQGTTLTVSS	**>TRBD-R-4KG5 (D-S14A-G5M2-RBD-4KG5)**<br>KIVLTQSPASLCASVGETVTITCRASENIYSYLAWYQQKQGKSPQLLVYNAKTLAEGVPSRFSGSGSGTQFSLK<br>INSLQPEDFGSYYCQHHYGTPLTFGAGTKLEIK
**>TRBD-R-5HB9 (D-S14A-G5M2-RBD-5HB9)**<br>DVKLVESGPQLVRPGASVKISCKASGYSFTSYWMQWLKQRPGQGLEWIGMIDPSDSETRLNQKFKDKATLTVDKSSS<br>TAHMQLSSPTSEDSAVYYCARRDYYGNNPFDFWGQGTTLTVSS	**>TRBD-R-5KB9 (D-S14A-G5M2-RBD-5KB9)**<br>DIVMSQSPPSVPVTPGESVSISCRSNKSLLHSNGNTYLYWFLQRPGQSPQLLIFRMSNLASGVPDRFSGSGS<br>GTAFTLRISRVEAEDVGVYYCMQHLEYPYTFGGGTKLEIK

[a] Single-cell sorted mouse antibodies are named as TRBD-[Probe]-[Antibody index].TRBD stands for trimeric RBD. For this mouse, RBD-Avi-Biot was used as a sorting probe.

fig. S3

D

Nucleotide and amino acid sequences of monoclonal neutralizing antibodies isolated from M4 in the S2GΔHR2-5GS-1TD0 group [a].

Heavy chain	Light chain
**>S2GD-S-1HF3 (D-S14A-G7M4-1HF3)**<br>GAGGTGCAGCTGCAGCAGTCTGGGGCTGAACTGGCAAAACCTGGGGCCTCAGTGAAGATGTCCTGCAAGGCTTC<br>TGGCTACACCTTTACTAGCTACTGGGTGCACTGGGTAAAACAGAGGCCTGGACAGGGTCTGGAATGGATTGGATA<br>CATTTATCCTAGCACTGGTTATACTGACTACAATCAGAAGTTCAAGGACAAGGCCACATTGACTGCAGACAAATCC<br>TCCACCACAGCCTACATGCAACTGAGCAGCCTGACATCTGACCACTCTGCAGTCTATTACTGTGCAAGATCCGAG<br>TATGGTAACCTTTACTATGCTATGGACTACTGGGGTCAAGGAACCTCAGTCACCGTCTCCTCA	**>S2GD-S-1KF3 (D-S14A-G7M4-1KF3)**<br>CAAATTGTTCTCTCCCAGTCTCCAGCTTCTTTGGCTGTGTCTCTAGGGCAGAGGGCCACCATCTCCTGCAA<br>GGCCAGCCAAAGTGTTGATTATGATGGTGATAGTTATATGAACTGGTACCAACAGAAACCAGGACAGCCA<br>CCCAAACTCCTCATCTATTCTGCATCCAATCTAGAATCTGGGATCCCAGCCAGGTTTAGTGGCAGTGGGCC<br>TGGGACAGACTTCACCCTCAACATCCATCCTGTGGAGGAGGAGGATGCTGCAACCTATTACTGTCAACAA<br>AGTAATGAGGATCCTCTCACGTTCGGTGCTGGGACCAAGCTGGAAATAAAA
**>S2GD-S-2HC10 (D-S14A-G7M4-2HC10)**<br>CAGCGTGAGCTGCAGCAGTCTGGACCTGAGCTGGTAAAGCCTGGGGCTTCAGTGAAGATGTCCTGCAAGGCTTC<br>TGGATACACATTCACTAACTATGTTATGCACTGGGTGAAGCAGAAGCCTGGGCAGGGCCTTGAGTGGATTGGCTA<br>TATTAATCCTTACAATGATGGTACTAAGTACAATGAGAAGTTCAAAGGCAAGGCCACACTGACTTTAGACAAATCC<br>TCCAGCACAGCCTACATGGAGCTCAGCAGCCTGACCTCTGAGGACTCTGCGGTCTATTACTGTGCAAGATTTACT<br>AAGGTAGAGGGCTACTGGGGCCAAGGCTCCACTCTCACAGTCTCCTCA	**>S2GD-S-2KC10 (D-S14A-G7M4-2KC10)**<br>GACATCTTGCTGACTCAGTCTCCAGCTTCTTTGGCTGTGTCTCTAGGGCAGAGGGCCACCATCTCCTGCA<br>GAGCCAGCGAAAGTGTTGATAATTATGGCATTAGTCTTATGAACTGGTTCCAACAGAAACCAGGACAGCCA<br>CCCCAACTCCTCATCTATGCTGCATCCAACCAAGGATCCGGGGTCCCTGCCAGGTTTAGTGGCAGTGGGT<br>CTGGGACAGACTTCAGCCTCAACATCCACCCTATGGAGGAGGATGATACTGCAATGTATTTCTGTCACCAA<br>AGTAAGGAGGTTCCGTGGACGTTCGGTGGAGGCACCAAGCTGGAAATCAAACGT
**>S2GD-R-1HF9 (D-S14A-G7M4-RBD-1HF9)**<br>GAAGTGATGCTGGTGGAGTCTGGGGCTGAGCTTGTGAGGCCAGGGGCCTTAGTCAAGTTGTCCTGCAAAGCTTC<br>TGGCTTCAACATTAAAGACTACTATATGCAGTGGGTGAAGCAGAGGCCTGAACAGGGCCTGGAGTGGATTGGATG<br>GATTGATCCTGAGAATGGTAATACTATATATGACCCGAAGTTCCAGGGCAAGGCCAGTATAACAGCAGACACATC<br>CTCCAACACAGCCTACCTGCAGCTCAGCAGCCTGACATCTGAGGACACTGCCGTCTATTACTGTGCCCTCTGGGA<br>GGGGAGAGCTATGGACTACTGGGGTCAAGGAACCTCAGTCACCGTCTCCTCA	**>S2GD-R-1KF9 (D-S14A-G7M4-RBD-1KF9)**<br>GACATCCAGATGACTCAGTCTCCATCCTCCCTGTCCGCCTCTCTGGGAGACAGAGTCACCATCAGTTGCA<br>GTGCAAGTCAGGGCATCAGCAATTATTTAAATTGGTATCAGCAGAAACCAGATGGAACTGTTAAACTCCTG<br>ATCTATTACACATCAAGCTTACACTCAGGAGTCCCATCAAGGTTCAGTGGCAGTGGGTCTGGGACAGATTA<br>TTCTCTCGCCATCAGCAACCTGGAACCTGAAGATATTGTCACTTACTATTGTCAGCAGTACAGTAAACTTCC<br>GTACACGTTCGGAGGGGGGACCAAGCTGGAGCTGAAA
**>S2GD-R-2HD10 (D-S14A-G7M4-RBD-2HD10)**<br>GAAGTGATGCTGGTGGAGTCTGGGGCAGAGCTTGTGAAGCCAGGGGCCTCAGTCAAGTTGTCCTGCACAGCTTC<br>TGGCTTCAACATTAAAGACACCTTTATACACTGGGTGAAACAGAGGCCTGAACAGGGCCTGGAGTGGATTGGAGG<br>GATTGATCCTGCGAATGCTGATCCTAAATATGACCCGAAGTTTCAGGGCAAGGCCACCATAACAGCAGACACATC<br>CTCCAACACAGCCTACCTGCAGTTCAGCAGCCTGACATCTGAGGACACTGCCGTCTATTACTGTGCTCGATGGGA<br>TAATGCGGCCTATTACTATGGTATGGACTACTGGGGTCAAGGCACCTCAGTCACCGTCTCCTCA	**>S2GD-R-2KD10 (D-S14A-G7M4-RBD-2KD10)**<br>GACATCCTGATGACCCAATCTCCATCTTCCATGTATGCATCTCTAGGAGAAAGAGTCACTTTCACCTGCAA<br>GGCGAGTCAGGACATTAATAGGTTTTTAAACTGGTTCCAGCAGAAACCAGGGAAATCTCCTAAGACCCTGA<br>TCTATCGTGCAAACAGATTGGTAGATGGGGTCCCATCAAGGTTCAGTGGCAGTGGATCTGGGCAAGATTA<br>TTCTCTCACCATCAGCAGTTTGGAGTATGAAGATATGGGAATTTATTATTGTCTGCAGTATGATGAGTTGTA<br>CACGTTCGGAGGGGGGACCAAGCTGGAAATAAAA
**>S2GD-R-2HE4 (D-S14A-G7M4-RBD-2HE4)**<br>GAGGTGAAGCTGGAGGAGTCTGGGGCTGAACTGGCGAAGCCTGAGGCTTCAGTGAAGTTGTCCTGCAAGGCTTC<br>TGGCTACACCTTCACCAGCTACTTTATGTACTGGGTGAAGCAGAGGCCTGGACAAGGCCTTGAGTGGATTGGACA<br>GATTAACCCTACCAATGGTGGTACTAACTTCAATGAGAAGTTCAAGAGCAAGGCCACACTGACTGTAGACAAATC<br>CTCCAGCACAGCATACATGCAACTCAGCAGCCTGACATCTGAGGACTCTGCGGTCTATTACTGTACAATATATGG<br>AAGGTACTTTGACTGCTGGGGCCAAGGCACCACTCTCACAGTCTCCTCA	**>S2GD-R-2KE4 (D-S14A-G7M4-RBD-2KE4)**<br>GACATCCAGATGAACCAGTCTCCATCCTCTATGTCTGCATCTCTGGGAGGCAGAATAACCATCACTTGCCA<br>GGCAACTCAAGACATTGTTAAGAATTTAAACTGGTATCAGCAGAAACCAGGGAAACCCCCTTCATTCCTGA<br>TCTATTATGCAACTGAACTGGCGGAAGGGGTCCCATCAAGGTTCAGTGGCAGTGGGTCTGGGTCAGACTA<br>TTCTCTGACAATCAGCAACCTGGAGTCTGAAGATTTTGCAGACTATTACTGTCTACAGTTTTACGAGTTTCC<br>GTACACGTTCGGAGGGGGGACCAAGCTGGAAATAAAA
**>S2GD-R-2HF4 (D-S14A-G7M4-RBD-2HF4)**<br>GAAGTGATGCTGGTGGAGTCTGGACCTGAGTTGGTGAAGCCTGGGGCTTCAGTGAAGATATCCTGCAAGGCCTC<br>TGGTTACTCATTTACTGGCTACTTTATGAGCTGGGTGAAGCAGAGCCGTGGAAAGAGCCTTGAGTGGATTGGACG<br>TATTAATCCTAACAATGGTGATACTTTCTACAACCAGAAGTTCAAGGGCAAGGCCACATTGACTGTAGACAAATCC<br>TCTAGCACAGCCCACATGGACTTCCTGAGCCTGACATCTGAGGACTCTGCAGTCTATTATTGTGTAAGAGAGAGG<br>CATTACTGGGGCCAAGGCACCACTCTCACAGTCTCCTCA	**>S2GD-R-2KF4 (D-S14A-G7M4-RBD-2KF4)**<br>GAAACAACTGTGACCCAGTCTCCATCCTCCATGTCTGTATCTCTGGGAGACACAGTCAGCATCACTTGCCA<br>TGCAAGTCAGGGCATTAGCAGTAATATAGGGTGGTTGCAGCAGAAACCAGGGAAATCATTTAAGGGCCTG<br>ATCTATCATGGAACCAACTTGGAAGATGGAGTTCCATCAAGGTTCAGTGGCAGTGGATCTGGAGCAGATTA<br>TTCTCTCACCATCAGCAGCCTGGAGTCTGAAGATTTTGCAGACTATTACTGTGTACAGTATGATCAGTTTCC<br>GTACACGTTCGGAGGGGGGACCAAGCTGGAGCTGAAA
**>S2GD-S-1HF3 (D-S14A-G7M4-1HF3)**<br>EVQLQQSGAELAKPGASVKMSCKASGYTFTSYWVHWVKQRPGQGLEWIGYIYPSTGYTDYNQKFKDKATLTADKSS<br>TTAYMQLSSLTSDHSAVYYCARSEYGNLYYAMDYWGQGTSVTVSS	**>S2GD-S-1KF3 (D-S14A-G7M4-1KF3)**<br>QIVLSQSPASLAVSLGQRATISCKASQSVDYDGDSYMNWYQQKPGQPPKLLIYSASNLESGIPARFSGSGPGT<br>DFTLNIHPVEEEDAATYYCQQSNEDPLTFGAGTKLEIK
**>S2GD-S-2HC10 (D-S14A-G7M4-2HC10)**<br>QRELQQSGPELVKPGASVKMSCKASGYTFTNYVMHWVKQKPGQGLEWIGYINPYNDGTKYNEKFKGKATLTLDKSS<br>STAYMELSSLTSEDSAVYYCARFTKVEGYWGQGSTLTVSS	**>S2GD-S-2KC10 (D-S14A-G7M4-2KC10)**<br>DILLTQSPASLAVSLGQRATISCRASESVDNYGISLMNWFQQKPGQPPQLLIYAASNQGSGVPARFSGSGSGT<br>DFSLNIHPMEEDDTAMYFCHQSKEVPWTFGGGTKLEIKR
**>S2GD-R-1HF9 (D-S14A-G7M4-RBD-1HF9)**<br>EVMLVESGAELVRPGALVKLSCKASGFNIKDYYMQWVKQRPEQGLEWIGWIDPENGNTIYDPKFQGKASITADTSSNT<br>AYLQLSSLTSEDTAVYYCALWEGRAMDYWGQGTSVTVSS	**>S2GD-R-1KF9 (D-S14A-G7M4-RBD-1KF9)**<br>DIQMTQSPSSLSASLGDRVTISCSASQGISNYLNWYQQKPDGTVKLLIYYTSSLHSGVPSRFSGSGSGTDYSLA<br>ISNLEPEDIVTYYCQQYSKLPYTFGGGTKLELK
**>S2GD-R-2HD10 (D-S14A-G7M4-RBD-2HD10)**<br>EVMLVESGAELVKPGASVKLSCTASGFNIKDTFIHWVKQRPEQGLEWIGGIDPANADPKYDPKFQGKATITADTSSNTA<br>YLQFSSLTSEDTAVYYCARWDNAAYYYGMDYWGQGTSVTVSS	**>S2GD-R-2KD10 (D-S14A-G7M4-RBD-2KD10)**<br>DILMTQSPSSMYASLGERVTFTCKASQDINRFLNWFQQKPGKSPKTLIYRANRLVDGVPSRFSGSGSGQDYSL<br>TISSLEYEDMGIYYCLQYDELYTFGGGTKLEIK
**>S2GD-R-2HE4 (D-S14A-G7M4-RBD-2HE4)**<br>EVKLEESGAELAKPEASVKLSCKASGYTFTSYFMYWVKQRPGQGLEWIGQINPTNGGTNFNEKFKSKATLTVDKSSST<br>AYMQLSSLTSEDSAVYYCTIYGRYFDCWGQGTTLTVSS	**>S2GD-R-2KE4 (D-S14A-G7M4-RBD-2KE4)**<br>DIQMNQSPSSMSASLGGRITITCQATQDIVKNLNWYQQKPGKPPSFLIYYATELAEGVPSRFSGSGSGSDYSLT<br>ISNLESEDFADYYCLQFYEFPYTFGGGTKLEIK
**>S2GD-R-2HF4 (D-S14A-G7M4-RBD-2HF4)**<br>EVMLVESGPELVKPGASVKISCKASGYSFTGYFMSWVKQSRGKSLEWIGRINPNNGDTFYNQKFKGKATLTVDKSSS<br>TAHMDFLSLTSEDSAVYYCVRERHYWGQGTTLTVSS	**>S2GD-R-2KF4 (D-S14A-G7M4-RBD-2KF4)**<br>ETTVTQSPSSMSVSLGDTVSITCHASQGISSNIGWLQQKPGKSFKGLIYHGTNLEDGVPSRFSGSGSGADYSL<br>TISSLESEDFADYYCVQYDQFPYTFGGGTKLELK

[a] Single-cell sorted mouse antibodies are named as S2GD-[Probe]-[Antibody index].S2GD stands for the S2GΔHR2 spike, or the full construct name S2GΔHR2-5GS-1TD0. Probe can be S, which stands for spike, or R, which stands for RBD. For this mouse, both RBD-Avi-Biot and S2GΔHR2-5GS-foldon-Avi-Biot were used as sorting probes.

fig. S3

E

Nucleotide and amino acid sequences of monoclonal neutralizing antibodies isolated from M2 in the S2GΔHR2-10GS-I3-01v9-LD7-PADRE group [a].

Heavy chain	Light chain
**>I3V9-S-1HC9 (D-S15G2M2-1HC9)**<br>CAGGTGCAGCTGCAGCAGTCTGGGGCTGAGCTTGTGAGGCCAGGGGCCTTAGTCAAGTTGTCCTGCAAAGCTTC<br>TGGCTTCAACATTAAAGACTACTATATACACTGGGTGAAACAGAGGCCTGAACAGGGCCTGGAGTGGATTGGATG<br>GATTGATCCTGAGAATGGTAATGCTATATATGACCCGAAGTTCCAGGGCAAGGCCAGTATAACAGCAGACACATC<br>CTCCAACACAGCCTACCTGCAGCTCAGCAGCCTGACATCTGAGGACACTGCCGTCTATTACTGTGCTAGGGGGG<br>ACTTTGACTACTGGGGCCAAGGCACCACTCTCACAGTCTCCTCA	**>I3V9-S-1KC9 (D-S15G2M2-1KC9-2)**<br>GATGTTGTGATGACCCAAACTCCAGCAATCATGTCTGCATCTCCAGGGGAGAAGGTCACCATGACCTGCA<br>GTGCCAGCTCAAGTGTAGGTTACATGCACTGGTACCAGCAGAAGCCAGGATCCTCCCCCAGACTCCTGAT<br>TTATGACGCATCCAACCTGGCTTCTGGAGTCCCTGTTCGTTTCAGTGGCAGTGGGTCTGGGACCTCTTACT<br>CTCTCACAATCAGCCGAATGGAGGCTGAAGATGCTGCCACTTATTACTGCCAGCAGTGGGGTACTTACCC<br>ACCCAGGACGTTCGGTGGAGGCACCAAGCTGGAAATCAAA
**>I3V9-S-2HD8 (D-S15G2M2-2HD8)**<br>CAGGTGCAGCTGCAGCAGCCTGGGGCAGAGCTTGTGAAGCCGGGGGCCTCAGTCAAGTTGTCCTGCACAGCTT<br>CTGGCTTCAACATTAAAGACACCTATATTCACTGGGTGAATCAGAGGCCTGAACAGGGCCTGGAGTGGATTGGAA<br>GGATTGATCCTGCGAATGGTAATACTAAGTATGACCCTAAATTCCAGGGCAAGGCCACAATAACAGCAGACACCT<br>CCTCCAATACCGCCTACCTGCAGCTCAGCAGCCTGACATCTGAGGACACTGCCGTCTATTACTGTGCTGGAGGAC<br>TCTATGATTACGACGGGAGCCCTTTTGCCTACTGGGGCCAAGGCACCACGATCACCGTCTCCTCA	**>I3V9-S-2KD8 (D-S15G2M2-2KD8)**<br>GATATCCAGATGACACAGACTACATCCTCCCTGTCTGCCTCTCTGGGAGACAGAGTCACCATCAGTTGCA<br>GGGCAAGTCAGGGCATTAGCACTTATTTACACTGGTATCAGCAGAAACCAGATGGAACTCTTAAACTCCTG<br>ATCTACTACACATCAAGATTACACTCAGGTGTCCCGTCAAGGTTCAGTGGCAGTGGGTCTGGAACAGATTA<br>TTCTCTCACCATTAGCAACCTGGAACAAGAGGATGTTGCCACTTACTTTTGCCAACAGGGTAATACGCTTC<br>CGTACACGTTCGGCGGGGGGACCAAGCTGGAGCTGAAA
**>I3V9-S-2HD10 (D-S15G2M2-2HD10-2)**<br>GAGGTGAAGCTGGAGGAGTCTGGGGCTGAGCTTGTGAGGCCAGGGGCCTTGGTCAAGTTGTCCTGCAAAGCTTC<br>TGGCTTCAACATTAAAGACTACTATATACACTGGGTGAAGCAGAGGCCTGAACAGGGCCTGGAGTGGATTGGATG<br>GATTGATCCTGTTTATGGTAATTCTATATGTGACCCGAAGTTTCAGGGCAAGGCCACTATAACAGCAGACACATCC<br>TCCAACACAGCCTACCTGCAGCTCAGCAGCCTGACATCTGAGGACACTGCCGTCTATTACTGTGCCATGGGGGAT<br>GGTAACTACTGGGGCCAGGGCACCACTCTCACAGTCTCCTCA	**>I3V9-S-2KD10 (D-S15G2M2-2KD10-2)**<br>GACATCAAGATGACCCAATCTCCAGCAATCATGTCTGCATCTCCAGGGGAGAAGGTCACCATGACCTGCA<br>GTGCCACCTCAAGTGTAGATTACATGCACTGGTTCCAGCAGAAGCCAGGCACTTCTCCCAAACTCTGGATT<br>TATAGCACATCCAACCTGGCTTCTGGAGTCCCTGCTCGCTTCAGTGGCAGCGGATCTGGGACCTCTTACT<br>CTCTCACAATCAGCCGAATGGAGGCTGAAGATGCTGCCACTTATTACTGCCAGCAAAGGAGTAGTTACCC<br>AATATTCACGTTCGGCTCGGGGACCAAGCTGGAGCTGAAA
**>I3V9-R-1HG3 (D-S15G2M2-RBD-1HG3)**<br>AAGTTCCAGCTGCAGCAGTCTGGGGCTGAAATGGTGAAGCCTGGGGCTTCAGTGAAGCTGTCCTGCAAGGCTTC<br>TGGCTACACCTTCACCAACTACTGGATGTACTGGATGAAGCAGAGGCCTGGGCAAGGCCTTGAGTGGATTGGAG<br>AGATTAATCCTAGGAACGGTCTTACTAACTACAATGAGAAATTCAAGACCAAGGCCACACTGACTGTAGACAAACC<br>CTCCAGCACAGCCTTCATGCAGCTCAACAGCCTGACATCTGAGGACTCTGCGGTCTATTACTGTGCAAAGGACGG<br>TAGTAGCGCCTACTGGGGCCAAGGCACCACTCTCACAGTCTCCTCA	**>I3V9-R-1KG3 (D-S15G2M2-RBD-1KG3)**<br>GATGTTGTGGTGACTCAAACTCCATCCTCCTTATCTGCCTCTCTAGGAGAAAGAGTCAGTCTCACTTGTCG<br>GGCAAGTCAGGACATTGGTAGTAGCTTAAACTGGCTTCAGCAGGAACCAGATGGAACTATTAAACGCCTG<br>ATCTACGCCACATCCATTTTAGATTCTGGTGTCCCCAAAAGGTTCAGTGGCAGTAGGTCTGGGTCAGATTA<br>TTCTCTCACCATCAGCAGCCTTGAGTCTGAAGACTTTGTAGACTATTACTGTCTACAATATGCTGGTTCTCC<br>ATTCACGTTCGGCTCGGGGACAAAGTTGGAAATAAAA
**>I3V9-R-1HG9 (D-S15G2M2-RBD-1HG9)**<br>CAGGTGCAGCTGAAGGAGTCTGGACCTGAGCTGGTGAAGCCTGGGGCTTCAGTGAAGATATCCTGCAAGACTTC<br>TGGATACACATTCACTGAATACACCATGCTCTGGGTGAAGCAGAGCCATGGAAAGAGCCTTAAGTGGATTGGAGG<br>TATTAATCCTAACAATGGTGATACTACCTACAACCAGAAGTTCAAGGGCAAGTTTATATTGACTGTAGACAAGTCCT<br>CCAGCACAGCCTACATGGAGCTCCGCAGCCTGACATCTGAGGATTCTGCAGTCTATTACTGTGCAAGAGATGGTT<br>ACCCCTATTACTATGCTATGGACTACTGGGGTCAAGGAACCTCAGTCACCGTCTCCTCA	**>I3V9-R-1KG9 (D-S15G2M2-RBD-1KG9-1)**<br>TACATCAAGATGACCCAGTCTCCAAAATTCATGTCCACATCAGTAGGAGACAGGGTCAGCGTCACCTGCA<br>AGGCCAGTCAGAATGTGGGTACTAATGTAGCCTGGTATCAAAAGAAACCAGGGCAATCTCCTAAAGCACT<br>GATTTACTCGGCATCCTACCGGTACAGTGGAGTCCCTGATCGCTTCACAGGCAGTGGATCTGGGACAGAT<br>TTCACTCTCACCATCAGCAATGTGCAGTCTGAAGACTTGGCAGAGTATTTCTGTCAACAATATAACAGCTAT<br>CCGTGGACGTTCGGTGGAGGCACCAAGCTGGAAATCAAA
**>I3V9-R-1HF5 (D-S15G2M2-RBD-1HF5)**<br>CAGGTGCAGCTGAAGGAGTCTGGACCTGAGCTGGTGAAGCCTGGGGCCTCAGTGAAGATTTCCTGCAAAGTTTC<br>TGGTTCCGCATTCAGTAATTCTTGGATGAACTGGGTGAAGCAGAGGCCTGGACAGGGTCTTGAATGGATTGGACG<br>GATTTATCTTGGAGATGGGGATACTAACTTCAATGGGAAATTCAAGGACAAGGCCACACTGACTGCAGACAAATC<br>CTCCACCACAGCCTACATGCAACTCAGCAGCCTCACCTCTGTGGACTCTGCGGTCTATTTCTGTGCAAATTCCTG<br>GGACGGTCTGGTGTTTGCTTACTGGGGCCAGGGGACTCTGGTCACTGTCTCTGCA	**>I3V9-R-1KF5 (D-S15G2M2-RBD-1KF5)**<br>AACATTGTGATGTCACAGTCTCCAACAATCATGTCTGCATCTCCAGGGGAGAAGGTCACTTTGACCTGCAG<br>TGCCAGCTCAGGTGTTACTTACATCTTCTGGTACCAGCAGAAGCCAGGATCCTCCCCCAGACTCCTGATTT<br>ATGACACATCCATCCTGGCTTCTGGAGTCCCTGTTCGCTTCAGTGGCGGTGGGTCTGGGACCTCTTACTC<br>TCTCACACTCAGCCGAATGGAGGCTGAAGATGCTGCCACTTATTACTGCCAGCAGTGGAATAATTTCCCAC<br>CCACGTTCGGTCCTGGGACCAAGCTGGAGCTGAAA
**>I3V9-R-2HE11 (D-S15G2M2-RBD-2HE11)**<br>GAGGTGCAGCTGGTGGAGTCTGGGGCTGAACTGGTGAAGCCTGGGGCTTCAGTGAAGTTGTCCTGCAAGGCTTC<br>TGGCTACAGCTTTACCACCTACTATATATACTGGATGAAGCAGAGGCCTGGACAGGGCCTTGAGTGGATTGGAGA<br>GATTAATCCTAGCAATGGTGGTACTAACTTCAATGAGAGGTTCAAGAGCAAGGCCACACTGACTGTAGACAAATC<br>CTCCAGCACAGCATACATGCAACTCAGCAGCCTGACATCTGAGGACTCTGCGGTCTATTACTGTTCAAGAGATGG<br>TAGTATTGCTTACTGGGGCCAAGGGACTCTGGTCACTGTCTCTGCA	**>I3V9-R-2KE11 (D-S15G2M2-RBD-2KE11-4)**<br>GAAAATGTTCTCACCCAGTCTCCATCCTCCTTATCTGCCTCTCTGGGAGAAAGAGTCAGTCTCACTTGTCG<br>GGCAAGTCAGGACACTGGTAGTAGTTTAAACTGGCTTCAGCAGGAACCAGATGGAACTATTAAACGCCTG<br>ATCTACGCCACATCCAATTTAGATTCTGGTGTCCCCAAAAGGTTCAGTGGCAGTAGGTCTGGGTCAGATTA<br>TTCTCTCACCATCAGCAGCCTTGAGTCTGAAGATTTTGTAGACTATTACTGTCTACAATATGCTAGTTCTCC<br>GTACACGTTCGGAGGGGGGACCAAGCTGGAAATAAAA
**>I3V9-R-2HF2 (D-S15G2M2-RBD-2HF2)**<br>GAGGTGAAGCTGGAGGAGTCTGGACCTGAGCTGGTGAAGCCTGGGGCTTCAGTGAAGATTTCCTGCAAGACTTC<br>TGGATTCACATTCACTGAATACACCATGCACTGGGTGAAGCAGAGCCATGGAAAGAGCCTTGAGTGGATTGGAGC<br>TATTAATCTTAACATTGTTGATACCATCTACAATCAGAACTTCCAGGGCAAGGCCACATTTGCTGTAGACAAGTCCT<br>CCAGCACAGCCTACATGGAACTCCGTAGCCTGACATCTGAAGATTCTTCAGTCTACTACTGCGCAAGGGATAGGT<br>ACGACCGGTACTTCGATGTCTGGGGCGCAGGGACCACGGTCACCGTCTCCTCA	**>I3V9-R-2KF2 (D-S15G2M2-RBD-2KF2)**<br>TACATTGTGATGACTCAGTCTCCAACGATCATGTCTGCGTCTCCAGGGGAGAAGGTCACTTTGACCTGCAG<br>TGCCAGCTCAGGTGTTACTTACATCTTCTGGTACCAGCAGAAGCCAGGATCCTCCCCCAGACTCCTGATTT<br>ATGACACATCCATCCTGGCTTCTGGAGTCCCTGTTCGCTTCAGTGGCGGTGGGTCTGGGACCTCTTACTC<br>TCTCACACTCAGCCGAATGGAGGCTGAAGATGCTGCCACTTATTACTGCCAGCAGTGGAATAATTTCCCAC<br>CCACGTTCGGTCCTGGGACCAAGCTGGAAATAAAA
**>I3V9-R-2HF5 (D-S15G2M2-RBD-2HF5)**<br>CAGGCTTATCTACAGCAGTCTGGACCTGAGCTGGTGAAGCCTGGGGCTTCAGTGAAGATATCCTGCAAGGCTTCT<br>GGTTACTCTTTTACTGAATACTTTATGAACTGGGTGATGCAGAGCCATGGAAAGAGCCTTGAGTGGATTGGACGTA<br>TTAATCCTTACAATGGTGATACTTTCTACAACCAGAAGTTCAAGGGCAAGGCCACATTGACTGTAGACAAATCATC<br>TAGCACTGCCCACATGGAGTTCCGGAGCCTGGCATCTGAGGACTCCGCAGTCTATTATTGTGCAAGATCCCATGA<br>TTACCCCTTTGACTACTGGGGCCAAGGCACCACTCTCACAGTCTCCTCA	**>I3V9-R-2KF5 (D-S15G2M2-RBD-2KF5)**<br>GACATCCTGATGACCCAATCTCCATCGTCCATGTTTGCCTCTCTGGGAGACAGAGTCAGTCTCTCTTGTCG<br>GGCTAGTCAGGGCATTAGAGGTAATTTAGACTGGTATCAGCAGAAACCAGGTGGAACTATTAAACTCCTGA<br>TCTACTCCACATTTAATTTAAATTTTGGTGTCCCATCAAGGTTCAGTGGCAGTGGGTCTGGGTCAGATTATT<br>CTCTCACCATCAGCAGCCTAGAGTCTGAAGATTTTGCAGACTATTACTGTCTACAGCGTAATGCGTATCCG<br>TACACGTTCGGAGGGGGGACAAAGTTGGAAATAAAA
**>I3V9-R-2HF10 (D-S15G2M2-RBD-2HF10)**<br>GAGTTCCAGCTGCAGCAGTCTGGACCTGAACTGGTGAGGCCTGGGGCTTCAGTGAAGATATCCTGCAGGACTTC<br>TGGATACACATTCACTGAATACACCCTGTACTGGGTGAAGCAGAGCCATGGAAAGAGCCTTGAGTGGATTGGAGG<br>TATTAATCCTAACAATGGTGATACTACCTACAACCAGAGGTTTAAGGGCAGGGCCACATTGACTGTAGACAAGTCA<br>TCCAGCACAGCCTACATGGAGCTCCGCAGCCTGACATCTGAGGATTCTGCAGTCTATTACTGTGCGAGAGATGGT<br>TACCCCTATTACTATGCCTTGGACTATTGGGGTCAAGGAACCACTCTCACAGTCTCCTCA	**>I3V9-R-2KF10 (D-S15G2M2-RBD-2KF10-2)**<br>TACATCTTGCTGACTCAGTCTCCAGCCTCTCAGTCTGCATCTCTGGGAGAAAGTGTCACCATCACATGCCT<br>GGCAAGTCAGACCATTGGTACATGGTTAGCATGGTATCAGCAGAAACCAGGGAAATCTCCTCAGCTCCTG<br>ATTTATGCTGCAACCAGCTTGGCAGATGGGGTCCCATCAAGGTTCAGTGGTAGTGGATCTGGCACAAAATT<br>TTCTTTCAAGATCAGCAGCCTACAGGCTGAAGATTTTGTAAGTTATTACTGTCAACAACTTTACAGTACTCC<br>TCTGACGTTCGGTGGAGGCACCAAGCTGGAAATCAAA
**>I3V9-S-1HC9 (D-S15G2M2-1HC9)**<br>QVQLQQSGAELVRPGALVKLSCKASGFNIKDYYIHWVKQRPEQGLEWIGWIDPENGNAIYDPKFQGKASITADTSSNT<br>AYLQLSSLTSEDTAVYYCARGDFDYWGQGTTLTVSS	**>I3V9-S-1KC9 (D-S15G2M2-1KC9-2)**<br>DVVMTQTPAIMSASPGEKVTMTCSASSSVGYMHWYQQKPGSSPRLLIYDASNLASGVPVRFSGSGSGTSYSL<br>TISRMEAEDAATYYCQQWGTYPPRTFGGGTKLEIK
**>I3V9-S-2HD8 (D-S15G2M2-2HD8)**<br>QVQLQQPGAELVKPGASVKLSCTASGFNIKDTYIHWVNQRPEQGLEWIGRIDPANGNTKYDPKFQGKATITADTSSNT<br>AYLQLSSLTSEDTAVYYCAGGLYDYDGSPFAYWGQGTTITVSS	**>I3V9-S-2KD8 (D-S15G2M2-2KD8)**<br>DIQMTQTTSSLSASLGDRVTISCRASQGISTYLHWYQQKPDGTLKLLIYYTSRLHSGVPSRFSGSGSGTDYSLTI<br>SNLEQEDVATYFCQQGNTLPYTFGGGTKLELK
**>I3V9-S-2HD10 (D-S15G2M2-2HD10-2)**<br>EVKLEESGAELVRPGALVKLSCKASGFNIKDYYIHWVKQRPEQGLEWIGWIDPVYGNSICDPKFQGKATITADTSSNTA<br>YLQLSSLTSEDTAVYYCAMGDGNYWGQGTTLTVSS	**>I3V9-S-2KD10 (D-S15G2M2-2KD10-2)**<br>DIKMTQSPAIMSASPGEKVTMTCSATSSVDYMHWFQQKPGTSPKLWIYSTSNLASGVPARFSGSGSGTSYSL<br>TISRMEAEDAATYYCQQRSSYPIFTFGSGTKLELK
**>I3V9-R-1HG3 (D-S15G2M2-RBD-1HG3)**<br>KFQLQQSGAEMVKPGASVKLSCKASGYTFTNYWMYWMKQRPGQGLEWIGEINPRNGLTNYNEKFKTKATLTVDKPS<br>STAFMQLNSLTSEDSAVYYCAKDGSSAYWGQGTTLTVSS	**>I3V9-R-1KG3 (D-S15G2M2-RBD-1KG3)**<br>DVVVTQTPSSLSASLGERVSLTCRASQDIGSSLNWLQQEPDGTIKRLIYATSILDSGVPKRFSGSRSGSDYSLTI<br>SSLESEDFVDYYCLQYAGSPFTFGSGTKLEIK
**>I3V9-R-1HG9 (D-S15G2M2-RBD-1HG9)**<br>QVQLKESGPELVKPGASVKISCKTSGYTFTEYTMLWVKQSHGKSLKWIGGINPNNGDTTYNQKFKGKFILTVDKSSST<br>AYMELRSLTSEDSAVYYCARDGYPYYYAMDYWGQGTSVTVSS	**>I3V9-R-1KG9 (D-S15G2M2-RBD-1KG9-1)**<br>YIKMTQSPKFMSTSVGDRVSVTCKASQNVGTNVAWYQKKPGQSPKALIYSASYRYSGVPDRFTGSGSGTDFT<br>LTISNVQSEDLAEYFCQQYNSYPWTFGGGTKLEIK
**>I3V9-R-1HF5 (D-S15G2M2-RBD-1HF5)**<br>QVQLKESGPELVKPGASVKISCKVSGSAFSNSWMNWVKQRPGQGLEWIGRIYLGDGDTNFNGKFKDKATLTADKSST<br>TAYMQLSSLTSVDSAVYFCANSWDGLVFAYWGQGTLVTVSA	**>I3V9-R-1KF5 (D-S15G2M2-RBD-1KF5)**<br>NIVMSQSPTIMSASPGEKVTLTCSASSGVTYIFWYQQKPGSSPRLLIYDTSILASGVPVRFSGGGSGTSYSLTLS<br>RMEAEDAATYYCQQWNNFPPTFGPGTKLELK
**>I3V9-R-2HE11 (D-S15G2M2-RBD-2HE11)**<br>EVQLVESGAELVKPGASVKLSCKASGYSFTTYYIYWMKQRPGQGLEWIGEINPSNGGTNFNERFKSKATLTVDKSSST<br>AYMQLSSLTSEDSAVYYCSRDGSIAYWGQGTLVTVSA	**>I3V9-R-2KE11 (D-S15G2M2-RBD-2KE11-4)**<br>ENVLTQSPSSLSASLGERVSLTCRASQDTGSSLNWLQQEPDGTIKRLIYATSNLDSGVPKRFSGSRSGSDYSL<br>TISSLESEDFVDYYCLQYASSPYTFGGGTKLEIK
**>I3V9-R-2HF2 (D-S15G2M2-RBD-2HF2)**<br>EVKLEESGPELVKPGASVKISCKTSGFTFTEYTMHWVKQSHGKSLEWIGAINLNIVDTIYNQNFQGKATFAVDKSSSTA<br>YMELRSLTSEDSSVYYCARDRYDRYFDVWGAGTTVTVSS	**>I3V9-R-2KF2 (D-S15G2M2-RBD-2KF2)**<br>YIVMTQSPTIMSASPGEKVTLTCSASSGVTYIFWYQQKPGSSPRLLIYDTSILASGVPVRFSGGGSGTSYSLTLS<br>RMEAEDAATYYCQQWNNFPPTFGPGTKLEIK
**>I3V9-R-2HF5 (D-S15G2M2-RBD-2HF5)**<br>QAYLQQSGPELVKPGASVKISCKASGYSFTEYFMNWVMQSHGKSLEWIGRINPYNGDTFYNQKFKGKATLTVDKSSS<br>TAHMEFRSLASEDSAVYYCARSHDYPFDYWGQGTTLTVSS	**>I3V9-R-2KF5 (D-S15G2M2-RBD-2KF5)**<br>DILMTQSPSSMFASLGDRVSLSCRASQGIRGNLDWYQQKPGGTIKLLIYSTFNLNFGVPSRFSGSGSGSDYSL<br>TISSLESEDFADYYCLQRNAYPYTFGGGTKLEIK
**>I3V9-R-2HF10 (D-S15G2M2-RBD-2HF10)**<br>EFQLQQSGPELVRPGASVKISCRTSGYTFTEYTLYWVKQSHGKSLEWIGGINPNNGDTTYNQRFKGRATLTVDKSSST<br>AYMELRSLTSEDSAVYYCARDGYPYYYALDYWGQGTTLTVSS	**>I3V9-R-2KF10 (D-S15G2M2-RBD-2KF10-2)**<br>YILLTQSPASQSASLGESVTITCLASQTIGTWLAWYQQKPGKSPQLLIYAATSLADGVPSRFSGSGSGTKFSFKI<br>SSLQAEDFVSYYCQQLYSTPLTFGGGTKLEIK

[a] Single-cell sorted mouse antibodies are named as I3V9-[Probe]-[Antibody index].I3V9 stands for the I3-01v9 nanoparticle, or the full construct name S2GΔHR2-10GS-I3-01v9-LD7-PADRE. Probe can be S, which stands for spike, or R, which stands for RBD. For this mouse, both RBD-Avi-Biot and S2GΔHR2-5GS-foldon-Avi-Biot were used as sorting probes.

fig. S3

F Mouse antibody neutralization against SARS-CoV-2 strains Wuhan-Hu-1, B.1.1.7, B1.351, P.1 and B.1.617$_{Rec}$

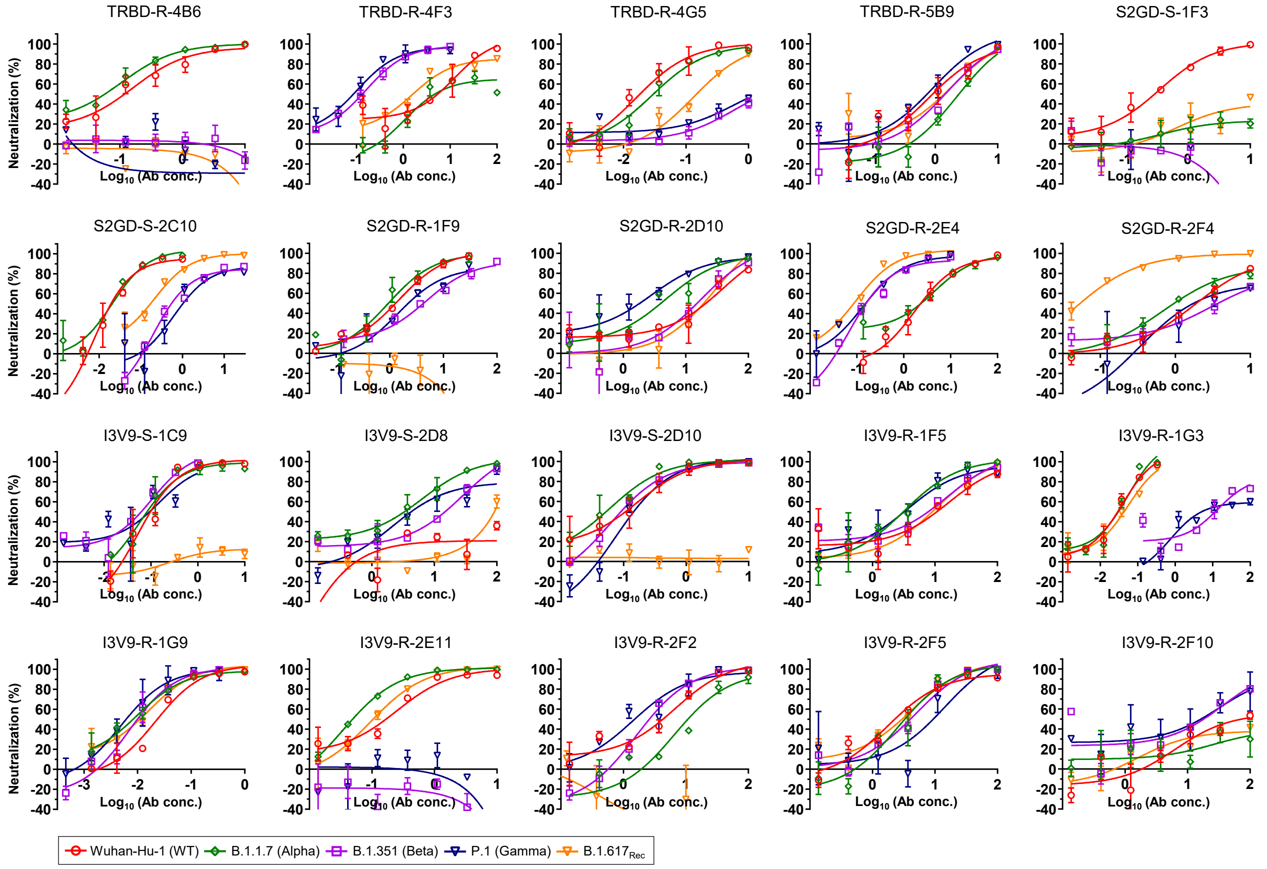

G Mouse antibodies isolated from mice in three vaccine groups binding to RBD and spike antigens

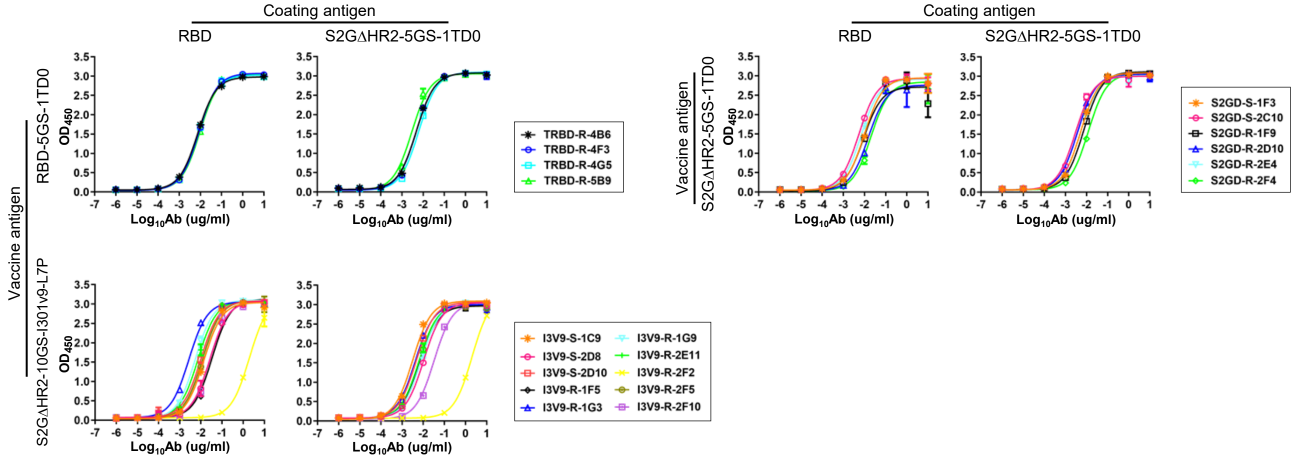

H

EC_{50} values (μg/ml) of 20 mouse antibodies isolated from mice in three vaccine groups binding to SARS-CoV-2 RBD and spike antigens [a].

	TRBD-R-4B6	TRBD-R-4F3	TRBD-R-4G5	TRBD-R-5B9	S2GD-S-1F3	S2GD-S-2C10	S2GD-R-1F9	S2GD-R-2D10	S2GD-R-2E4	S2GD-R-2F4
RBD	0.007	0.009	0.008	0.010	0.010	0.005	0.009	0.015	0.007	0.020
S2GΔHR2-5GS-1TD0 spike	0.005	0.005	0.006	0.003	0.005	0.003	0.007	0.004	0.005	0.012

	I3V9-S-1C9	I3V9-S-2D8	I3V9-S-2D10	I3V9-R-1G3	I3V9-R-1G9	I3V9-R-1F5	I3V9-R-2E11	I3V9-R-2F2	I3V9-R-2F5	I3V9-R-2F10
RBD	0.014	0.027	0.010	0.003	0.005	0.034	0.007	1.973	0.012	0.026
S2GΔHR2-5GS-1TD0 spike	0.003	0.010	0.005	0.004	0.008	0.007	0.006	2.051	0.006	0.032

[a] EC_{50} values were calculated from the besting fitting in GraphPad Prism v9.1.2.

fig. S3. Single-cell isolation and functional evaluation of monoclonal neutralizing antibodies from mice immunized with the RBD, spike, and SApNP vaccines. (**A**) SEC profiles of biotinylated Avi-tagged SARS-CoV-2 spike (S2GΔHR2-5GS-foldon-Avi-Biot) and RBD (RBD-5GS-foldon-Avi-Biot) probes from a Superdex 200 Increase 10/300 GL column and a HiLoad Superose 6 16/600 column, respectively. Foldon (PDB: 1RFO) is a trimerization motif used to stabilize the spike and RBD in trimeric conformations and to mask 1TD0-specific B cells (1TD0 is a trimerization motif used in the RBD and spike vaccine constructs). (**B**) Gating strategies used in the antigen-specific single-cell sorting of mouse splenic B cells (Step 1: remove cell debris; Steps 2 and 3: exclude clumped or sticky cells to ensure that only single cells remain; Step 4: remove dead cells; Step 5: identify antigen-specific B cells). Spleen samples from M2 in the RBD-5GS-1TD0 group, M4 in the S2GΔHR2-5GS-1TD0 group, and M2 in the S2GΔHR2-10GS-I3-01v9-L7P group were single-cell sorted using the probes in (A). While only the RBD probe was used to sort B cells from M2 in the RBD-5GS-1TD0 group, both probes were used to sort B cells from the two mice immunized with spike-based vaccines, resulting in a total of 5 sorting experiments. In each sorting experiment, a 96-well plate was used to collect single B cells, which were subjected to antibody cloning and functional validation. Nucleotide and amino acid sequences of monoclonal neutralizing antibodies isolated from (**C**) M2 in the RBD-10GS-1TD0 group (4 antibodies), (**D**) M4 in the S2GΔHR2-5GS-1TD0 group (6 antibodies), and (**E**) M2 in the S2GΔHR2-10GS-I3-01v9-L7P (10 antibodies). Antibodies are named as [Vaccine]-[Probe]-[Antibody index], with heavy and κ-light chains indicated by “H” and “K”, respectively. Vaccine can be TRBD, which stands for the RBD-5GS-1TD0 trimer, S2GD, which stands for the S2GΔHR2-5GS-1TD0 spike, and I3V9, which stands for the S2GΔHR2-10GS-I3-01v9-L7P NP. Probe can be S, which stands for spike, and R, which stands for RBD. (**F**) Neutralization curves of 20 mouse monoclonal neutralizing antibodies against SARS-CoV-2-pps that carry spikes of four strains, the wildtype Wuhan-Hu-1 strain and four variants, B.1.1.7, B.1.351, P.1, and $B.1.617_{Rec}$. IC_{50} values were calculated in GraphPad Prism 9.1.2 and are summarized in Fig. 3B. (**G**) ELISA curves of 20 mouse monoclonal neutralizing antibodies binding to the RBD and spike antigens derived from the wildtype Wuhan-Hu-1 strain. All ELISA binding assays were performed in duplicates. (**H**) Summary of EC_{50} values (μg/ml) measured for antibody-antigen binding in (G).

fig. S4

A

Gating strategies in bulk sorting of mouse splenic B cells

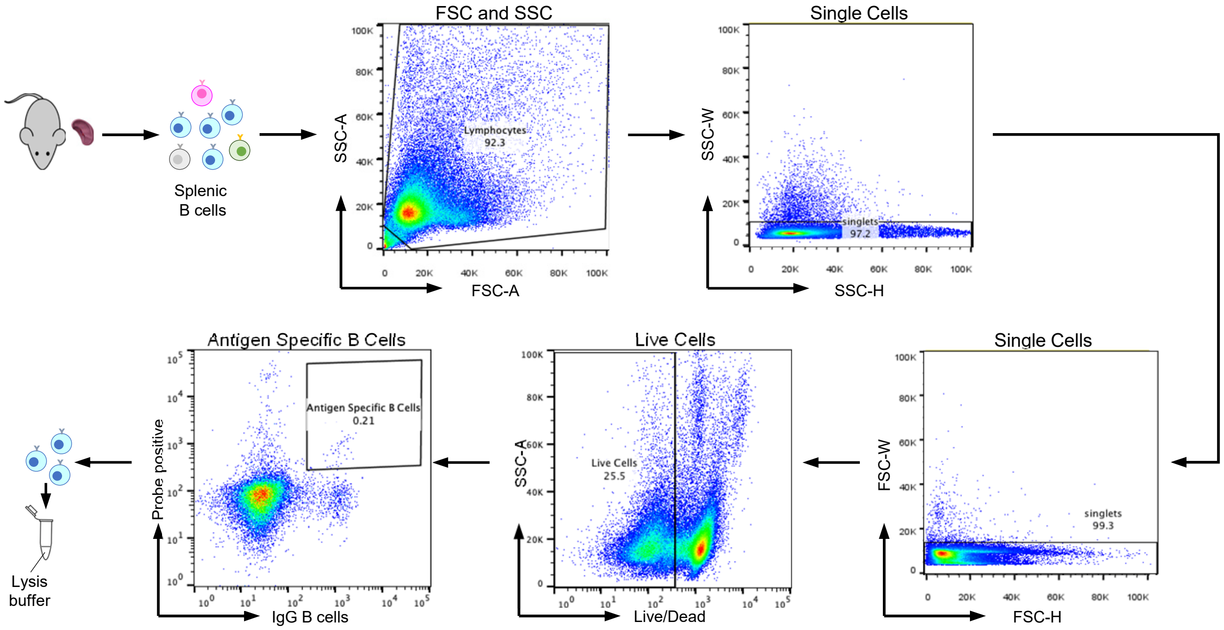

Antigen-specific sorting of mouse splenic B cells from 3 mice [a]

	M2 in the RBD-5GS-1TD0 group		M4 in the S2GΔHR2-5GS-1TD0 group		M2 in the S2GΔHR2-10GS-I3-01v9-L7P group	
Probe	Sorted B cells	%Splenic B cells	Sorted B cells	%Splenic B cells	Sorted B cells	%Splenic B cells
S2GΔHR2-5GS-foldon-Avi-Biot	1481	0.36370	1550	0.21626	1407	0.25887
RBD-5GS-foldon-Avi-Biot	1565	0.23684	1550	0.27332	1402	0.37566

[a] Spleen samples from three mice in the previous study (Ref 41) were processed for bulk sorting of antigen-specific B cells to facilitate deep sequencing analysis. Two SARS-CoV-2 antigen probes, S2GΔHR2-5GS-foldon-Avi-Biot and RBD-Avi-Biot, were used in the bulk sorting.

B

Next-generation sequencing (NGS) analysis of 3 mice immunized with RBD, spike, and nanoparticle vaccines [a]

Vaccine antigen	Probe [b]	N_{Raw}	$N_{V\text{-}assign}$	$N_{V\text{-}align\ (250bp)}$	Chain	N_{Chain}	N_{Step5}	<Length>	N_{Usable}
M2 in the RBD-5GS-1TD0 group	Spike	2,067,177	842,447	335,620	H	150,323	149,768	361.0	149,767
					K	185,297	184,846	331.5	184,845
	RBD	1,258,194	831,049	470,912	H	208,287	207,731	359.9	207,728
					K	262,625	262,030	335.5	262,028
M4 in the S2GΔHR2-5GS-1TD0 group	Spike	1,431,024	771,147	376,522	H	111,699	111,440	357.7	111,438
					K	264,823	264,345	339.6	264,344
	RBD	1,380,740	907,658	380,881	H	154,863	154,571	355.4	154,570
					K	226,017	224,892	337.9	224,892
M2 in the S2GΔHR2-10GS-I3-01v9-L7P group	Spike	3,952,238	1,873,397	720,897	H	411,322	410,074	360.2	410,068
					K	309,575	308,842	330.3	308,842
	RBD	1,752,190	954,410	389,625	H	200,507	197,000	361.0	196,995
					K	189,118	188,800	329.9	188,798

[a] Listed items include the vaccine antigen, mouse sample ID, number of raw reads from Ion S5 sequencing, number of reads that can be assigned to a VH/VK gene with an E-value of 10^{-3} or lower, number of reads that can be aligned to a VH/VK gene with 250bp or longer, number of VH/VK chains, number of VH/VK chains at the last step (5) of pipeline processing, average read length, and number of usable chains. Of note, to determine usable chains, the 250bp V-gene alignment filter was applied again to remove any problematic sequences detected during the full pipeline processing.

[b] Two SARS-CoV-2 probes were used in antigen-specific bulk sorting of mouse splenic B cells. Spike stands for S2GΔHR2-5GS-foldon-Avi-Biot; RBD stands for RBD-5GS-foldon-Avi-Biot.

fig. S4

C **Spike/RBD-specific bulk sorting of splenic B cells from three mice**

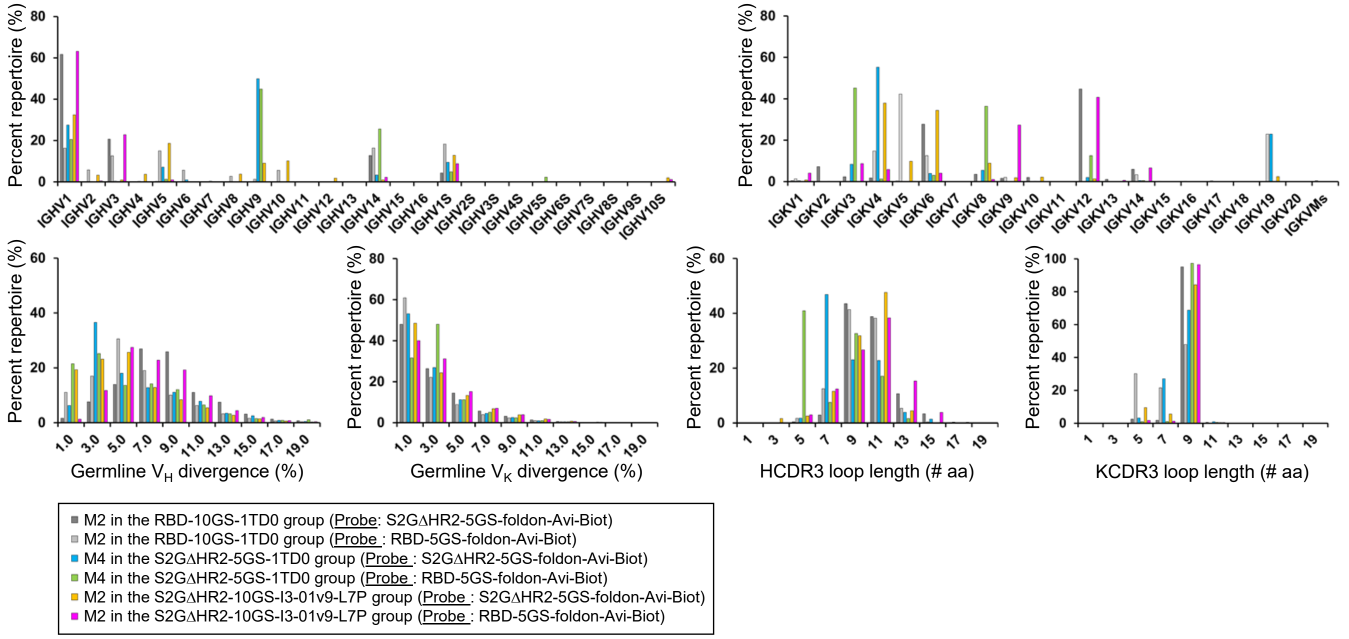

D **Tracing RBD-5GS-1TD0 trimer-elicited NAbs in spike-sorted B cell populations**

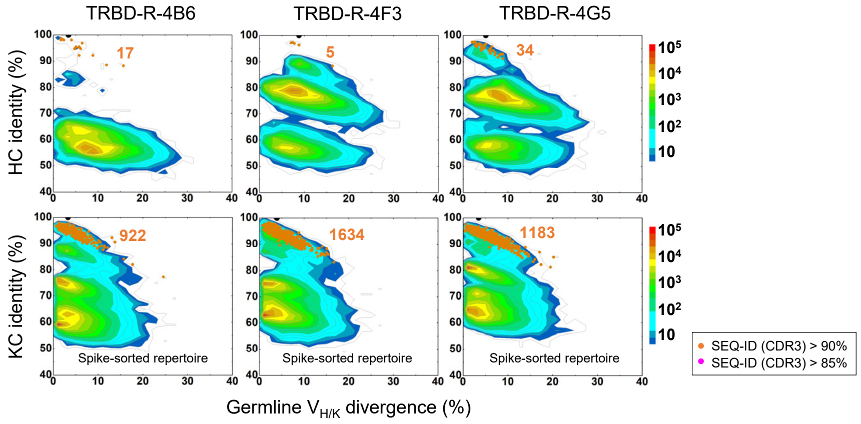

E **Tracing S2GΔHR2-5GS-1TD0 spike-elicited NAbs in spike/RBD-sorted B cell populations**

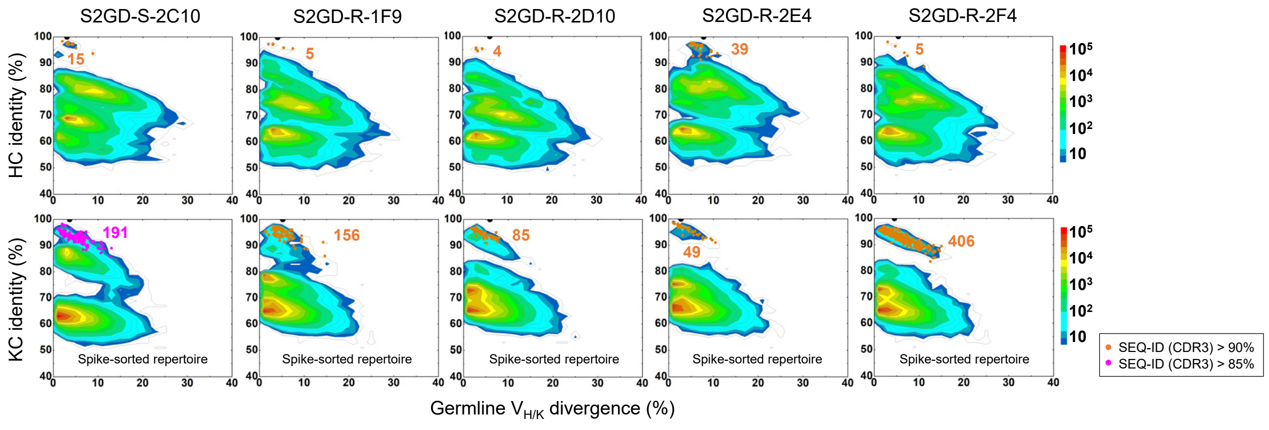

fig. S4

E **(continued)**

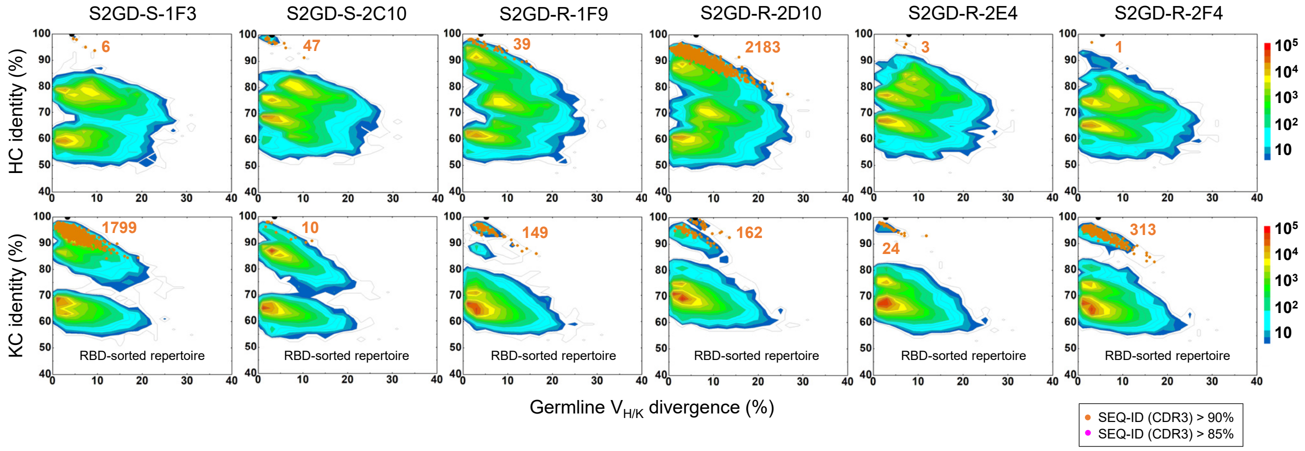

F **Tracing S2GΔHR2-10GS-I3-01v9-L7P NP-elicited NAbs in spike/RBD-sorted B cell populations**

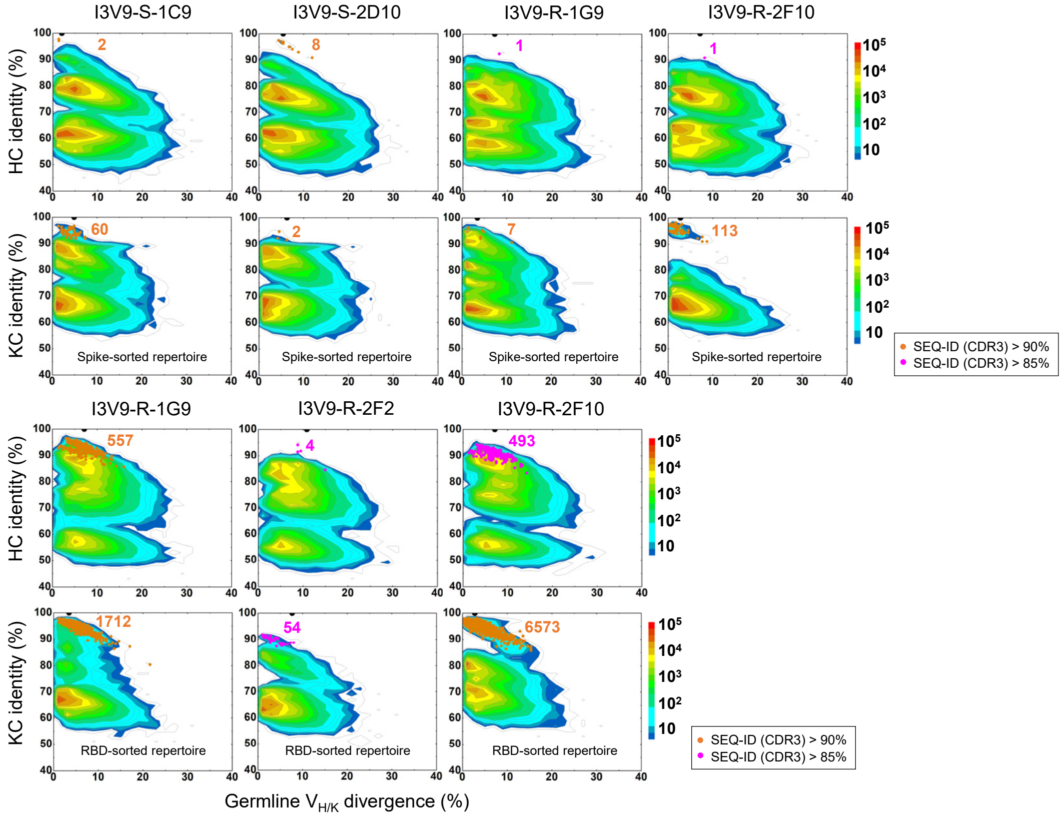

fig. S4

fig. S4. Unbiased repertoire analysis of bulk-sorted SARS-CoV-2 antigen-specific mouse splenic B cells and tracing of mouse neutralizing antibodies in the NGS-derived repertoires. Two SARS-CoV-2 antigen probes in fig. S3A were used to sort mouse splenic B cells. (**A**) Bulk B-cell sorting experiment. Top: gating strategies used in the antigen-specific sorting of mouse splenic B cells (Step 1: remove cell debris; Steps 2 and 3: exclude clumped or sticky cells to ensure that only single cells remain; Step 4: remove dead cells; Step 5: identify antigen-specific B cells). Bottom: Summary of SARS-CoV-2 antigen-specific bulk sorting of mouse splenic B cells from three mice. (**B**) Antibodyomics pipeline analysis of repertoire NGS data obtained for spike and RBD-specific mouse splenic B cells from three mice. (**C**) B-cell repertoire profiles are shown for three mice immunized with RBD-5GS-1TD0, S2GΔHR2-5GS-1TD0, and S2GΔHR2-10GS-I3-01v9-L7P. Top: VH gene usage (left) and VK gene usage (right); Bottom: germline VH/VK divergence (left) and CDRH/K3 loop length (right). **(D)** Divergence-identity analysis of NAbs in the context of RBD-sorted B-cell repertoires for M2 in the RBD-5GS-1TD0 group. (**E**) Divergence-identity analysis of NAbs in the context of spike and RBD-sorted B-cell repertoires for M4 in the S2GΔHR2-5GS-1TD0 group. (**F**) Divergence-identity analysis of NAbs in the context of spike and RBD-sorted B-cell repertoires for M2 in the S2GΔHR2-10GS-I3-01v9-L7P group. HC and KC sequences are plotted as a function of sequence identity to a given template NAb and sequence divergence from putative germline V genes. Color coding indicates sequence density. On the 2D plots, template NAbs are shown as black dots, whereas somatic variants that were identified based on the germline V gene and the CDR3 identity of 85/90% or greater are shown as orange/magenta dots, with the number of sequences labeled accordingly. In (D)-(F), the 2D plots are only shown for NAbs for which both HC and KC somatic variants could be found in the NGS repertoires.

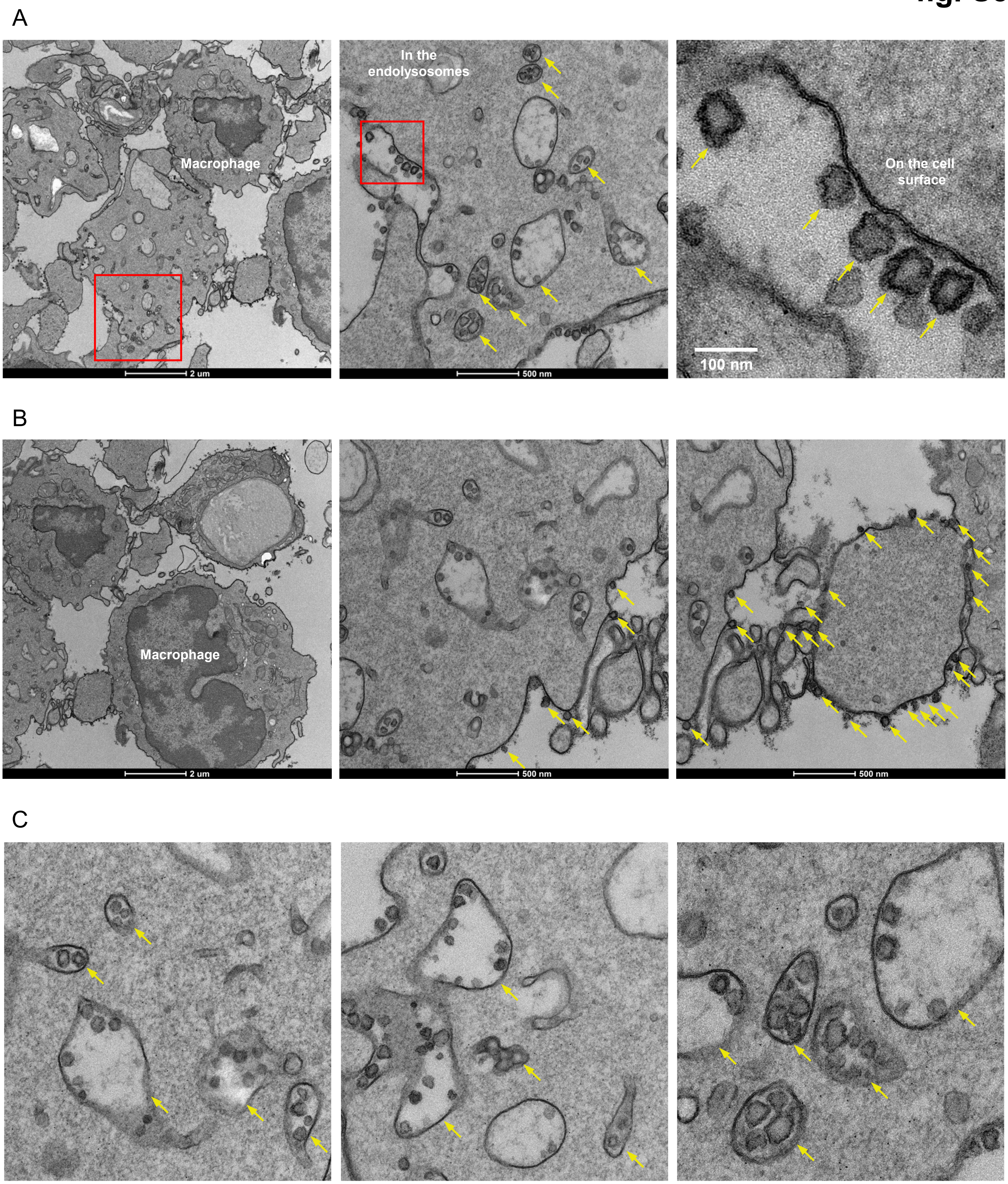

fig. S5. SARS-CoV-2 spike-presenting I3-01v9 SApNP interaction with macrophages in a lymph node. (**A**) S2GΔHR2-presenting I3-01v9 SApNPs are sequestered by macrophages in the medullary cord zone of a lymph node after a single-dose injection (50 μg). S2GΔHR2-presenting I3-01v9 SApNPs (**B**) aligned on macrophage surface or (**C**) sequestered inside the endolysosomes of a macrophage. S2GΔHR2-presenting I3-01v9 SApNPs are pointed by yellow arrows.

fig. S6

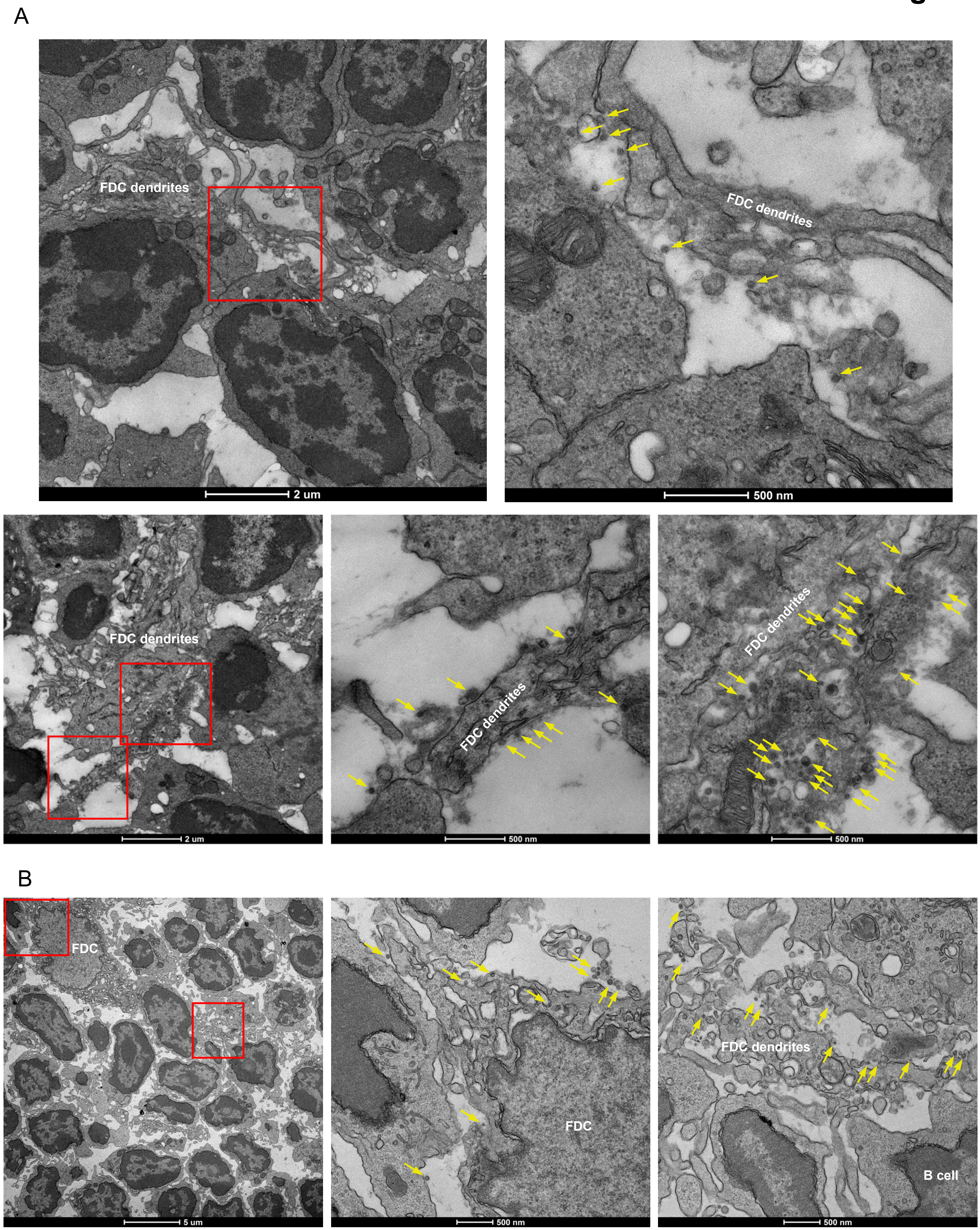
A
FDC dendrites
2 um
FDC dendrites
500 nm
FDC dendrites
2 um
FDC dendrites
500 nm
FDC dendrites
500 nm
B
FDC
5 um
FDC
500 nm
FDC dendrites
B cell
500 nm

fig. S6

C

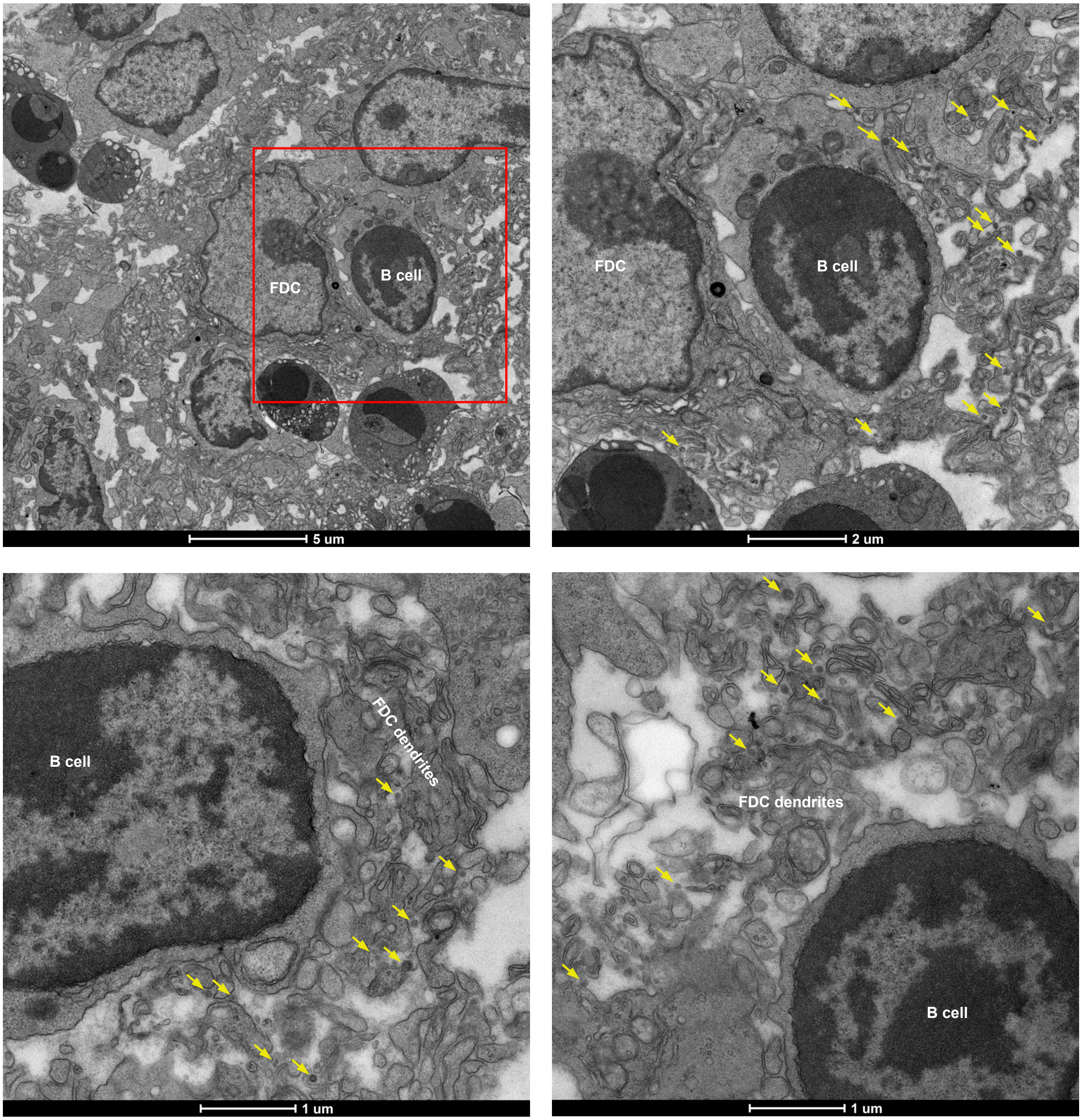
FDC
B cell
5 um
FDC
B cell
2 um
B cell
FDC dendrites
1 um
FDC dendrites
B cell
1 um

fig. S6

D

I3-01 nanoparticles injected at 2h

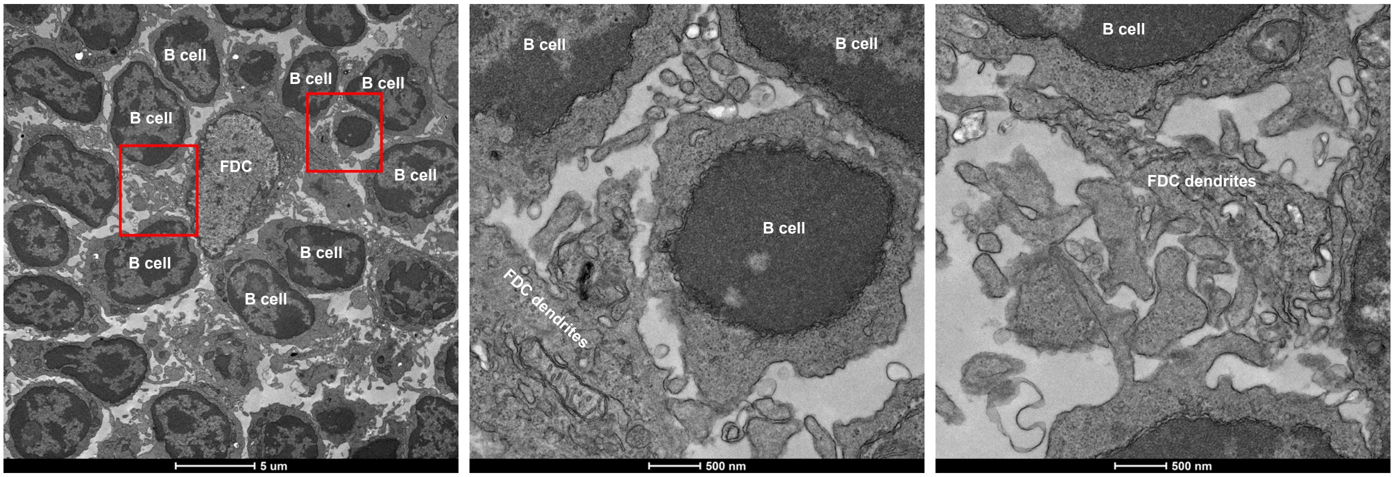

E

Lymph node from naïve mouse

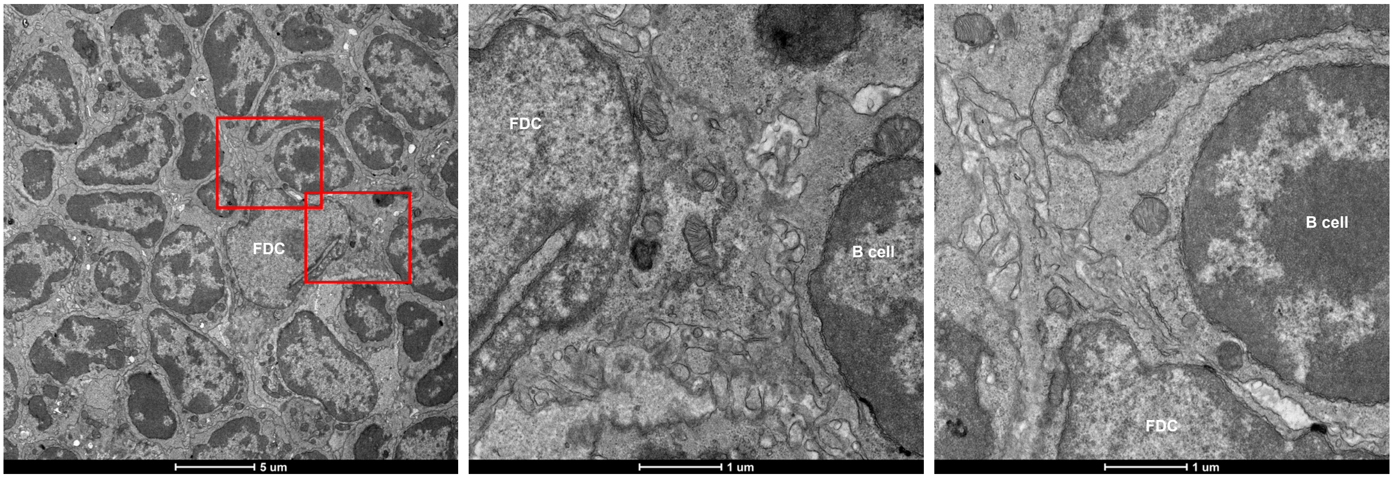

fig. S6. TEM images of SARS-CoV-2 spike-presenting I3-01v9 SApNP interaction with FDCs in a lymph node. S2GΔHR2-presenting I3-01v9 SApNPs are aligned on FDC dendrites (**A**) at 12 h after a single-dose injection (50 μg), (**B**) at 48 h after a single-dose injection (50 μg), and (**C**) at 12 h after the boost injection (50 μg). S2GΔHR2-presenting I3-01v9 SApNPs are pointed by yellow arrows. (**D**) S2GΔHR2-presenting I3-01v9 SApNPs were barely observed at 2 h after a single-dose injection (50 μg). (**E**) Lymph node from a naïve, unimmunized mouse.

fig. S7

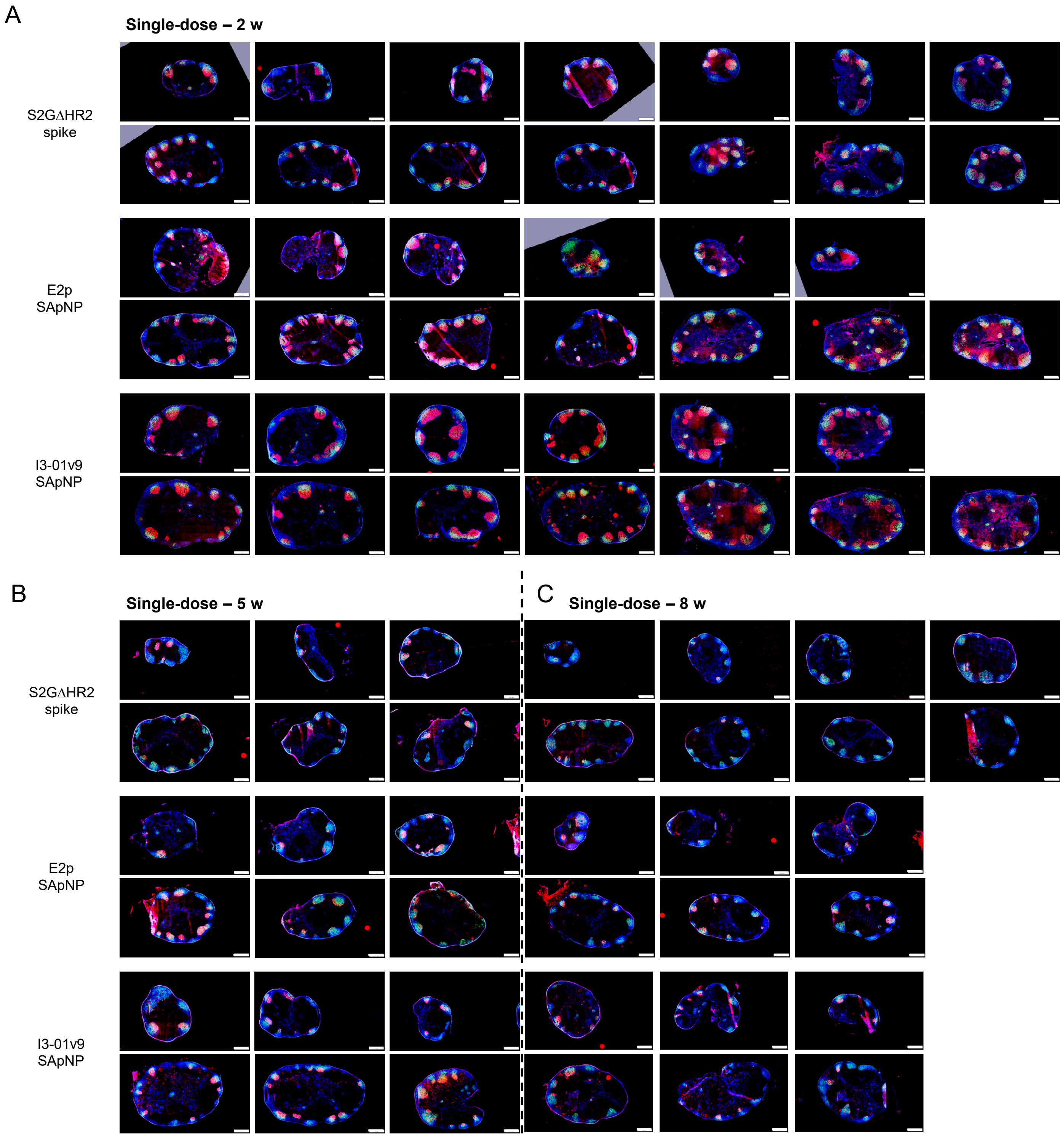
A
Single-dose – 2 w
S2GΔHR2 spike
E2p SApNP
I3-01v9 SApNP
B
Single-dose – 5 w
C
Single-dose – 8 w
S2GΔHR2 spike
E2p SApNP
I3-01v9 SApNP

fig. S7

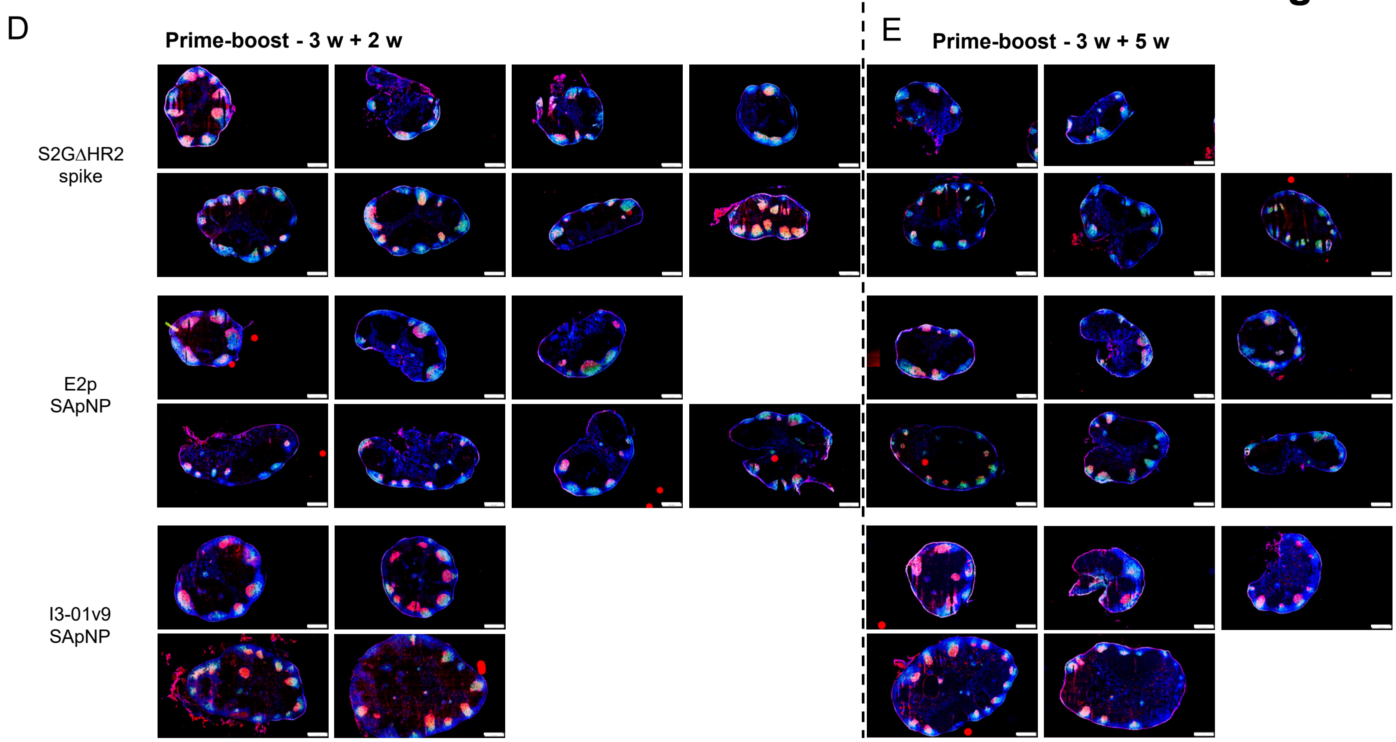

fig. S7. Immunohistological analysis of SARS-CoV-2 spike/spike-presenting SApNP vaccine-induced GCs. Images of germinal centers at (**A**) week 2, (**B**) week 5, and (**C**) week 8 after a single-dose injection of S2GΔHR2 spike and S2GΔHR2-presenting E2p and I3-01v9 SApNP vaccines (10 μg per injection, totaling 40 μg per mouse), with a scale bar of 500 μm for each image. Images of germinal centers at (**D**) week 2 and (**E**) week 5 after prime-boost injections.

fig. S8

A

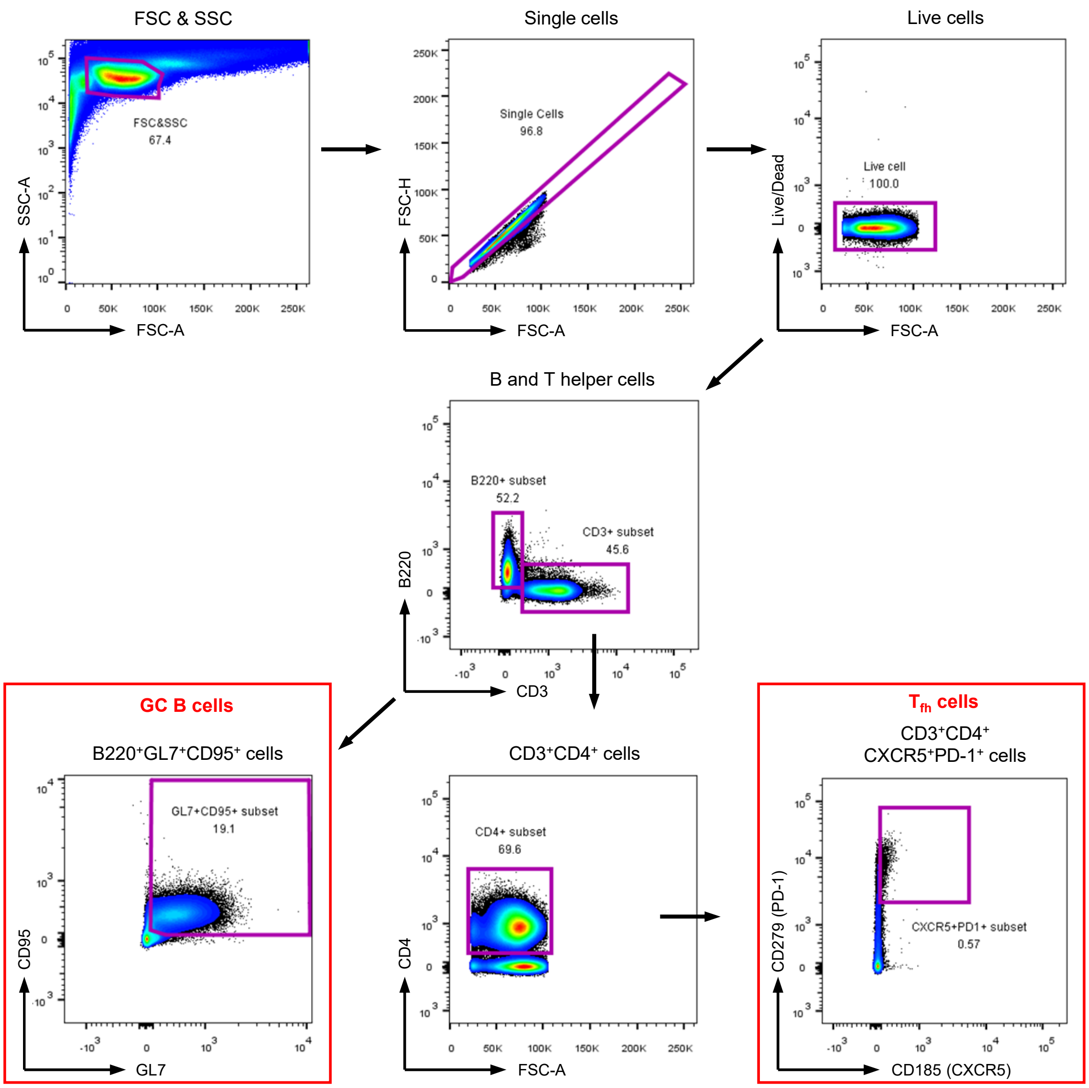
FSC & SSC
FSC&SSC
67.4
SSC-A
FSC-A
Single cells
Single Cells
96.8
FSC-H
FSC-A
Live cells
Live cell
100.0
Live/Dead
FSC-A
B and T helper cells
B220+ subset
52.2
CD3+ subset
45.6
B220
CD3
GC B cells
B220⁺GL7⁺CD95⁺ cells
GL7+CD95+ subset
19.1
CD95
GL7
CD3⁺CD4⁺ cells
CD4+ subset
69.6
CD4
FSC-A
Tfh cells
CD3⁺CD4⁺
CXCR5⁺PD-1⁺ cells
CXCR5+PD1+ subset
0.57
CD279 (PD-1)
CD185 (CXCR5)

fig. S8

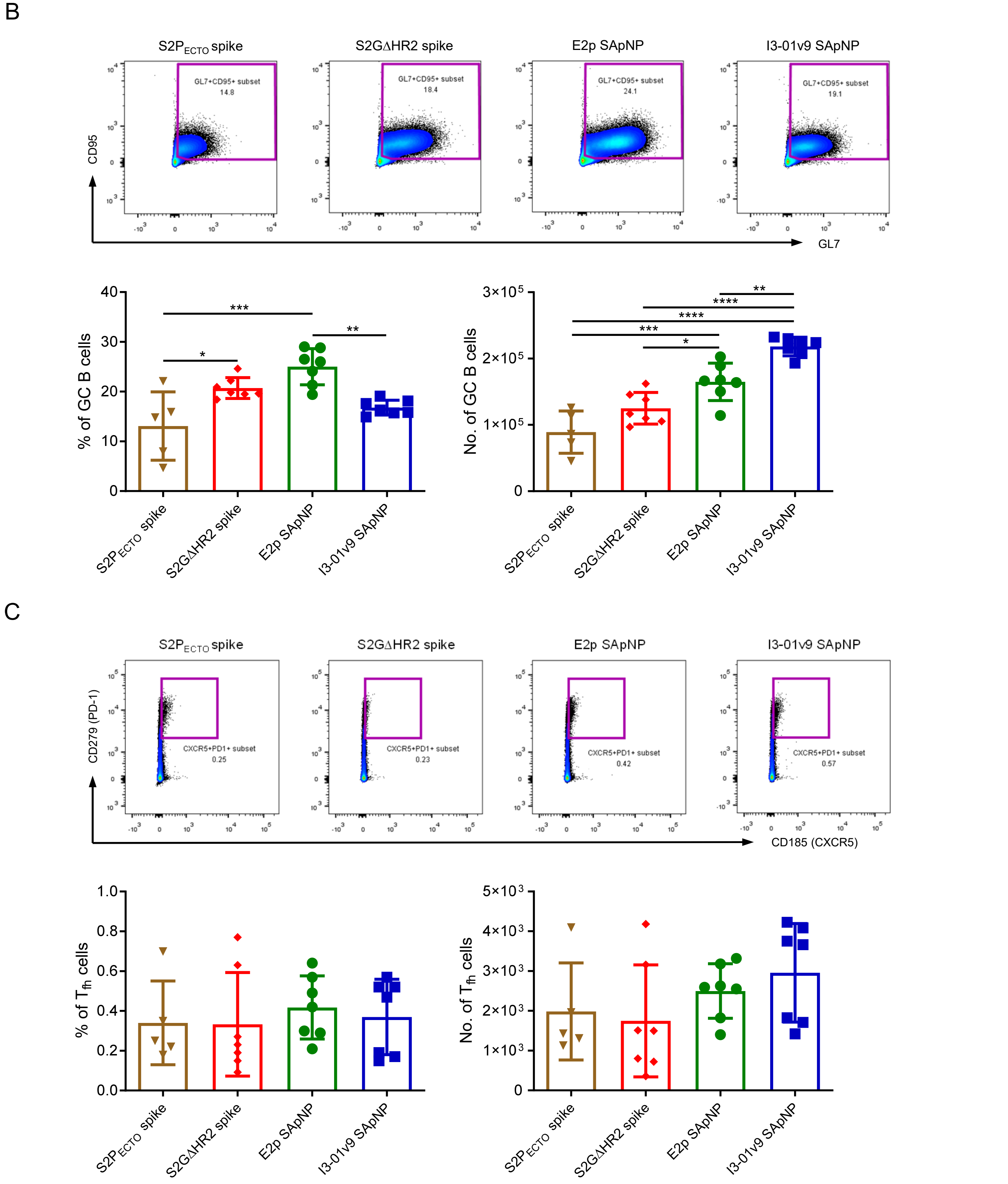

fig. S8. Flow cytometry analysis of SARS-CoV-2 spike/spike-presenting SApNP vaccine-induced GCs. (**A**) Gating strategy for analyzing germinal center reactions using flow cytometry. Quantification of germinal center reactions, including (**B**) GC B cells and (**C**) T follicular helper cells at week 2 after a single-dose injection of $S2P_{ECTO}$, S2GΔHR2, and S2GΔHR2-presenting E2p and I3-01v9 SApNP vaccines (10 μg per injection, totaling 40 μg per mouse)**.** Data points are presented as mean ± SD. The *P* values were determined by one-way ANOVA followed by Tukey's multiple comparisons *post hoc* test for each timepoint. **p* < 0.05, ***p* < 0.01, ****p* < 0.001, *****p* < 0.0001.

fig. S9

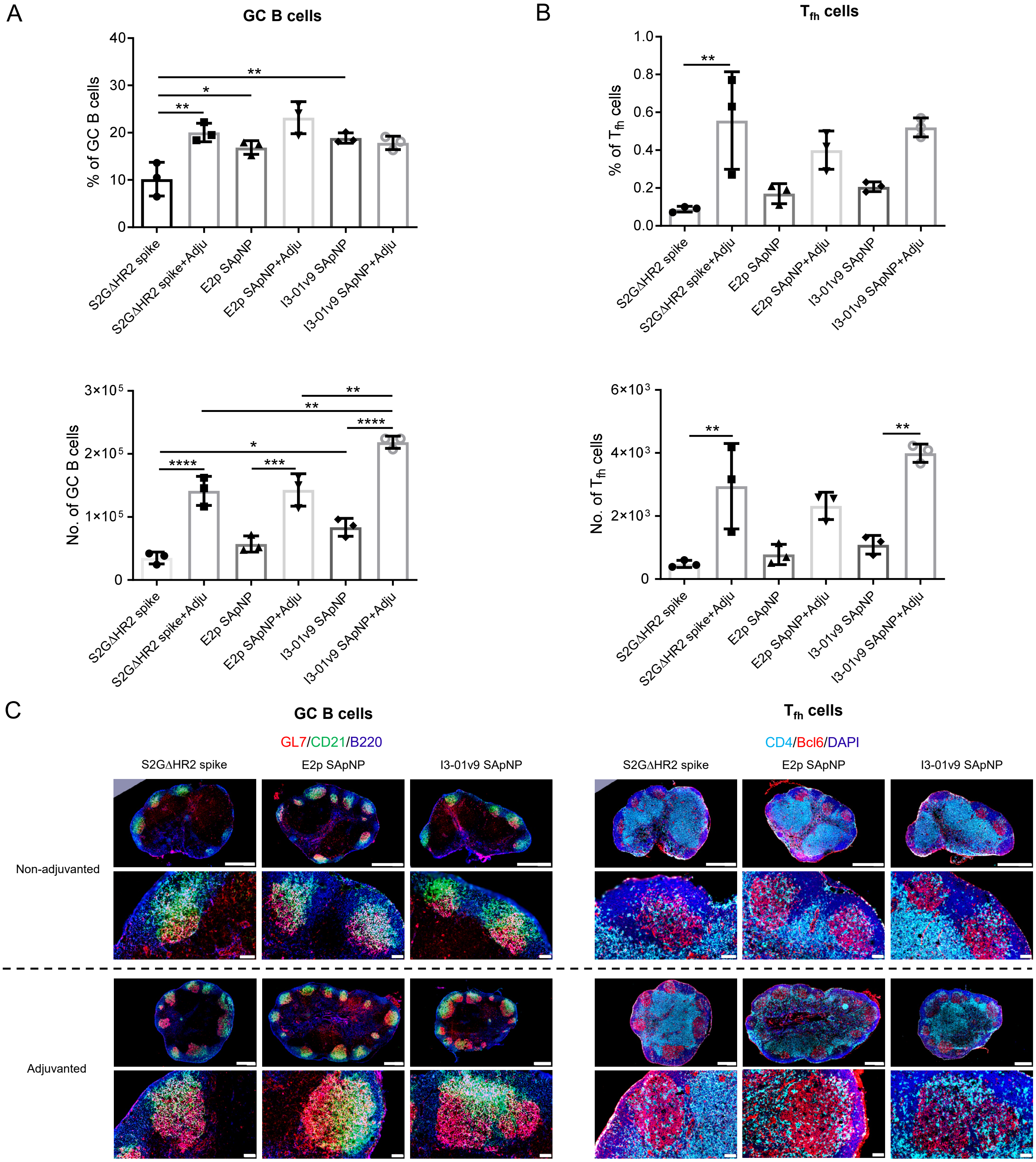

fig. S9. Adjuvant effect on SARS-CoV-2 spike/spike-presenting SApNP vaccine-induced GCs. Quantification of germinal center reactions, including (**A**) GC B cells and (**B**) T follicular helper cells at week 2 after a single-dose injection of S2P$_{ECTO}$, S2GΔHR2, and S2GΔHR2-presenting E2p and I3-01v9 SApNP vaccines with/without adjuvants using flow cytometry (10 μg per injection, totaling 40 μg per mouse). (**C**) Immunohistology of germinal centers at week 2 after a single-dose injection with/without adjuvants. Scale bars of 500 μm and 50 μm are shown for a complete lymph node and an enlarged image of a follicle, respectively. Data points are presented as mean ± SD. The *P* values were determined by one-way ANOVA followed by Tukey's multiple comparisons *post hoc* test for each timepoint. **p* < 0.05, ***p* < 0.01, ****p* < 0.001, *****p* < 0.0001.

fig. S10

A

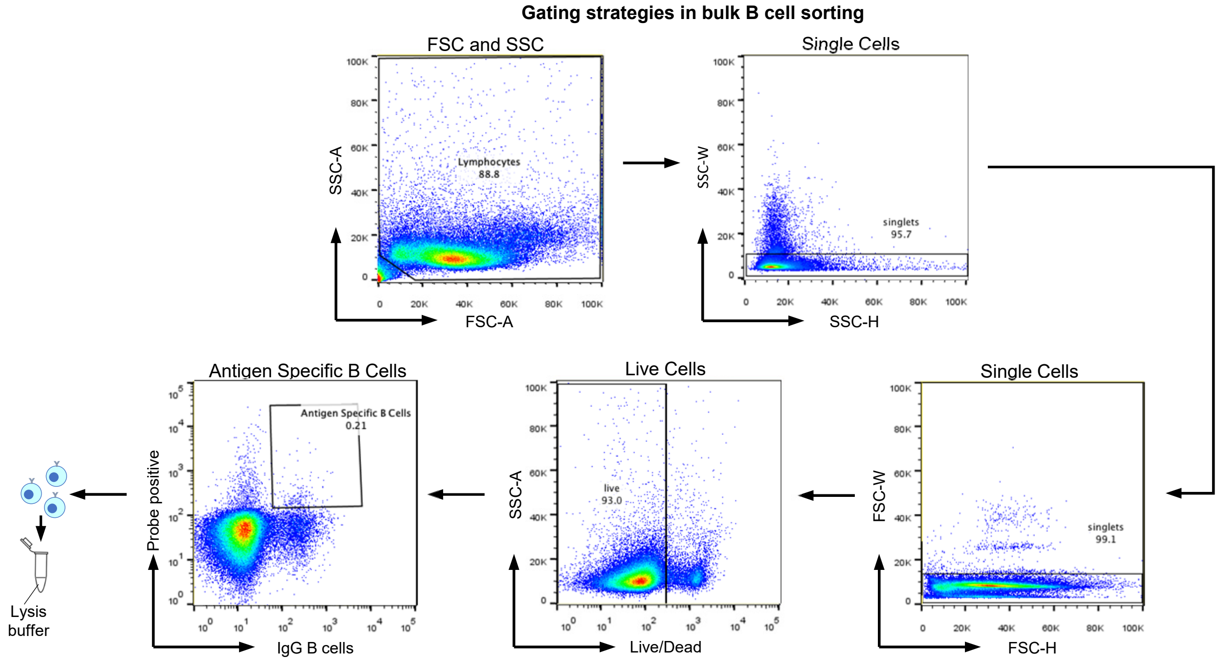

SARS-CoV-2 S2GΔHR2-specific sorting of mouse lymph node (LN) B cells from 3 low-dosage vaccine groups [a]

S2GΔHR2-5GS-1TD0			S2GΔHR2-5GS-E2p-LD4-PADRE			S2GΔHR2-5GS-I3-01v9-LD7-PADRE		
Mouse	Sorted B cells	%LN B cells	Mouse	Sorted B cells	%LN B cells	Mouse	Sorted B cells	%LN B cells
G5-1	487	0.07656	G6-1	670	0.08735	G7-1	1425	0.23039
G5-2	567	0.10521	G6-2	381	0.07638	G7-2	1196	0.24353
G5-3	358	0.07592	G6-3	314	0.05040	G7-3	1893	0.39877
G5-4	552	0.08819	G6-4	522	0.09089	G7-4	1164	0.23013
G5-5	442	0.07488	G6-5	297	0.05094	G7-5	1377	0.08676

[a] In this study, each mouse was immunized at w0 and w3 with a dosage of 3.3μg nanoparticle protein mixed with respective adjuvants. LN samples at w5 were processed for bulk sorting of S2GΔHR2-specific B cells to facilitate deep sequencing analysis.

B

Next-generation sequencing (NGS) analysis of 3 low-dosage SARS-CoV-2 vaccine groups [a]

Vaccine antigen	Mouse	N_{Raw}	$N_{V\text{-}assign}$	$N_{V\text{-}align\ (250bp)}$	Chain	N_{Chain}	N_{Step5}	<Length>	N_{Usable}
S2GΔHR2-5GS-1TD0 (Spike)	G5-1	1,596,202	441,528	99,119	H	49,456	49,347	361.3	49,347
					K	49,663	49,480	337.5	49,480
	G5-2	846,960	393,825	208,528	H	47,402	47,286	364.0	47,283
					K	161,126	160,748	330.8	160,747
	G5-3	843,754	289,986	71,301	H	27,115	270,12	362.0	27,010
					K	44,186	44,038	333.0	44,038
	G5-4 [b]	1,030,253	270,732	34,713	H	34,674	34,584	355.9	34,583
					K [c]	39	39	331.0	39
	G5-5	1,018,511	362,904	75,634	H	37,696	37,544	361.2	37,542
					K	37,938	37,851	336.3	37,851
S2GΔHR2-5GS-E2p-LD4-PADRE (NP)	G6-1	844,432	442,045	150,263	H	118,683	118,154	359.6	118,152
					K	31,580	31,338	340.6	31,338
	G6-2	743,730	391,866	118,081	H	31,763	31,681	359.5	31,677
					K	86,318	86,150	334.6	86,149
	G6-3	847,416	360,187	100,405	H	34,702	34,588	361.8	34,586
					K	65,703	65,385	343.5	65,385
	G6-4	804,742	325,463	148,519	H	57,260	55,248	358.1	55,245
					K	91,259	90,934	332.2	90,934
	G6-5	1,226,643	388,694	148,556	H	45,000	44,835	360.3	44,833
					K	103,556	103,386	328.5	103,385
S2GΔHR2-10GS-I3-01v9-LD7-PADRE (NP)	G7-1	698,174	561,918	196,631	H	84,704	84,373	359.5	84,371
					K	111,927	111,777	331.6	111,776
	G7-2	685,753	587,525	226,293	H	84,558	84,259	359.0	84,258
					K	141,735	141,506	332.1	141,505
	G7-3	772,549	645,323	233,227	H	85,301	84,944	359.1	84,944
					K	147,926	147,648	332.4	147,648
	G7-4	887,414	388,120	126,844	H	59,480	59,271	358.8	59,268
					K	67,364	67,232	330.4	67,232
	G7-5	537,471	283,727	103,400	H	37,814	37,329	358.8	37,329
					K	65,586	65,438	345.3	65,437

[a] Listed items include the vaccine antigen, mouse sample ID, number of raw reads from Ion S5 sequencing, number of reads that can be assigned to a VH/VK gene with an E-value of 10^{-3} or lower, number of reads that can be aligned to a VH/VK gene with 250bp or longer, number of VH/VK chains, number of VH/VK chains at the last step (5) of pipeline processing, average read length, and number of usable chains. Of note, to determine usable chains, the 250bp V-gene alignment filter was applied again to remove any problematic sequences detected during the full pipeline processing.

[b] Due to the difficulty in κ-light chain library preparation, only 39 reads were obtained for M4 sample in the S2GΔHR2-5GS-1TD0 group. The results from pipeline processing are provided here only for the reason of completeness.

fig. S10

C **S2GΔHR2-5GS-1TD0 spike group**

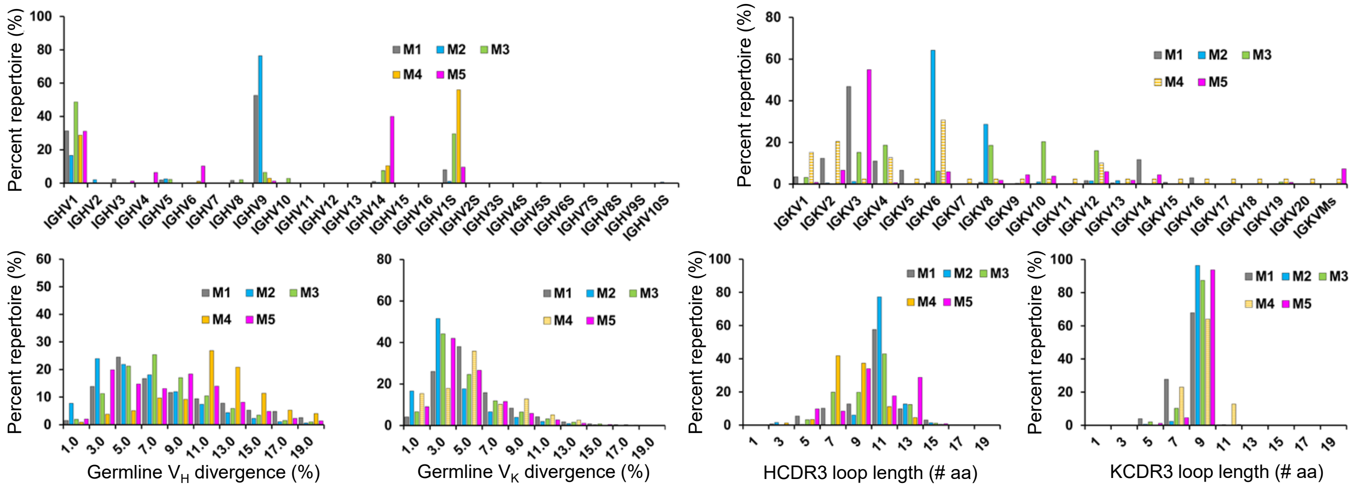

D **S2GΔHR2-5GS-E2p-LD4-PADRE NP group**

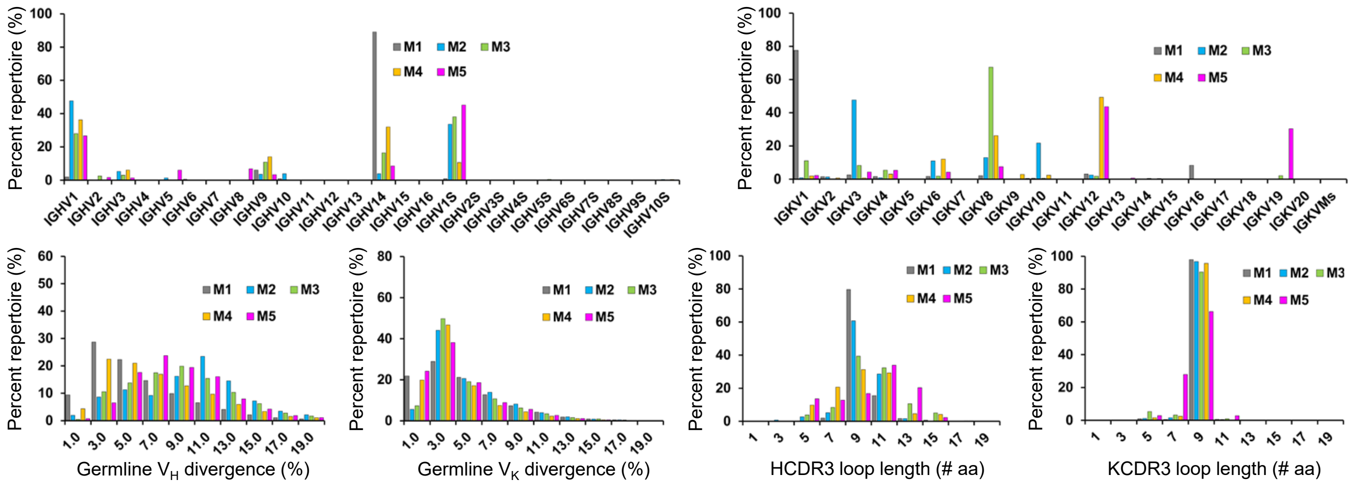

E **S2GΔHR2-10GS-I3-01v9-LD7-PADRE NP group**

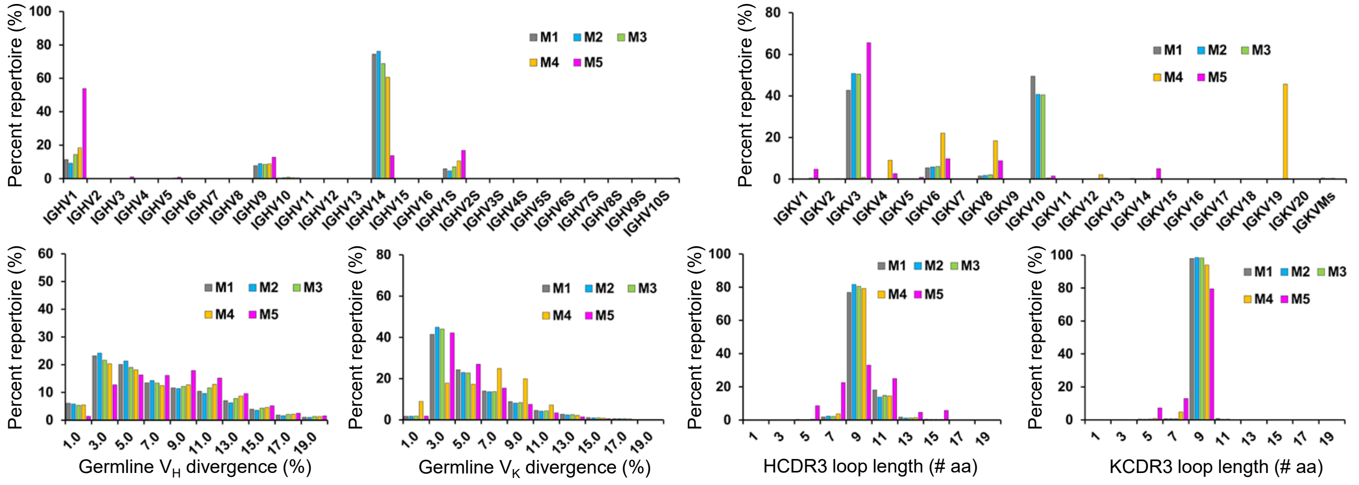

fig. S10

F

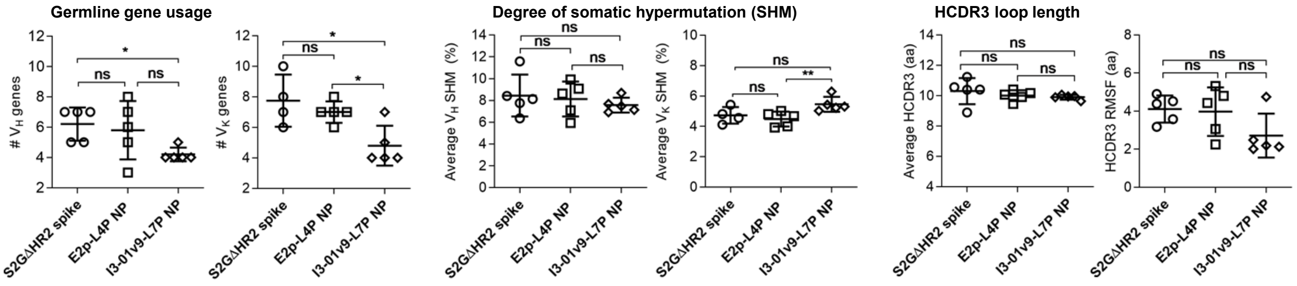

fig. S10. NGS analysis of SARS-CoV-2 spike-specific lymph node (LN) B cells from mice immunized with the spike and SApNP vaccines. In this study, three groups of mice were immunized with S2GΔHR2-5GS-1TD0/AddaVax, S2GΔHR2-5GS-E2p-L4P/AddaVax, S2GΔHR2-10GS-I3-01v9-L7P/aluminum phosphate (AP) at w0 and w3 via intradermal (i.d.) footpad injections (0.8 μg per injection site, totaling 3.3 μg per mouse). The spike probe in fig. S3A (left), S2GΔHR2-5GS-foldon-Avi-Biot, was used to sort mouse LN B cells. (**A**) Bulk B-cell sorting experiment. Top: gating strategies used in the antigen-specific sorting of mouse LB B cells (Step 1: remove cell debris; Steps 2 and 3: exclude clumped or sticky cells to ensure that only single cells remain; Step 4: remove dead cells; Step 5: identify antigen-specific B cells). Bottom: Summary of SARS-CoV-2 spike-specific bulk sorting of mouse LN B cells from three vaccine groups. Bottom: Summary of SARS-CoV-2 spike-specific bulk sorting of mouse LN B cells from three vaccine groups. (**B**) Antibodyomics analysis of repertoire NGS data obtained for spike-specific mouse LN B cells from three vaccine groups. B-cell repertoire profiles are shown for three groups of mice immunized with (**C**) S2GΔHR2-5GS-1TD0, (**D**) S2GΔHR2-5GS-E2p-L4P, and (**E**) S2GΔHR2-10GS-I3-01v9-L7P. Top: VH gene usage (left) and VK gene usage (right); Bottom: germline VH/VK divergence (left) and CDRH/K3 loop length (right). (**F**) Cross-group comparison of key repertoire properties. Left: statistical comparison of the number of activated VH/VK genes (≥ 1% of the total population); middle: statistical comparison of the average VH/VK SHM rate; right: statistical comparison of HCDR3 loop length and root-mean-square fluctuation (RMSF) of HCDR3 loop length. The RMSF value is used as an indicator of how much HCDR3 loop length varies within the spike-specific antibodies from each mouse. In statistical comparison, mean value and standard deviation (SD) are shown as black lines. *P*-values were determined by an unpaired two-tailed *t* test in GraphPad Prism 9.1.2. The asterisk symbol (*) indicates the level of statistical significance: ns (not significant), $*p < 0.05$, $**p < 0.01$. Of note, for M4 of the S2GΔHR2-5GS-1TD0 group, only 39 reads were obtained from Ion S5 sequencing due to the difficulty in light chain library preparation, as shown in the summary in (B). Therefore, the light chain profiles for M4 are only shown for the sake of completeness in (C) and will not be included in the comparison between different vaccine groups in (F).
